# Identical-location electron microscopy for the discovery of heterogeneous catalysts' dynamics at the single-particle level

**DOI:** 10.1039/d6cs00998k

**Published:** 2026-10-22

**Authors:** Yifan Chen, Jesum Alves Fernandes, Andrei N. Khlobystov

**Affiliations:** a School of Chemistry, University of Nottingham NG7 2RD UK Andrei.Khlobystov@nottingham.ac.uk

## Abstract

Understanding how heterogeneous catalysts evolve at the single-particle level is central to advancing modern industrial chemistry and energy conversion technologies. However, conventional characterisation techniques provide only ensemble-averaged information, obscuring the structural diversity and dynamic behaviour that govern catalytic performance. In particular, individual nanoparticles can follow distinct evolution pathways under reaction conditions, making it challenging to establish clear structure–property relationships. Identical-location electron microscopy (IL-EM) has emerged as a powerful approach to overcome these limitations by enabling the direct tracking of the same catalyst particle across different stages of a reaction. By preserving spatiotemporal continuity, IL-EM provides particle-resolved insights into single-particle events such as coalescence, dissolution, restructuring, and compositional changes, while avoiding radiolysis damage during the reaction stage. This capability allows reconstruction of catalyst evolution pathways with atomic-scale precision under realistic operating conditions, without the need for specialised EM equipment or software. This review critically examines recent advances in IL-EM methodologies, including experimental workflows, correlative spectroscopy, and applications in thermal and electrochemical catalysis, as well as emerging applications for photocatalysis. Particular emphasis is placed on how IL-EM reveals mechanistic insights that are inaccessible through conventional ensemble-averaging techniques, and workflows correlating IL-EM with other local-probe and macroscale characterisation methods. Finally, we discuss current challenges, including limited time resolution, experimental labour intensity, and statistical considerations, alongside emerging opportunities, such as automation, artificial intelligence (AI)-assisted analysis, and multimodal characterisation. These developments are expected to transform IL-EM into a high-throughput and widely accessible tool. As such, IL-EM is poised to play a key role in establishing quantitative design principles for next-generation heterogeneous catalysts.

## Abstract 2

Identical-location electron microscopy enables particle-resolved tracking of structural evolution, providing deep insights into catalyst stability and the structure–function relationship beyond what ensemble-averaged analysis can offer.

## Graphical abstract

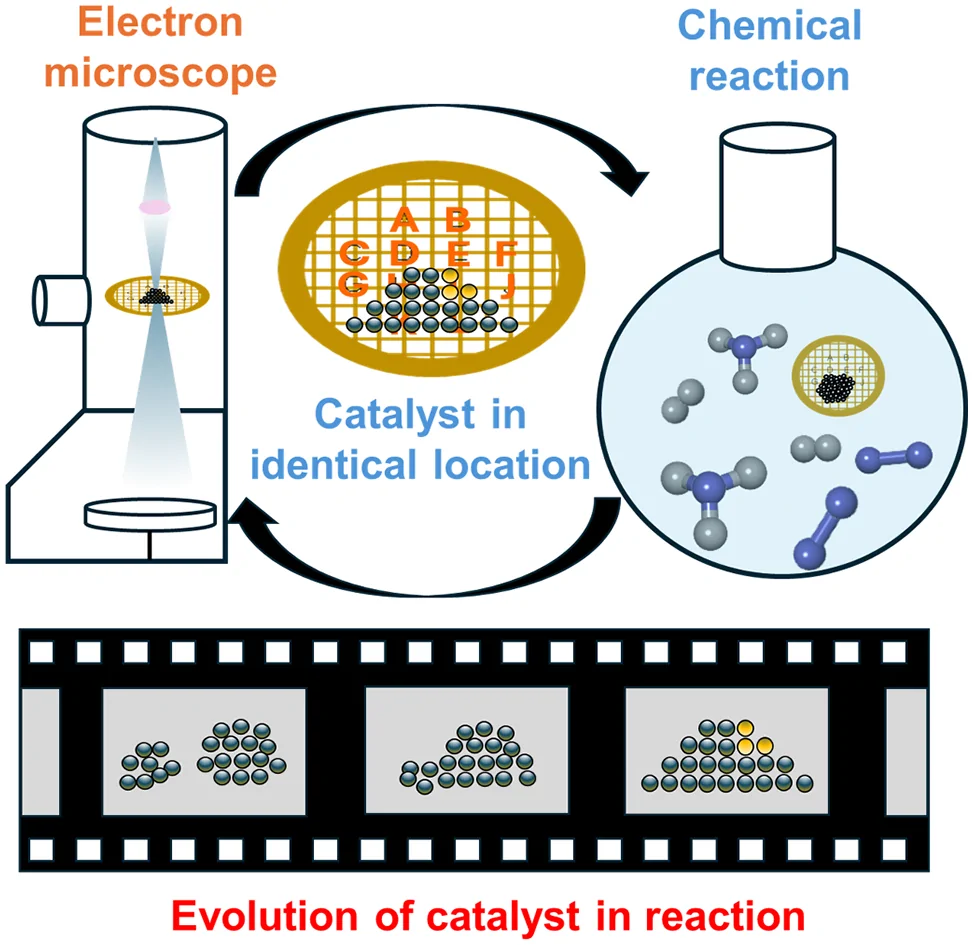

## 1. Introduction: tracking the chemistry of a single particle

Heterogeneous catalysis is a crucial component of the chemical industry, responsible for over 70% of chemical reactions used to produce solvents, fuels, fertilisers, polymers, pharmaceuticals, paints, adhesives, and many other products.^1^ For catalysts to facilitate molecular transformations driven by heat, light, or electric potential, they must exhibit high activity (typically measured by turnover frequency, ToF; current density; or quantum efficiency), selectivity for the desired product, and stability under operational conditions.^2–5^ Meeting all these criteria simultaneously is quite challenging. This challenge can only be addressed by understanding the inner workings of catalyst particles and linking atomic-scale dynamics to macroscopic reaction kinetics. If we can successfully connect the atomic-scale behaviour of a catalyst to its overall performance, it would help establish design rules for creating new, highly effective catalysts. This could accelerate the search for efficient catalysts and improve the success rate of discoveries in chemistry.

The main challenge in this process is two-fold. First, typical heterogeneous catalysts – especially those used in industrial applications – are highly polydisperse. This means that particle sizes, shapes, and compositions (in the case of multi-metallic particles) vary, making each particle unique. Second, catalyst particles are not static; they undergo complex nanoscale transformations in response to reactant molecules and external conditions throughout activation, steady-state operation, and deactivation.^6,7^

Bulk analysis methods, such as spectroscopy and diffractometry, offer averaged information about a group of catalyst particles. As a result, the atomic structures of individual catalyst particles and the mechanisms of their evolution during catalysis must be inferred from modelled experimental data rather than observed directly. In contrast, local-probe methods of analysis, such as electron microscopy (EM), provide a direct view of the behaviour of individual particles. This is essential for understanding heterogeneous catalysts, as different particles display varying dynamics. The primary goal of most microscopy investigations in catalysis is to determine the relationship between a particle's initial state and its subsequent evolution mechanism. A secondary goal is to connect these findings to the catalyst's performance. The overall performance of the catalyst observed at the macroscale is a function of the total sum of all single-particle events, SPEs (coalescence, faceting, Ostwald ripening, alloying or dealloying, carburisation or nitridation, poisoning, leaching, and even coulombic explosion).^6^ Each SPE is normalised by its occurrence frequency, *n*_*i*_ (Fig. 1c), and finding those frequencies requires multiple measurements to reconcile spatial resolution and statistical significance in EM observations. Only when the SPEs and their frequencies are known can one be confident to propose atomistic mechanisms responsible for the heterogeneous catalyst performance measured in bulk-chemistry experiments.

**Fig. 1:**
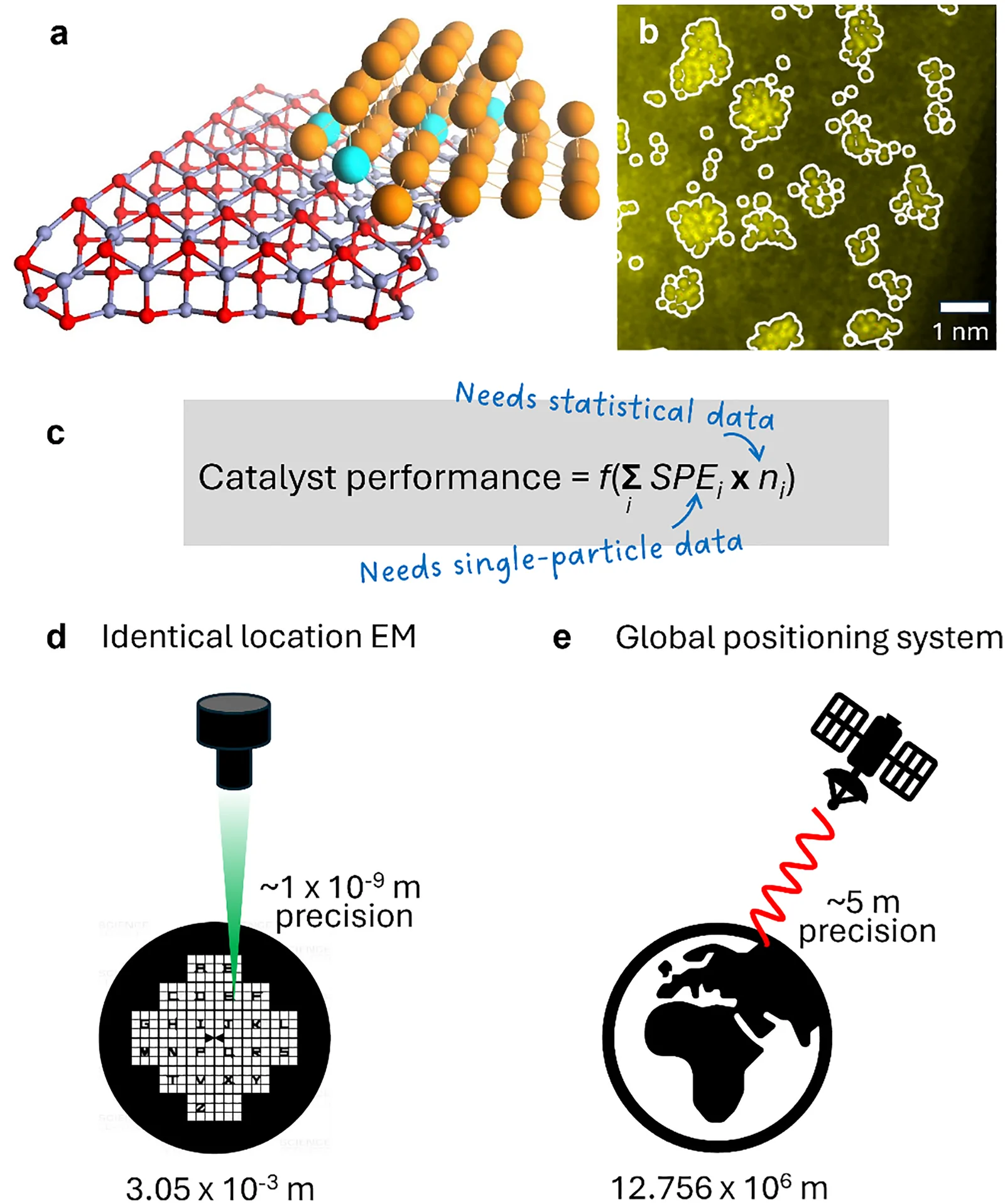
Heterogeneous catalysts are often viewed as idealised particles with well-defined, uniform structure (a), but the reality is far from this, as demonstrated here by an aberration corrected scanning transmission electron microscopy (AC-STEM) image of a typical heterogeneous catalyst (Ru on carbon; b). The overall catalyst performance is a function of the sum of single-particle events (SPE) normalised by the frequency of their occurrence (*n*_*i*_, c). (d) and (e) Comparative precision of atomic-scale relocation in IL-EM *versus* planetary-scale navigation *via* GPS.

Transmission electron microscopy (TEM) and scanning electron microscopy (SEM) offer spatial resolutions from microns to nanometres to sub-Angstroms, thus enabling access to the full range of length scales relevant to heterogeneous catalysis. In particular, structural characterisation of catalyst particles at the nanoscale can provide indispensable information, such as size, composition, shape, crystallinity, strain, and defects.^8^ This is ideal for identifying SPEs, but the key to success lies in the imaging protocol. Average information gained from imaging random sets of particles, before and after catalysis, can be useful for understanding general trends in catalyst dynamics, but it cannot unambiguously identify SPEs because the identity of individual particles is not preserved between successive imaging stages. Such averaged microscopy information is akin to bulk characterisation methods and thus requires good statistics derived from imaging large numbers of particles across different randomised locations within the sample.

Identical-location electron microscopy (IL-EM) has been developed to preserve the identity and spatial correspondence of individual particles across successive reaction stages, enabling analysis of the specimen before and after each step of catalysis at the location of the same particles. This procedure usually uses a TEM finder grid as the sample holder, with grid squares alphanumerically labelled, but the grid bars or mask pattern of a conventional TEM grid can also serve as a usable coordinate system for identifying the same region. Generally, the catalyst is first imaged and characterised before exposure to any reactions. Then the grid is transferred to real reaction conditions in a reactor, after which it is imaged and analysed in the microscope again. To gain a detailed understanding of catalyst evolution at different stages of the reaction, the procedure can be repeated multiple times. This approach can answer crucial questions that ensemble-averaged EM cannot. For example, pioneering work by Mayrhofer and Arenz^9^ using IL-TEM has shown that Pt particles detaching from the support are not re-deposited and instead dissolve into the electrolyte during the applied harsh conditions.

IL-EM utilises the full potential of the microscope for imaging and analysis. Unlike a liquid- or gas-cell *in situ* TEM, there is no compromise on spatial resolution or chemical information due to electron-beam scattering by window materials of the cell enclosing the catalyst. A variant of *in situ* TEM, environmental TEM (ETEM), also bypasses the challenge of the cell window by creating a reaction environment inside the TEM column using rarefied gases. In contrast to ETEM, the reaction conditions experienced by the catalyst in IL-EM workflows are identical to those in the actual chemical reactor. Importantly, interaction between the electron beam and matter is more complex when imaging a catalyst directly in reaction conditions, resulting in various effects such as direct momentum transfer to atoms, ionisation, heating, and radiolysis of the reaction medium, with each depending intricately on the electron beam's energy and flux.^10^ Indeed, there is no single solution that can mitigate beam damage on the catalyst imaged during a chemical reaction; addressing one type of beam damage often exacerbates another, such that the electron beam not only interferes with *in situ* reaction observations but may completely override the natural behaviour of atoms and molecules under thermal, electrochemical, or photocatalytic conditions during *in situ* experiments.^11–14^ In the IL-EM protocols, the catalyst operates under actual reaction conditions while being completely shielded from the electron beam during the reaction. This permits a detailed examination of the catalyst's structure and composition, enabling the reconstruction of an atomistic evolution pathway at the single-particle level from successive IL-EM images acquired after defined reaction stages. Table 1 summarises the key advantages and disadvantages of different EM approaches.

**Table 1:** Summary of the key advantages and disadvantages of different EM approaches

Methods^15,16^	Pros	Cons
ETEM	• Enables real-time observation of materials in gases and at elevated temperatures	• Limited gas pressure compared to realistic reaction catalytic conditions
	• Gas is admitted directly into the specimen region, without any cells/windows	• Restricted gas types
	• Direct imaging of gas-solid interactions, catalytic reactions, and oxidation/reduction processes	• Complicated operation and high cost

Closed-cell liquid-/gas-cell *in situ* TEM	• Dynamic insights into structural and morphological changes under different conditions (*e.g.*, temperature, electrical bias, mechanical stress, gas/liquid environment)	• Imaging resolution is often affected by the windows of the cell and/or solvent
	• Allows concurrent product analysis *via* mass spectrometry	• Solvent radiolysis makes it difficult to carry out liquid-phase reactions over extended time periods
	• Higher pressure compared to ETEM, but still below industrial catalytic reaction conditions	• Beam damage can change/override the intrinsic chemistry to be studied

Identical-location TEM	• No special instrumentation or dedicated EM required	• No real-time dynamics as catalyst evolution between imaging stages must be interpolated
	• Catalysis conditions (*e.g.*, pressure, temperature, concentration of reactants, electric potential, *etc.*) replicate real-world reactions	• Labour- and time-consuming if extended temporal catalyst dynamics are required
	• Reactions can be paused at any stage (*e.g.*, catalyst activation, steady-state operation, change of selectivity, degradation of activity, *etc.*) on demand, and the catalyst can be investigated in an arrested state	• Possible interactions between the TEM finder grid and the reaction environment should be considered
	• The electron beam does not interfere with *ex situ* reactions	• Increased likelihood of mechanical damage to the TEM sample due to human error
		• Loss of the native solid–liquid or solid–gas interface upon removal from the reaction environment

Importantly, IL-EM is not limited to imaging; it also leverages EM-based spectroscopy and diffractometry. For instance, energy-dispersive X-ray spectroscopy (EDS) analysis is commonly utilised in IL-EM studies to confirm the presence of elements and to monitor changes in their ratios within the catalyst particles and the support material. Similarly, electron energy loss spectroscopy (EELS) is often used to detect changes in the valence or oxidation state of metals following catalytic reactions. Indeed, both *in situ* and IL-EM workflows can benefit from EDS and EELS, which are integral parts of the EM instrumentation, with the added advantage that spectroscopy in IL-EM is unaffected by the materials of the cells’ windows, thereby enhancing the sensitivity.

Additionally, *ex situ* surface spectroscopies, such as X-ray photoelectron spectroscopy (XPS) or Raman spectroscopy, can be performed on the catalysts as part of the IL-EM workflow. This can conveniently be done on the same finder grid as the IL-EM imaging and the same stages of the reaction, thus providing precise correlation between imaging and *ex situ* spectroscopy. However, because these *ex situ* surface spectroscopy methods utilise a much larger probe size, they yield ensemble-averaged information. While this type of data lacks the spatial resolution to reveal the evolution of individual catalyst particles, it still provides complementary insights to enhance IL-EM measurements. Notably, correlative electrocatalysis - IL-EM investigations can be particularly informative, thanks to the conducting nature of TEM finder grids made of gold that can be directly integrated into the working electrode of the electrochemical cell between EM imaging sessions. This allows for the direct assessment of the redox behaviour and electrocatalytic performance of particles on the same grid used for IL-EM imaging. As a result, IL-EM has become a particularly popular technique for investigating electrocatalysis, as apparent from the literature covered in this review.

The main downside of IL-EM is that while it preserves spatiotemporal continuity by enabling the same catalyst particles to be relocated after successive reaction stages, it lacks time resolution as compared to *in situ* EM. In effect, while IL-EM provides snapshots of catalyst dynamics, *in situ* EM gives a movie. Main pros and cons of IL-EM compared to other approaches, including *in situ* EM and ETEM, are listed in Table 1.

## 2. IL-EM methodologies and experimental workflows

When we have a gram of molecules, such as water, they all share the same shape, size, and composition. In contrast, if we have a gram of nanoparticles, each one is different –some may vary only slightly, while others can differ significantly (Fig. 1a), depending on how they were produced. Additionally, because matter at the nanoscale does not exist at the thermodynamic minimum, nanoparticles made of the same material (for example, the same metal) can react and change in markedly different ways. This variability is heavily influenced by factors such as their initial size and shape, as well as local conditions like bonding with a support material, surface defects, and fluctuations in temperature, pressure, or the concentration of reactive species.

The primary goal of IL-EM is to monitor the evolution of individual particles under changing conditions and within their local nanoscale environments. In IL-TEM, each particle is treated as an individual entity and tracked over time to reveal its specific structural response to external stimuli, while accounting for the unique local environment surrounding the nanoparticle. This approach is experimentally demanding, yet IL-EM exploits the advanced capabilities of modern EM instrumentation, which can repeatedly relocate the same region of a microscopic sample with atomic precision. For example, a TEM grid with a diameter of approximately 3 mm can be revisited with atomic-scale accuracy. In relative terms, the precision of relocation is comparable to 5-meter GPS accuracy across the Earth's diameter (12 756 km), but accomplished with electron optics rather than satellite-based positioning systems (Fig. 1d and e).

Choosing the appropriate TEM grid is crucial for this process. It usually includes a set of fiducial marks or an alphanumeric-labelled grid to assist in navigating the micron scale when relocating the particle, similar to traditional atlas maps that use a square grid to find specific streets and houses in a large city. Equally important is the choice of support materials, whether for a TEM grid or SEM stub. The material must be stable and inert under reaction conditions so that it does not interfere with the catalysis during the sample's exposure to the reaction environment. Additionally, when the support forms part of an electric circuit – such as in IL-EM of an electrocatalyst – high electrical conductivity becomes an essential requirement.

There are different methods for integrating catalyst particles onto a TEM grid. One option is to deposit a prepared catalyst onto the grid using techniques such as drop-casting. Alternatively, the catalyst can be formed directly on the grid, which can lead to a more uniform distribution of nanoparticles (Fig. 2). This uniformity is beneficial for IL-EM imaging and is particularly useful when the same technique is applied to prepare a bulk catalyst as for EM samples. Surface assembly of the catalyst from atoms, using, for example, magnetron sputtering, is one effective method that can be used for both EM sample and bulk catalyst fabrication.^6,7,17–21^ This approach has the added benefit of cleanliness, as it is a solvent- and reagent-free method, which is crucial for high-quality EM imaging. Even minor surface impurities, such as leftover reagents, ligands, trapped solvents, or anions, can severely hinder microscopy imaging. This contamination must be avoided at all costs to ensure optimal EM data acquisition.

**Fig. 2:**
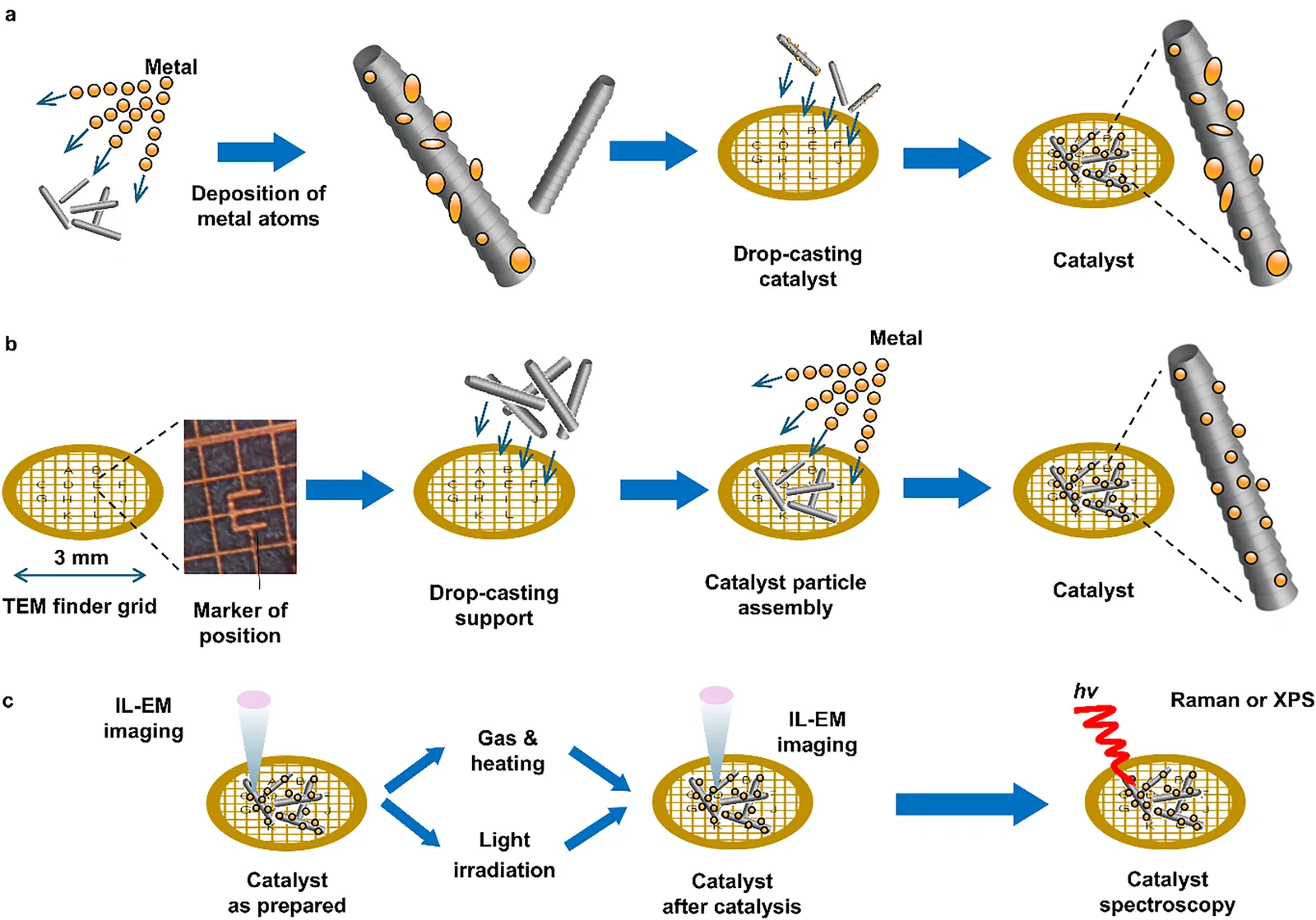
(a) Schematic illustration of a conventional catalyst sample preparation workflow, where the catalyst is assembled as bulk materials synthesis followed by deposition on a TEM finder grid. (b) Workflow for catalyst assembly directly on a TEM finder grid, where deposition of support is followed by assembly of metal particles by metal delivery, for example *via* magnetron sputtering, yielding a more uniform sample. (c) Schematic representation of the IL-EM workflow to probe catalyst evolution under thermally or photocatalytically driven reaction conditions, with optional follow-up characterisation by Raman spectroscopy or XPS.

After imaging the as-prepared catalyst on the TEM grid or the SEM stub in the required number of areas, it can be placed in a reactor alongside the bulk catalyst. Alternatively, it can be exposed to the same conditions as the bulk catalyst separately or connected to the working electrode in the case of electrocatalysis. For the latter, this connection can be established simply using a piece of wire, tweezers, or a cap, or with a more sophisticated floating electrode assembly. When testing electrocatalysts under fuel cell conditions, the EM sample can be sandwiched between the cathode and anode plates. Regardless of how the EM sample is connected to the electrical circuit, ensuring high-quality contact between the EM sample and the working electrode, as well as with the electrolyte solution, is crucial. The latter can be particularly challenging due to the formation of gas bubbles, which can interfere with measurements and disrupt the realistic reaction environment around the catalyst. Despite these challenges, IL-EM for electrocatalysts is currently very popular. This popularity is likely driven by the ease of measurement, because the TEM grid can be easily integrated into a working electrode. Additionally, due to the highly sensitive nature of electrochemical equipment, the electrochemical response of the catalyst (*e.g.*, current density or overpotential) can be directly recorded for the EM sample under reaction conditions (Fig. 3).

**Fig. 3:**
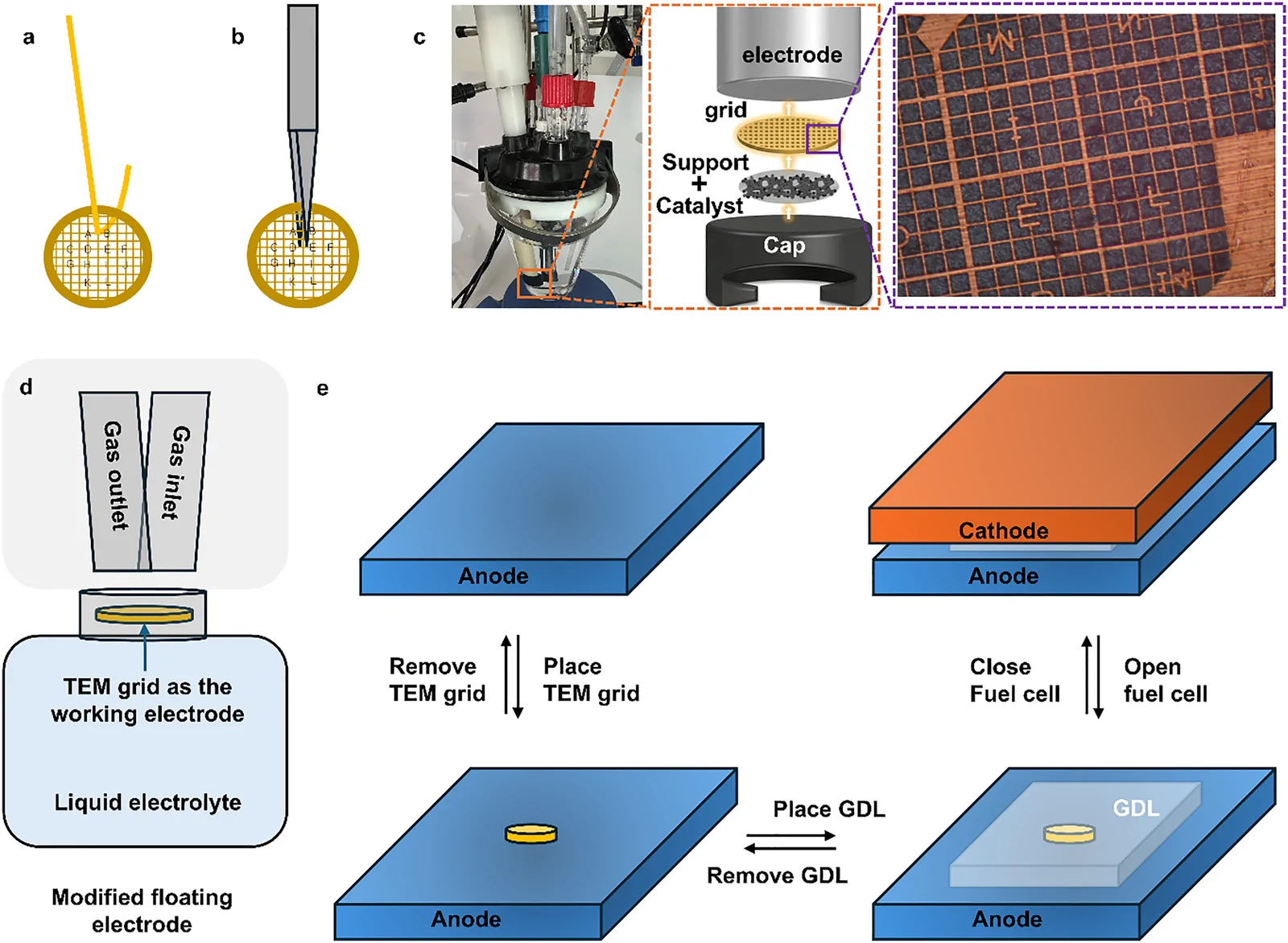
Schematic overview of different approaches for establishing electrical contact between a TEM finder grid and the working electrode: (a) connection *via* a conductive wire or (b) tweezers, and (c) attachment to a glassy carbon rod combined with a 3D-printed Teflon cap. (d) A floating electrode configuration including a TEM finder grid. (e) Illustration of the IL-EM strategy implemented in a fuel cell system, where GDL denotes the gas diffusion layer.

Another downside of IL-EM workflows compared with *in situ* TEM is that the reaction must be interrupted. Once the reaction is halted by removing the source of activation – such as heat, light, or electric potential – the catalyst enters an arrested state. This arrested state must be protected from the negative influence of the ambient environment (*e.g.* O_2_ of the air) during extraction from the reactor and transfer onto a TEM or SEM holder. Manual handling of samples between exposure to reaction conditions and EM analysis increases the risks of sample contamination and damage that need to be carefully managed; another disadvantage as compared to *in situ* EM. However, the interruption of the reaction also provides an opportunity to incorporate other *ex situ* measurements into the IL-EM workflow, such as XPS or Raman spectroscopy, as illustrated in several examples below. These measurements must also be performed with caution to avoid damaging the sample. The issue of electron beam damage is significantly reduced in IL-EM workflows compared to *in situ* and *operando* microscopy, as the catalyst is free from reactant and solvent molecules, which are more susceptible to beam damage and can generate highly reactive species (*e.g.*, cation radicals) that may chemically etch the catalyst when the electron beam is on. Nevertheless, even when the catalyst sample is placed in a vacuum, electron-beam damage, such as knock-on effects, sample ionisation, or heating, can still occur. Fortunately, the thin nature of catalyst particles helps to mitigate heating; however, sample ionisation remains a concern, particularly if the catalyst support is an insulator, such as metal oxides. Knock-on damage, caused by momentum transfer from the electrons in the beam to the catalyst atoms, is particularly prevalent and must be mitigated by reducing the electron beam energy. However, this reduction can lead to increased ionisation and heating, as these factors are inversely related to the electron beam energy. A widely accepted approach to controlling beam damage is low-dose EM, which helps address all types of damage simultaneously, with new direct electron detectors playing an important role in this. It is also good practice to check for sample deterioration caused by repeated identical-location imaging by inspecting fresh, unirradiated areas of the sample for comparison.

The challenges and limitations of IL-EM (Table 1) must be borne in mind when considering whether IL-EM is a suitable experimental method for a particular catalyst. While there is no universal recipe for successful IL-TEM imaging, as each catalyst presents unique challenges, a comprehensive workflow for conducting reliable identical-location scanning electron microscopy (IL-SEM) experiments has recently been reported,^22,23^ with similar good-practice protocols also emerging for identical-location scanning transmission electron microscopy (IL-STEM).^24^ These reports establish systematic experimental protocols that enable the reproducible re-location and analysis of the same microscopic region across multiple stages of treatment and characterisation.

## 3. IL EM in electrochemical energy systems

### 3.1. Electrochemical CO_2_ reduction reaction

#### 3.1.1. Single-metal catalysts

Copper is one of the most effective catalysts for the electrochemical conversion of CO_2_ to value products. The process of electrochemical CO_2_ reduction reaction (CO_2_RR) is complex and still hotly debated, both from the point of view of reactive intermediates formed during the reaction and the changes in the catalyst. The former is particularly important as they clearly affect the activity as well as the selectivity of the catalyst. IL-EM has been demonstrated as a powerful tool to track changes in the catalyst structure and composition during CO_2_RR. One of the important features of copper catalysts is that they usually have a layer of oxide covering the metal surface after preparation. The thickness of this layer and the composition of copper oxide (CuO_*x*_) depend heavily on the methods of catalyst preparation and pre-treatment before CO_2_RR.

For example, changes in 40 nm cubes of Cu coated with ∼2 nm of a native layer of CuO_*x*_ deposited on a gold finder grid have been investigated during CO_2_RR at −1.1 V *vs.* reversible hydrogen electrode (RHE) in 0.1 M KHCO_3_.^25^ IL-TEM imaging of the catalyst after 20 min of the reaction revealed etching of the facets of the Cu nanocubes so that the originally smooth sides became uneven, developing dents of several nm, which was linked to a change in selectivity of production, leading to a wide range of gas and liquid products (Fig. 4a–c). The authors determined the onset potential for the structural changes in the catalyst at −0.4 V *vs.* RHE and proposed a mechanism for cathodic copper dissolution. The cation of the electrolyte has been shown to play a critical role in the dissolution process by facilitating the formation of the soluble ternary copper hydride [CuH_3_]^−^, with all alkali cations, such as K^+^, Na^+^, Li^+^ and Cs^+^, facilitating the cathodic etching of the catalyst.

**Fig. 4:**
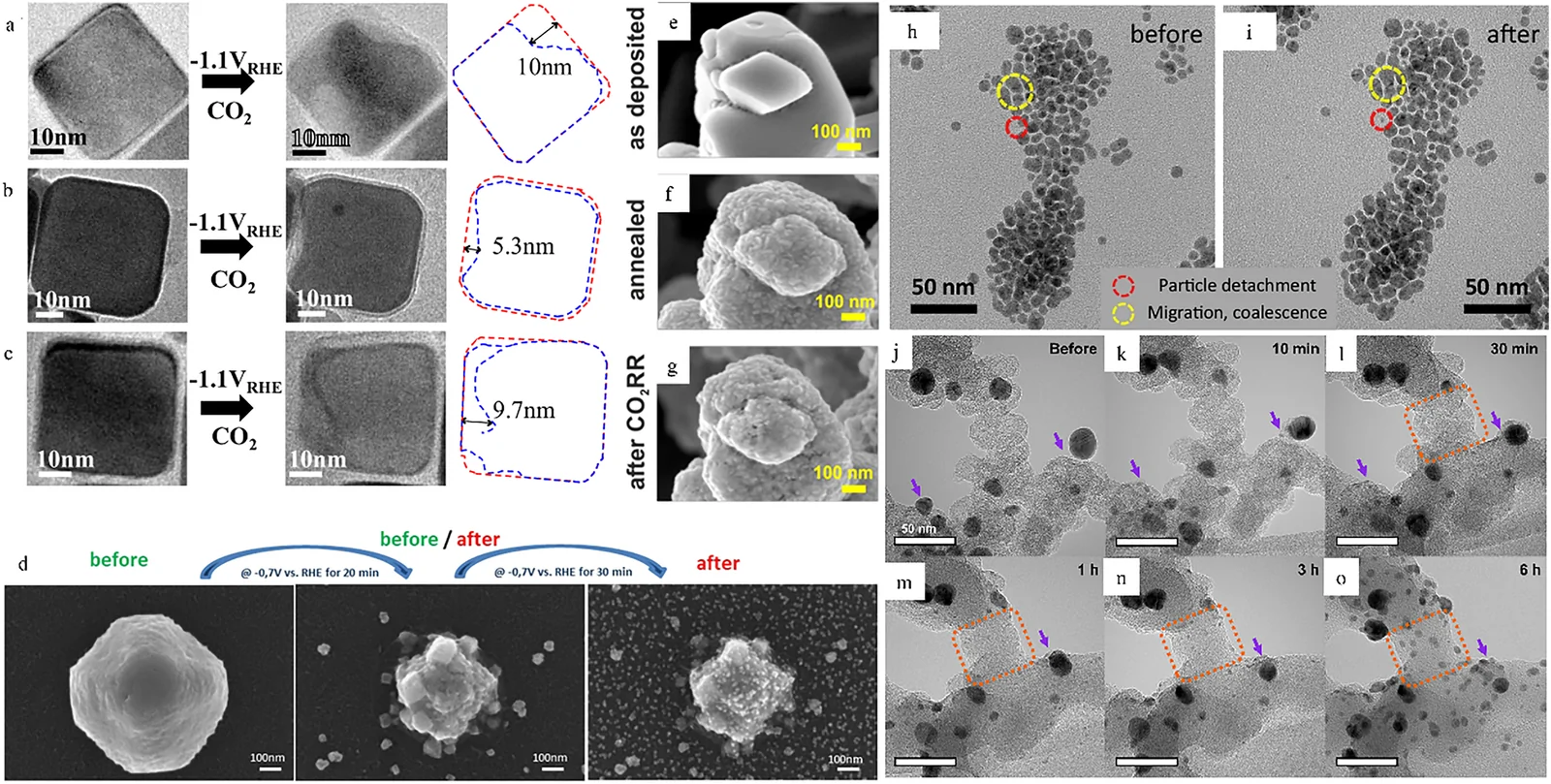
(a)–(c) IL-TEM inspection before (first column) and after (second column) treating the Cu nanocubes at −1.1 V *vs.* RHE in a CO_2_-saturated 0.1 M KHCO_3_ electrolyte for 20 min. Reproduced from ref. 25 with permission from Springer Nature, copyright 2024. (d) IL-SEM approach proving the dissolution/redeposition as the main degradation pathway of Cu nanoparticles. Reproduced from ref. 28 with permission from John Wiley and Sons, copyright 2021. (e)–(g) IL-SEM images of the Cu foam under different experimental conditions. Reproduced from ref. 30 with permission from Elsevier, copyright 2020. (h)–(i) IL-TEM measurement of the Ag nanoparticle catalysts (h) before and (i) after the CO_2_RR. Reproduced from ref. 33 with permission from Springer Nature, copyright 2024. (j)–(o) IL-TEM images of Ag nanoparticles at various reaction times (j) before, (k) 10 min, (l) 30 min, (m) 1 h, (n) 3 h and (o) 6 h of the CO_2_RR at −0.8 V *vs.* RHE. Reproduced from ref. 34 with permission from Elsevier, copyright 2021.

Copper catalysts in the form of foils, nanoparticles on different carbon supports, oxide-derived films, and gas diffusion electrodes have been systematically examined using IL-SEM under CO_2_ reduction conditions in KHCO_3_ electrolyte (0.1 M and 0.5 M), with protocols including both open-circuit potential (OCP) pretreatment and direct polarisation at potentials such as −0.975 and −1.025 V *vs.* RHE.^26^ IL-SEM imaging revealed that restructuring occurs at the onset of the reaction, characterised by the appearance of newly formed Cu nanostructures at locations distinct from the original features rather than the direct evolution of pre-existing morphologies. This behaviour was observed across all catalyst types and was more pronounced in samples subjected to OCP pretreatment, where larger, more uniformly distributed redeposited features were detected, whereas samples without OCP exhibited finer structures. The extent of restructuring depended strongly on the initial oxidation state, with oxide-derived Cu undergoing more extensive morphological transformation compared to Cu foil. These changes were associated with the dissolution of Cu species followed by their redeposition under cathodic bias, with IL-SEM indicating spatial separation between dissolution and nucleation sites. Inductively coupled plasma mass spectrometry (ICP-MS) analysis confirmed higher concentrations of dissolved Cu species in oxide-rich catalysts, while gas chromatography (GC) measurements showed that OCP-treated catalysts exhibited increased ethylene and hydrogen production, whereas catalysts polarised without OCP produced less hydrogen and ethylene but higher amounts of CO and CH_4_.

Copper foil has been investigated under CO_2_ reduction conditions in 0.1 M KHCO_3_ at −1.0 V *vs.* RHE, with periodic 2 s oxidative pulses applied every 2 min, reaching potentials above 0.6 V *vs.* RHE over 23 h.^27^ IL-SEM imaging revealed continuous restructuring of the surface during operation, with early-stage modification within the first several hours characterised by the dissolution of initial features and the emergence of spherical and grain-like structures, alongside pyramidal surface motifs whose facet assignment remained uncertain. On longer timescales, from approximately 5 to 23 h, the surface evolved further, forming extended dendritic architectures distributed across the electrode. These structural changes were accompanied by a progressive increase in ethylene selectivity and a decrease in hydrogen evolution, as determined by GC analysis. Electrolyte analysis by ICP-MS showed a substantial increase in dissolved Cu species under pulsed conditions (259 ± 61 ppb) compared to static electrolysis (6.8 ± 3.7 ppb), consistent with enhanced dissolution-redeposition during operation. Post-reaction AC-STEM of the dendritic structures revealed a polycrystalline morphology with multiple grains and the presence of high-index surface facets, indicating that the change in selectivity towards ethylene is due to the formation of active sites with undercoordinated copper atoms.

CuO_*x*_ particles can also serve as a starting point in CO_2_RR. When a negative potential is applied, CuO_*x*_ is reduced to metallic copper, which provides active sites for CO_2_ reduction. For example, CuO_*x*_ particles of a hundred nm in size electrodeposited onto a glassy carbon rotating disk electrode (RDE) have been exposed to −0.7 V *vs.* RHE in 0.1 M KHCO_3_ saturated with CO_2_.^28^ After 20 or 30 min of catalysis, IL-SEM imaging indicated that the original particles have shrunk slightly and surfaces became uneven and pitted (Fig. 4d). A closer inspection showed the formation of a set of new smaller particles of 20–40 nm. The authors explained the transformation by a two-step transformation mechanism – cathodic dissolution of CuO_*x*_ followed by electrochemical deposition of metallic copper. The redeposition process is highly complex and affected by many local and bulk parameters like local Cu ion concentration, presence of nucleation sites, effective local pH and potential.

It is interesting to compare an example *in situ* EM for Cu_2_O cubes evolution in CO_2_RR. *In situ* STEM imaging using a liquid electrochemical cell TEM holder was performed on Cu_2_O cubes with three sizes of 80, 170 and 390 nm grown electrochemically on a carbon support and subjected to −1.1 V *vs.* RHE in 0.1 M KHCO_3_.^29^*In situ* observations detected only slight changes in the size of the cubes during CO_2_RR, but their internal structure undergoes significant transformations, becoming porous. This was attributed to Cu_2_O to Cu reduction under the applied potential. Similar to the previous example, new, smaller particles ∼40 nm are formed on the original cubes and carbon film, which is explained by leaching and redeposition of Cu. This change has been shown to be beneficial for CO_2_RR, as it was linked with an increase in the efficiency towards C_2+_ products for the 170 nm cubes. However, it is important to note that because this is an *in situ* measurement, the electron beam plays an active role, for example, *via* chemical reactions induced in water (radiolysis). This is an inevitable side effect which ultimately causes the formation of gas bubbles in the liquid TEM cell, preventing measurements beyond 9 min of the experiment in this study, which is not a problem for IL-EM methodology.

Copper can be an effective catalyst for CO_2_ reduction not only in the form of nanoparticles but also in microstructure form, such as electrodeposited foam with a thickness of 30–43 micrometre and pores of 15–25 micrometre.^30^ The copper foam was heated in air first to convert it into copper oxide, which then became reduced back to metallic copper during the CO_2_ reduction. IL-SEM of the catalyst exposed to −0.67 V *vs.* RHE in 0.5 M KHCO_3_ for 1 h showed no changes in the microscale structure of the catalyst, but some subtle changes at the nanoscale, such as grain coarsening, attributed to the electrochemical reduction of CuO_*x*_ to Cu (Fig. 4e–g). The study applied *in operando* X-ray absorption spectroscopy (XAS), X-ray diffraction (XRD) and Raman, and correlated the formation of metallic copper with the production of hydrocarbons (such as ethylene, ethane) and alcohols (such as ethanol and *n*-propanol).

It has been demonstrated that the IL-SEM approach can be effectively applied to the investigation of the bulk copper surface in CO_2_RR. For example, an electrochemically roughened copper surface with features on the scale of 50 nm has been studied by IL-SEM after 60 min at −0.75 V *vs.* RHE in CO_2_-saturated 0.5 M KHCO_3_.^31^ The microscopy imaging showed no morphological changes of the copper surface on the scale of 50 nm, while the voltammetry measurements clearly indicated a drop in catalytic activity. The authors concluded that the changes in copper catalysts in CO_2_RR cannot be attributed to cathodic restructuring, which is widely used as an explanation in other studies, as a copper catalyst is much more stable in CO_2_RR than it is widely believed. The authors proposed that the catalytic activity is affected by modification of the surface layer of the catalyst *via*, for example, poisoning or restructuring on a very small scale. Importantly, the authors suggested that the changes in copper catalyst reported by previous studies are likely to be related to accidental oxidation of the copper prior to CO_2_ reduction in the experiments.

A recent investigation into carbon-wrapped Cu structures sized between 0.1–1.0 µm, with surfaces primarily composed of Cu oxides, elucidated the dynamic deactivation mechanisms during CO_2_RR using IL-SEM.^32^ The catalysts were subjected to a sequence of OCP exposures and controlled electrolysis (potentials ranging from −0.8 to −1.1 V *vs.* RHE) in CO_2_- or Ar-saturated 0.1 M KHCO_3_. IL-SEM imaging before and after 25 min OCP exposure revealed size-dependent dissolution of sub-10 nm Cu nanoparticles, while larger subsurface carbon-embedded Cu structures remained intact, indicating dissolution suppression due to physical encapsulation. Further IL-SEM analysis following an extended CO_2_-saturated electrolysis process uncovered a stochastic dissolution behaviour among micron-scale particles, highlighting the combined influence of morphological variations and local microenvironment effects. Notably, avoiding OCP exposure effectively suppresses Cu dissolution and mitigates catalyst restructuring. Additionally, IL-SEM revealed a dynamic interplay between electrodeposition and CO_2_RR: nanoparticles formed at −0.975 V *vs.* RHE (optimal for C–C coupling) exhibited rough surfaces, compared to those formed at less reducing potentials. These structural/morphological transformations directly correlated with progressive selectivity shifts from CO_2_ reduction products toward hydrogen evolution. This transition was attributed to four key factors: (1) nanoparticle size distribution, (2) crystallographic facet orientation, (3) CO intermediate surface coverage, and (4) the ratio of bridge-bound to atop-bound CO species, collectively governing catalyst performance and deactivation pathways.

A recent work extended identical-location analysis of Cu catalysts in CO_2_RR to the atomic scale by combining IL-TEM and IL-AC-STEM with a graphitised carbon nanofiber (GNF) support.^21^ Cu atoms were deposited directly onto GNFs by magnetron sputtering, producing ligand-free Cu nanoparticles, nanoclusters and isolated atoms on a conductive, nanotextured carbon support. By tracking the same Cu/GNF regions before and after OCP exposure and short CO_2_RR treatment, the study showed that the OCP stage can strongly precondition the catalyst by promoting Cu leaching and dissolution–redeposition processes. When the OCP step was eliminated, solution-mediated Cu loss was largely suppressed, allowing surface-confined restructuring pathways intrinsic to CO_2_RR to be examined. IL-TEM and IL-AC-STEM revealed that several single-particle events can coexist under the same macroscopic reaction conditions, including Ostwald ripening, coalescence, selective etching, disappearance of small particles, formation of new 1–3 nm particles, emergence of single Cu atoms, and rare Coulomb-explosion-like fragmentation of larger nanoparticles into smaller particles confined to their original footprints. These observations demonstrate that Cu catalyst dynamics in CO_2_RR are not governed by a single universal restructuring pathway, but are highly sensitive to the local nanoscale environment, including support nanotexture, defect density, anchoring strength and electronic coupling with the carbon support. This work, therefore, highlights the unique value of IL-EM for resolving particle-to-particle heterogeneity and identifying competing catalyst-evolution pathways that would be hidden in ensemble-averaged measurements.

Among the metals, silver is known as an efficient catalyst for the conversion of CO_2_ to CO, and therefore, it attracted attention to IL-EM. For example, 7 nm Ag nanoparticles modified with a lipid ligand and deposited on a TEM grid on an RDE demonstrated excellent stability during 4 h of operation at −1 V *vs.* RHE in CO_2_-saturated 0.1 M KHCO_3_, with IL-TEM indicating no significant changes in the nanoparticles (Fig. 4h–l).^33^ In contrast, the Ag black sample containing nanoparticles of various sizes undergoes extensive particle migration and coalescence under the same conditions.

In contrast, spherical Ag nanoparticles with 5–10 nm diameter on carbon black exposed to −0.82 V *vs.* RHE in CO_2_-saturated 0.5 M KHCO_3_ after 1 h show significant changes as well as growth of new Ag nanoparticles (Fig. 4j–o), which continued growing up to 6 h.^34^ The changes are correlated with the decrease in current density on the RDE in the first 3 h. A loss of electrochemically active surface area (ECSA) and an increase in parasitic HER in the bulk measurements have been observed.

Silver cubes of 110 nm supported on carbon black were integrated into a gas diffusion electrode (GDE) and subjected to electrochemical CO_2_ reduction under varying conditions (*e.g.*, 2 M KOH at −1.84 V *vs.* Ag/AgCl for 30–60 min or −2.07 V *vs.* Ag/AgCl for 13–32 min) with CO_2_ introduced as gas through the GDE.^35^ IL-SEM revealed no significant morphological changes or catalyst detachment (Fig. 5a), indicating that the main mechanism of deactivation is carbonate deposition at high pH. This is also correlated with the observation that at more cathodic potentials, CO production was initially more prominent but declined over time.

**Fig. 5:**
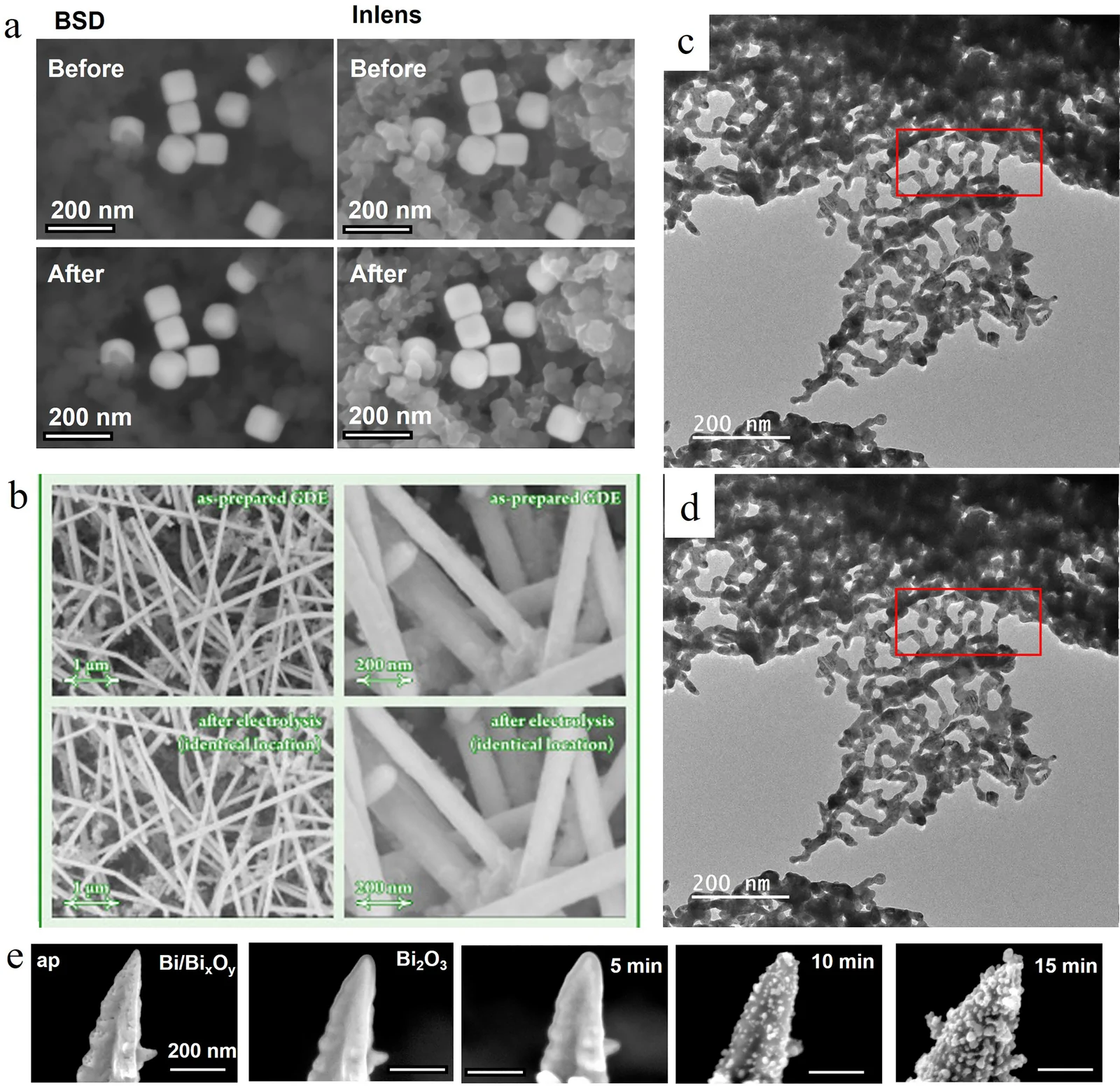
(a) IL-SEM inspection of Ag-GDE cathode surfaces before and after gas-fed CO_2_RR experiments at −1.84 V for 30 min. Adapted from ref. 35 with a figure provided by Pavel Moreno García. (b) IL-SEM inspection of as-prepared GDEs and the same GDEs after electrolysis performed with the charge consumption of 1839 C cm^−2^. Reproduced from ref. 36 with permission from John Wiley and Sons, copyright 2022. IL-TEM inspection of Au aerogel before (c) and after (d) CO_2_RR at −0.5 V *vs.* RHE in CO_2_-saturated 0.5 M KHCO_3_. Reproduced from ref. 37 with permission from the American Chemical Society, copyright 2022. (e) IL-SEM images of morphological changes associated with the thermal annealing of the Bi/Bi_*x*_O_*y*_ foam and subsequent potentiostatic CO_2_ electrolysis in the CO_2_-saturated 0.5 M KHCO_3_ solution at −0.6 V *vs.* RHE. Adapted from ref. 38 with a figure provided by Abhijit Dutta.

Silver nanowires on carbon black have been applied for a GDE operated in a galvanostatic regime and converting humidified CO_2_ to CO.^36^ IL-SEM showed no significant changes in the catalyst under these conditions (Fig. 5b).

Gold is known as a good catalyst for CO_2_RR to CO, and it was investigated in the form of an aerogel consisting of ∼5 nm strings of metal prepared by chemical reduction of a gold salt, and compared with commercial 9 nm Au nanoparticles on carbon black.^37^ Catalysts were deposited on an Au finder grid and attached to an RDE. After 1 h at −0.5 V *vs.* RHE in CO_2_-saturated 0.5 M KHCO_3_ RDE, IL-TEM imaging indicated no significant changes in the aerogel catalyst (Fig. 5c and d), but 9 nm Au nanoparticles on carbon black migrated on the support and coalesced, with the carbon black support also exhibiting movement.

A main group element, bismuth, in the form of Bi/Bi_*x*_O_*y*_ foam with pores of 15–25 microns and a few microns long dendritic structures, electrodeposited and annealed in air, has demonstrated remarkably high faradaic efficiency for formate at a relatively low overpotential −0.6 V *vs.* RHE.^38^ The authors applied IL-SEM to evaluate the catalyst before and after 15, 30 and 45 min treatment in CO_2_-saturated 0.5 M KHCO_3_ solution at −0.6 V *vs.* RHE (Fig. 5e). IL-SEM showed no changes in the microstructure of the catalyst but detected sub-10 nm particles growing on the dendrites. Long-term catalyst stability measurements up to 100 h indicated that most changes in the catalyst happen in the first 1 h of CO_2_RR. The EM was correlated with increases in ECSA, and *in situ* Raman spectroscopy that detected bismuth sub-carbonate (BiO)_2_CO_3_ formation on the catalyst surface, which plays an important role in the selective production of formate.

#### 3.1.2. Bimetallic catalysts

Gold and copper have been effectively combined in different ratios of AuCu and Au_3_Cu aerogels consisting of 7 nm strings of alloys for CO_2_RR to CO.^39^ The authors applied IL-TEM (Fig. 6a and b) and IL-EDS to evaluate transformations in the catalyst, both during the stage of conditions/activation and CO_2_RR. During the conditioning stage, when the catalyst is cycled between 0.1 and 1.7 V *vs.* RHE, the overall structure of the aerogel remains largely intact, but Cu-rich regions on the sides of the alloy particles become etched away, which is confirmed with lead underpotential deposition (Pb-UPD), indicating the removal of the Cu phase from the surface of the catalyst. Next, the catalyst was subjected to CO_2_RR conditions at −0.5 V *vs.* RHE in CO_2_-saturated 0.5 M KHCO_3_ on a Ti TEM grid. After 75 min, IL-TEM imaging and IL-EDS spectroscopy showed no changes in the structure or composition of the bimetallic aerogel, which is consistent with stable current density and ECSA.

**Fig. 6:**
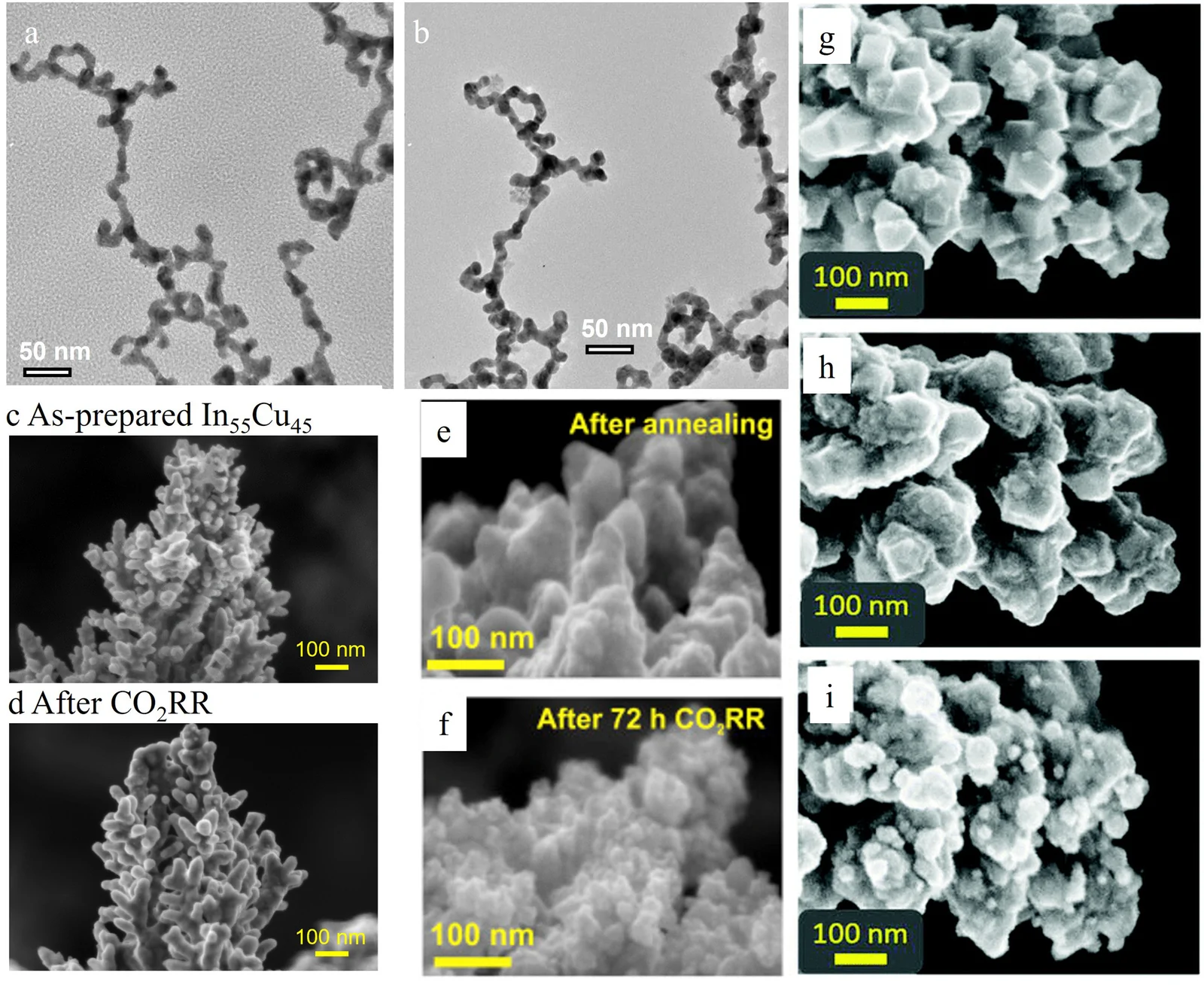
IL-TEM inspection acquired on the Au_3_Cu aerogels before (a) and after (b) 5 cycles of cyclic voltammetry (CV)-treatment in CO_2_-saturated 0.5 M KHCO_3_. Adapted from ref. 39 with a figure provided by Juan Herranz and Piyush Chauhan. IL-SEM images of the In_55_Cu_45_@Cu alloy catalyst (c) before and (d) after the electrolysis (30 h at −1.0 V *vs.* RHE in the CO_2_-saturated. 0.5 M KHCO_3_ electrolyte). Adapted from ref. 40 with a figure provided by Motiar Rahaman. IL-SEM images of the Ag_15_Cu_85_ micro-foam before (e) and after (f) 72 h CO_2_RR at −1.0 V *vs.* RHE. Reproduced from ref. 41 with permission from Elsevier, copyright 2020. IL-SEM inspection of the Pd_9_Cu_91_ alloy foam: (g) as-prepared alloy foam; (h) thermally annealed alloy foam (200 °C, 12 h, in air); (i) oxide-derived alloy foam (after electrochemical pretreatment at −0.65 V *vs.* RHE for 45 min). Reproduced from ref. 42 with permission from the Royal Society of Chemistry, copyright 2020.

In_55_Cu_45_ alloy foam with a thickness of 20 microns and dendritic structures on the order of 100 nm was prepared by electrodeposition and applied as a highly selective catalyst for CO_2_ to formate conversion.^40^ After 30 h at −1.0 V *vs.* RHE in the CO_2_-saturated 0.5 M KHCO_3_, IL-SEM imaging did not indicate any changes in the foam structures but only subtle changes in the morphology of the dendrites at the scale of *ca.* 100 nm (Fig. 6c and d). This supports the high stability and activity of the catalyst measured in electrochemistry.

A similar bimetallic Ag_15_Cu_85_ micro-foam, where Ag metal forms junctions with CuO and Cu_2_O, has been investigated by IL-SEM as a catalyst for ethanol production (Fig. 6e and f).^41^ At −1 V *vs.* RHE in CO_2_-saturated 0.5 M KHCO_3_, where ethanol is formed with 33% FE, the catalyst exhibited no changes in the microstructure, but roughening of the surface morphology at the nanoscale was attributed to the reduction of copper oxides to metallic copper during CO_2_RR. This ensures that the overall current density and FE for ethanol remain stable in this catalyst over 100 h.

A Pd_9_Cu_91_ alloy foam with 23-micron pores and 25-micron thickness was grown by electrochemical deposition followed by annealing in air.^42^ The catalyst was studied by IL-SEM imaging before and after CO_2_RR in 0.5 M KHCO_3_ for 45 min at −0.65 V *vs.* RHE. While no significant microscale changes were detected, IL-SEM revealed the growth of new 10–50 nm nanoparticles (Fig. 6g–i). These changes were linked to the increase in ECSA during CO_2_RR and *in situ* XRD measurements, confirming the reduction of oxide phases to the metallic phase. This allowed the authors to establish a complete picture of the catalyst evolution and its implications for selectivity. Changes in the catalyst detected by IL-SEM were assigned to phase segregation of the catalyst into nm-sized Pd-rich and Cu-rich domains that boost selectivity for PrOH. A proposed mechanism involves a concerted spillover of chemisorbed carbon monoxide (*CO) and atomic hydrogen (*H) species from the Pd-rich domains to the activated Cu-rich domains of the catalyst under CO_2_RR conditions.

IL-EM has proven to be an effective methodology for studying the evolution of various electrocatalysts in the CO_2_RR process. This includes a range of systems from single metals to bimetallic alloys and heterogeneous materials. Notably, bi-intermetallic and multi-element materials, such as high-entropy alloys, deserve further investigation. The studies conducted have demonstrated that researchers can revisit points of interest both before and after the reaction occurs. While most studies focus on changes at the micro- and tens-of-nanometer scales, there is a pressing need to explore the nm and sub-nm scales. This is crucial because electrocatalysis predominantly occurs at nanoscale surface features, making high-resolution essential. Even though IL-SEM or IL-TEM may show no observable changes at low magnifications in many cases, this does not imply that the catalyst surface remains unaltered. To gain deeper insights from IL-EM imaging, it is important to incorporate advanced microscopy techniques, including but not limited to 3D tomography and 4D STEM.

### 3.2 Oxygen reduction reaction (ORR)

#### 3.2.1 Single-metal catalysts

##### Pt-based catalysts

Carbon-supported Pt nanoparticles are widely used as cathode catalysts in proton exchange membrane fuel cells, yet their durability remains limited under repeated potential cycling. To elucidate the structural origins of this instability, the degradation of Pt nanoparticles supported on Vulcan carbon was investigated using IL-TEM during a load-cycle accelerated stress test (AST) comprising 20 000 potential cycles between 0.6 and 0.95 V *versus* RHE (Fig. 7a–c).^43^ Tracking the same catalyst regions before and after electrochemical treatment showed that the carbon support largely preserves its morphology within this potential window, whereas the Pt nanoparticles undergo pronounced structural evolution. Particle-resolved analysis reveals multiple degradation pathways, including particle disappearance, size change, migration, and coalescence, which collectively reflect processes associated with Pt dissolution and subsequent redistribution. Statistical analysis of hundreds of individual nanoparticles further enabled quantification of the relative frequencies of these transformations. Complementary IL-STEM provided direct evidence that dissolved Pt species can reappear on the carbon support as isolated single atoms. Electrochemical measurements using rotating ring-disk electrode techniques indicate that the emergence of these atomic Pt species alters the oxygen reduction pathway, increasing the formation of H_2_O_2_ relative to the four-electron reduction route. Consistent with these structural changes, the bulk catalyst exhibited a substantial decline in electrochemical performance after the stress test, with the ECSA decreasing from 85.8 to 43.8 m^2^ g^−1^ and the half-wave potential shifting negatively by 48 mV. Together, these observations highlight how nanoparticle dissolution and the generation of dispersed Pt atomic species contribute to both structural and functional degradation of Pt/C catalysts.

**Fig. 7:**
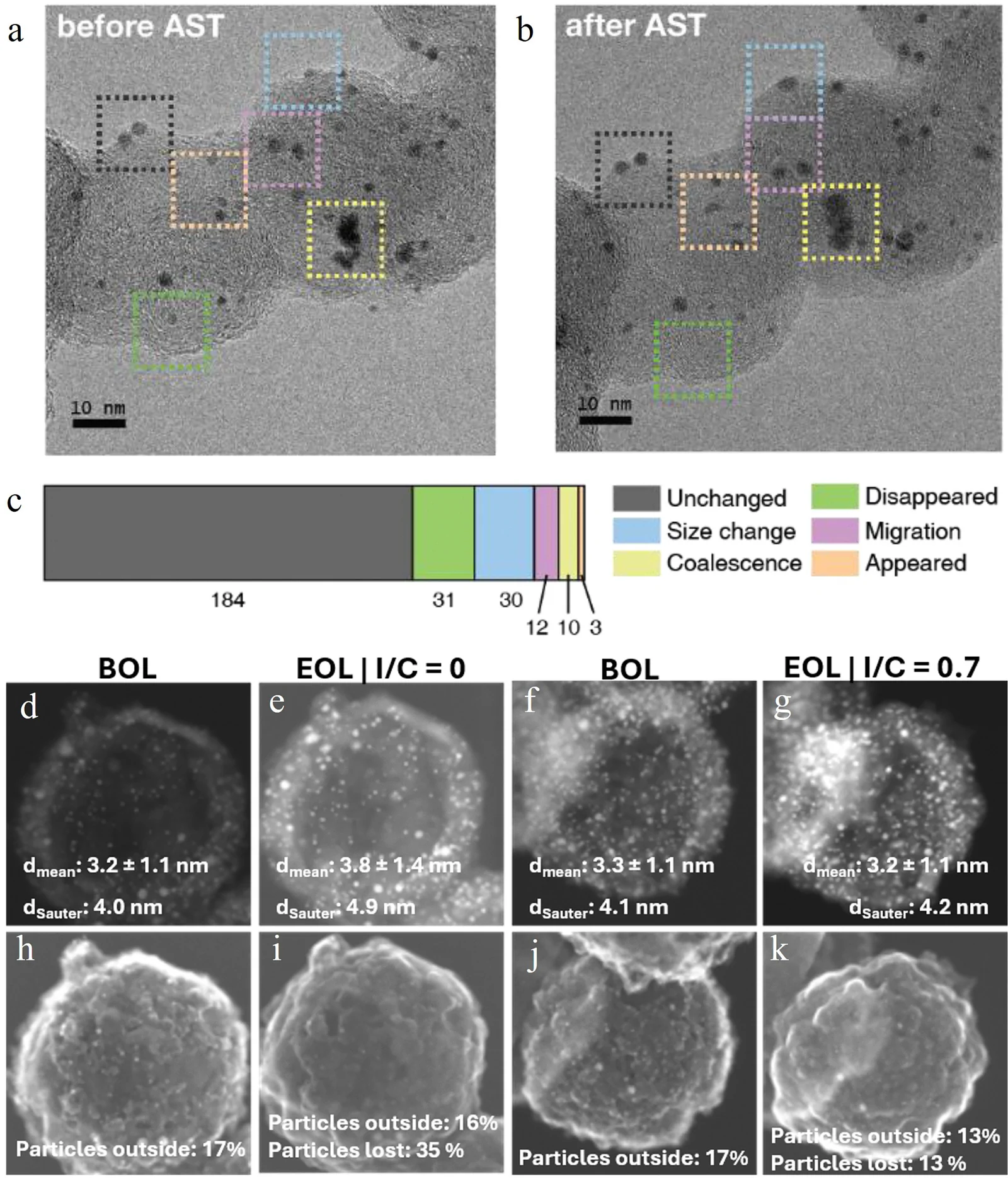
IL-TEM images of Pt/C before (a) and after (b) AST, (c) frequencies of each categorised process. Reproduced from ref. 43 with permission from John Wiley and Sons, copyright 2023. IL-high-angle annular dark-field scanning transmission electron microscopy (HAADF-STEM) (d)–(g) and IL-SE-STEM (h)–(k) images of Pt/HGS at the beginning of life (BoL) and end of life (EoL), without ionomer (I/C = 0) and with ionomer (I/C = 0.7), where I/C denotes the ionomer-to-carbon mass ratio, after ageing at 80 °C in 0.1 M HClO_4_. Adapted from ref. 44 with a figure provided by Marc Ledendecker.

Pt/C catalysts supported on carbons with different pore structures were investigated to examine the effects of ionomer-induced degradation, including nonporous Pt/Vulcan (2.7 nm), microporous Pt/Ketjenblack (2.4 nm), and mesoporous Pt/hollow graphitic spheres (Pt/HGS, 3.1 nm).^44^ The catalysts were subjected to a voltage-cycling AST consisting of 20 000 square-wave cycles between 0.6 and 1.0 V *vs.* RHE at 80 °C in 0.1 M HClO_4_ using a rotating disk electrode configuration. Structural evolution of the nanoparticles was analysed using IL-STEM in combination with secondary electron (SE) imaging, including IL-STEM/SE-STEM analysis (Fig. 7d–k). These measurements enabled differentiation between particles located on the external carbon surface and those confined within the pore network. The results reveal a strong dependence of degradation behaviour on nanoparticle location. For particles located outside the pores, the presence of ionomer enhanced dissolution, driven by the adsorption of acidic sulfonate groups onto accessible Pt surfaces. In contrast, nanoparticles located within pores exhibited a lower probability of dissolution and reduced particle growth. This behaviour was attributed to the limited accessibility of sulfonate groups and to pore confinement influencing nanoparticle stability. Consequently, porous carbon supports such as Ketjenblack and hollow graphitic spheres displayed different restructuring behaviour compared with the nonporous Vulcan support, highlighting the importance of carbon-support porosity in determining ionomer–catalyst interactions during catalyst degradation.

Pt nanoparticles supported on titanium oxynitride (Pt-TiO_*x*_N_*y*_/C) with an average particle size of 2.9 nm were investigated by IL-four-dimensional scanning transmission electron microscopy (4D-STEM) (Fig. 8a and b) before and after electrochemical perturbation cycling.^45^ The electrochemical treatment consisted of potentiodynamic cycling between 0.05 and 1.2 V *vs.* RHE for 200 cycles at a scan rate of 300 mV s^−1^ in 0.1 M HClO_4_ under an argon atmosphere. Structural characterisation using IL-STEM, including ADF imaging and 4D-STEM acquisition, together with EELS mapping of Ti, revealed that the initially observed gap between Pt nanoparticles and the TiO_*x*_N_*y*_ support disappeared after electrochemical perturbation. The resulting configuration showed Pt nanoparticles located in a pocket-like junction with the TiO_*x*_N_*y*_ support. Additional electrochemical measurements indicated that the Pt-TiO_*x*_N_*y*_/C catalyst exhibits a smaller Tafel slope than the Pt/C reference catalyst.

**Fig. 8:**
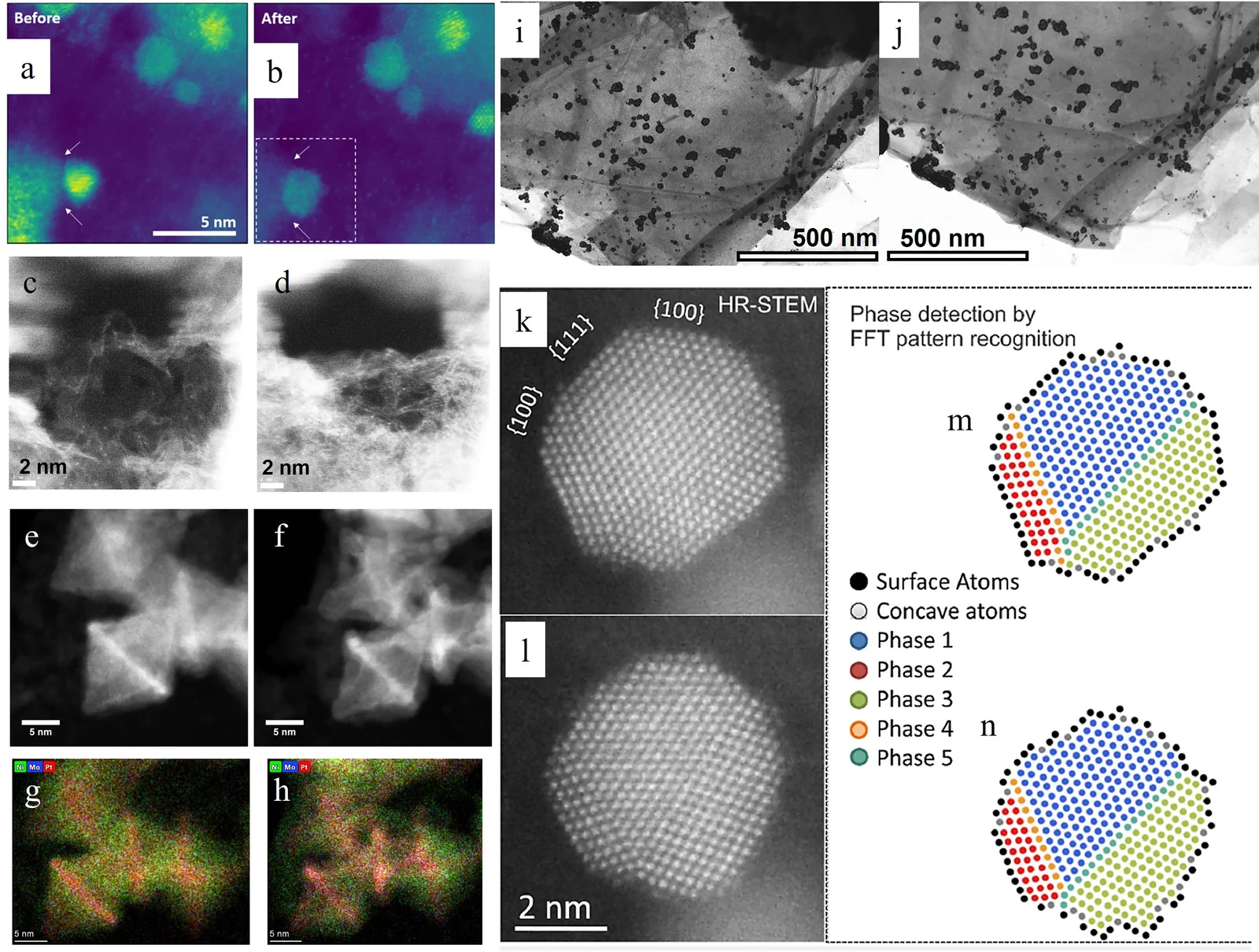
IL-4D-STEM images of Pt-TiO_*x*_N_*y*_/C before (a) and after (b) electrochemical perturbation. Reproduced from ref. 45 with permission from the American Chemical Society, copyright 2024. IL-HAADF-STEM images of Fe–N–C before (c) and after (d) AST. Adapted from ref. 46 with a figure provided by Laetitia Dubau. IL-HAADF images of (e) before, (f) after AST and IL-STEM-EDS of (g) before, (h) after AST for PtNi(Mo). Adapted from ref. 47 with a figure provided by Marc Heggen. IL-TEM of HG13 before (i) and after AST (j). Adapted from ref. 48 with a figure provided by Christopher Tremblay. IL-AC-STEM images of Pt–Co nanoparticle (k) before and (l) after electrochemical activation, and corresponding atomic columns with phase detection before (m) and after (n). Reproduced from ref. 49 with permission from Elsevier, copyright 2021.

##### Non-Pt single metal

Atomically dispersed Fe–N–C catalysts were examined by HAADF-STEM imaging in identical-location mode before and after AST (Fig. 8c and d).^46^ The catalyst was subjected to 10 000 load-cycle potential steps between 0.6 and 1.0 V *vs.* RHE with a holding time of 3 s at each potential in Ar-saturated 0.1 M H_2_SO_4_ at 60 °C. Identical-location HAADF-STEM observations revealed clogging of mesopores and densification of the N-doped carbon matrix after electrochemical cycling. Despite these structural changes in the carbon support, no clustering of Fe atoms was observed, indicating that Fe species remained atomically dispersed after the load-cycle protocol. Electrochemical measurements showed a decrease in mass activity after the accelerated stress test.

##### Bimetallic catalysts

IL-STEM combined with EDS was used to examine the structural and compositional evolution of carbon-supported octahedral PtNi and Mo/Rh-doped PtNi nanoparticles (6.97 ± 1.61 nm and 9.97 ± 2.27 nm), with and without prior reductive heat treatment, subjected to AST (10 800 cycles, 0.6–0.95 V *vs.* RHE in Ar-saturated 0.1 M HClO_4_).^50^ At the single-particle level, the undoped PtNi catalyst exhibited pronounced Ni depletion (32.4% ± 4.3%), accompanied by preferential dissolution of Ni-rich facets and transformation of the octahedral structure into a Pt-enriched framework. In contrast, both heat-treated PtNi and Mo/Rh-doped systems showed substantially reduced Ni loss (6.1% ± 3.4% and ∼8% ± 8%, respectively), while largely retaining their original particle morphology. Across all samples, IL-STEM analysis revealed no evidence of particle displacement or detachment during cycling, indicating that degradation proceeds predominantly through compositional modification rather than particle mobility. These observations are consistent with electrochemical measurements, in which the undoped PtNi catalyst exhibited a measurable decrease in ECSA after cycling, whereas doped and heat-treated catalysts showed minimal variation in ECSA, reflecting improved stability under ORR-relevant conditions.

Mo-doped PtNi octahedral nanoparticles with an average edge length of 12.4 nm were investigated before and after AST (10 800 cycles, 0.6–0.95 V *vs.* RHE in Ar-saturated 0.1 M HClO_4_).^47^ IL-STEM (Fig. 8e and f) enabled direct comparison of the same nanoparticle before and after electrochemical treatment, revealing a clear transformation of the octahedral morphology, with initially flat facets developing concave features. Elemental analysis by STEM-EDS (Fig. 8g and h) demonstrated a substantial change in the Pt : Ni ratio, from 0.25 : 1 before the test to 1.5 : 1 after AST, providing direct evidence for Ni leaching during electrochemical operation. Consistent with this compositional change, post-AST characterisation indicated that the catalysts evolved toward a Pt-enriched structure. The combined structural and compositional observations therefore indicate that electrochemical testing induces Ni dissolution and facet restructuring in the PtNi(Mo) nano-octahedra, thereby forming Pt-rich surfaces.

Hollow porous PtNi nanoparticles were synthesised on graphene nanoplatelets using a Pt : Ni precursor ratio of 1 : 3.^48^ Catalysts supported on hydrogenated graphene nanoplatelets (HG13) and pristine graphene nanoplatelets (G13) exhibited average particle diameters of 21.13 ± 4.18 nm and 25.87 ± 6.38 nm, respectively. Durability was evaluated using AST, comprising 6000 potential cycles between 1.0 and 1.6 V *vs.* RHE in 0.1 M KOH at 25 °C. IL-TEM (Fig. 8i and j) revealed substantial structural degradation of the G13 catalyst after cycling, where pronounced carbon corrosion of the graphene support was accompanied by nanoparticle aggregation. In contrast, the HG13 composite exhibited minimal observable carbon corrosion and largely preserved nanoparticle dispersion. These structural observations are consistent with electrochemical measurements, in which the HG13 catalyst retained 61.9% of its ECSA and 53.0% of its activity after the AST, compared with 36.8% and 26.6% for the G13 analogue. The improved durability of the hydrogenated composite was attributed to the hydrophobic character of the hydrogenated graphene support, which reduces water interactions and mitigates carbon corrosion.

Electrochemical activation of Pt–Co/C nanoparticles was investigated using IL-EM combined with a modified floating-electrode configuration.^49^ The catalyst-coated TEM grid was subjected to a potential dynamic activation protocol consisting of 200 cycles between 0.05 and 1.2 V *vs.* RHE at a scan rate of 300 mV s^−1^ under a nitrogen atmosphere in 0.1 M HClO_4_. IL-SEM analysis of the catalyst film before and after activation revealed no significant changes in the overall morphology of the catalyst layer, indicating that the electrochemical treatment did not induce large-scale restructuring of the film. In contrast, IL-STEM observations at higher spatial resolution (Fig. 8k–n) revealed multiple structural transformations of individual Pt–Co nanoparticles, including necking between adjacent particles, separation of previously connected particles (anti-necking), and particle shrinkage associated with dissolution processes. Atomically resolved STEM analysis further revealed etching and redeposition of atomic columns on nanoparticle surfaces, leading to reshaping and a progressive rounding of particle morphology. Quantitative EDS analysis showed compositional evolution during activation, with the Pt/Co ratio increasing from approximately 2 to 3.5, consistent with preferential Co dissolution and the formation of Pt-enriched surface regions.

Cu core Pt shell nanoparticles supported on nitrogen-doped carbon (PtCu/C_N_) with an average size of approximately 4 nm were investigated together with a commercial Pt/C catalyst.^51^ Electrochemical activation was performed by cycling the potential between 0.04 and 1.0 V or 0.04 and 1.2 V for 100 cycles in 0.1 M HClO_4_ at a scan rate of 200 mV s^−1^. IL-TEM analysis (Fig. 9a–d) showed that the average particle size decreased with increasing upper activation potential, consistent with dissolution occurring during the electrochemical treatment. Structural evolution of individual nanoparticles included particle disappearance, migration across the carbon support, and aggregation into larger particles. EM observations further indicated that both platinum and copper dissolve during activation, with copper dissolving more rapidly, thereby increasing the relative platinum content and thickening the platinum shell in core–shell nanoparticles. IL-EDS mapping confirmed a decrease in the copper fraction after activation. Electrochemical measurements showed that the ECSA was largely independent of the activation protocol, whereas the oxygen reduction activity depended strongly on the upper activation potential. In particular, the PtCu/C_N_ catalyst activated to 1.0 V exhibited a mass activity 1.7 times higher than that obtained after activation to 1.2 V, and significantly higher than that of the commercial Pt/C catalyst.

**Fig. 9:**
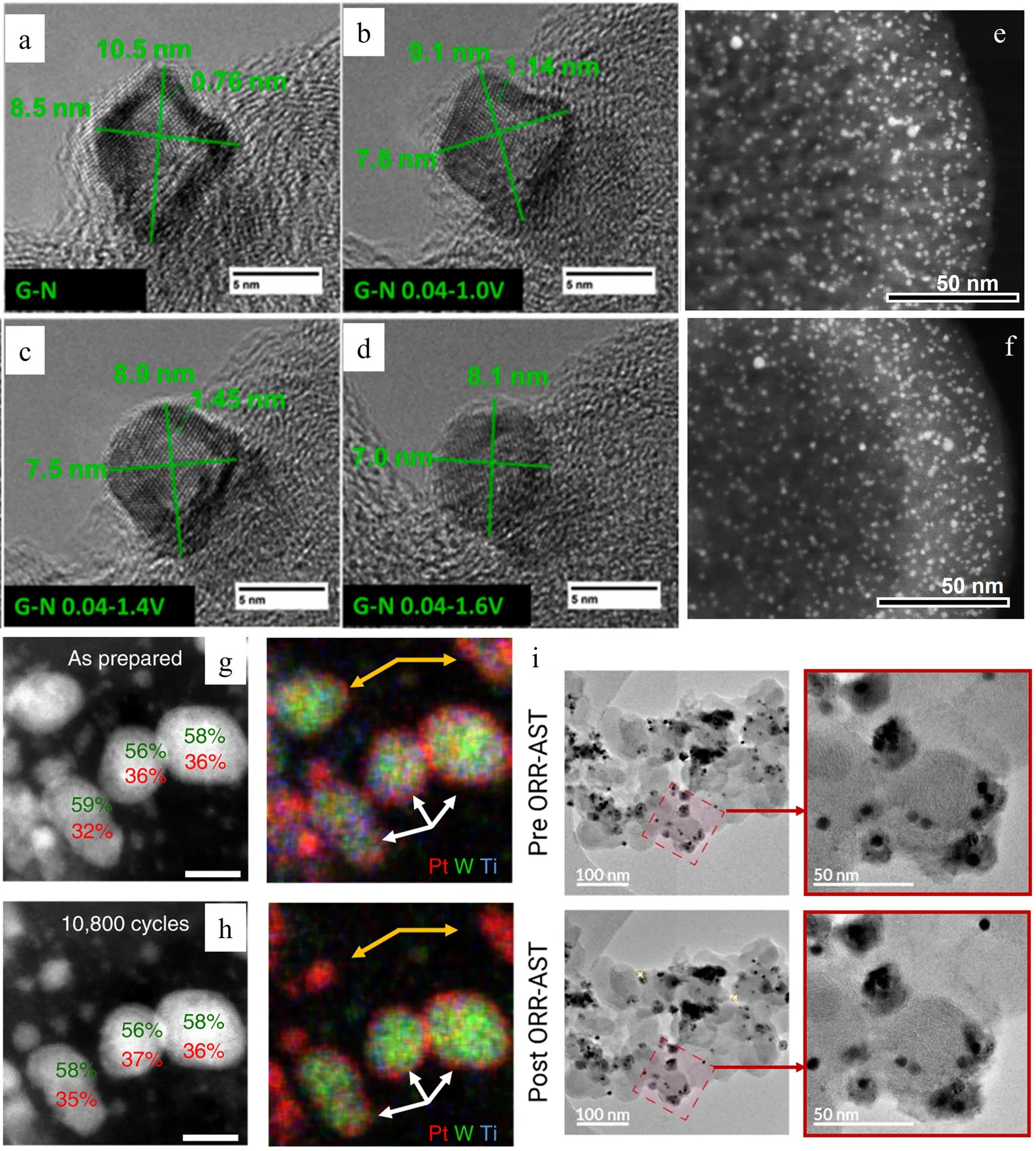
(a)–(d) IL-high-resolution transmission electron microscopy (HRTEM) of PtCu/C_N_ under different activation potentials. Reproduced from ref. 51 with permission from Elsevier, copyright 2024. IL-STEM images of O-PtCu/HMCS (e) before, (f) after ADT. Adapted from ref. 52 with a figure provided by Zhiping Deng and Mingxing Gong. IL-STEM-EDS of 2 ML Pt/TiWC before (g) and after (h) ASTs. Reproduced from ref. 54 with permission from Springer Nature, copyright 2020. (i) IL-TEM images of core–shell PtNi before and after ORR-AST, respectively. Adapted from ref. 55 with a figure provided by Marian Chatenet and David Zitoun.

Three PtCu catalysts were investigated, including disordered PtCu alloy nanoparticles supported on carbon (D-PtCu/C), ordered PtCu intermetallic nanoparticles on carbon (O-PtCu/C), and ordered PtCu intermetallic nanoparticles supported on hollow mesoporous carbon spheres (O-PtCu/HMCS).^52^ TEM showed that D-PtCu/C and O-PtCu/HMCS consisted of nanoparticles with an average size of approximately 3 nm. The electrochemical durability of the catalysts was evaluated using an accelerated durability test with potential cycling between 0.6 and 1.0 V in 0.1 M HClO_4_. IL-TEM analysis revealed substantial structural degradation in D-PtCu/C during cycling, including particle dissolution and aggregation, which is attributed to the unstable nature of Cu in the disordered alloy structure. The dissolved Cu^2+^ species in the electrolyte were also associated with corrosion of the carbon support. In contrast, the majority of PtCu particles in O-PtCu/C were preserved after cycling, consistent with the stabilisation of Cu in the ordered intermetallic structure. However, deformation at the boundaries of the carbon support was observed, which induced particle migration, detachment, and agglomeration. For the O-PtCu/HMCS catalyst, IL-STEM images (Fig. 9e and f) collected before and after cycling showed no significant changes in particle morphology, number, distribution, or size. Instead of aggregation, particles exhibited a tendency to disperse after cycling, which was attributed to coulombic repulsion arising from the adsorption of electrolyte ions on the HMCS surface. Electrochemical measurements further showed negligible differences in cyclic voltammetry (CV) after 50 000 cycles, while the half-wave potential decreased by 14 mV and the mass activity after cycling remained 2.15 A cm_Pt_^−2^.

IL-TEM was employed to examine the structural evolution of Pt/C, Pt/C + Ir/C, and Pt_85_Ir_15_/C catalysts during potential cycling between 1.0 and 1.5 V *vs.* RHE in O_2_-saturated 0.1 M HClO_4_ (500 mV s^−1^, 5000 cycles).^53^ Commercial Pt/C and Ir/C exhibit average particle sizes of 2.7 and 3.5 nm, respectively, while Pt_85_Ir_15_ nanoparticles are distributed with sizes of 3.6–3.9 nm. IL-TEM observations show that Pt/C undergoes substantial structural degradation, including reduction in nanoparticle number, particle detachment from the carbon support, and deformation of carbon boundaries, consistent with carbon corrosion. In contrast, Pt_85_Ir_15_/C exhibits significantly smaller losses of nanoparticles and limited deformation of the carbon support. For Pt/C + Ir/C, degradation depends on the local composition, with Pt-rich regions showing nanoparticle loss and carbon corrosion, while Ir-rich regions exhibit minimal changes. Most Pt, Ir, and Pt_85_Ir_15_ nanoparticles remain dispersed without severe agglomeration after cycling, indicating that the decrease in ECSA is mainly associated with nanoparticle loss rather than particle coarsening. Electrochemical measurements show that the ORR mass activity of Pt/C decreases from 0.12 to 0.017 A mg_PGM_^−1^ after 5000 cycles, whereas Pt/C + Ir/C decreases from 0.045 to 0.039 A mg_M_^−1^ and Pt_85_Ir_15_/C increases from 0.029 to 0.071 A mg_M_^−1^. High-resolution transmission electron microscopy (HRTEM) reveals the formation of high-index facets on Pt_85_Ir_15_/C after cycling. XPS analysis shows an increase in oxidised carbon species for all catalysts, with a larger increase for Pt/C than for Pt_85_Ir_15_/C, indicating suppressed carbon oxidation in the alloy catalyst. Differential electrochemical mass spectrometry measurements further show that CO_2_ evolution follows the order Pt/C > Pt/C + Ir/C > Pt_85_Ir_15_/C, confirming reduced carbon corrosion upon incorporation of Ir. OER measurements indicated that Pt_85_Ir_15_/C maintains a higher current density (2.70 mA cm^−2^) than Pt/C + Ir/C (1.59 mA cm^−2^) after 5000 cycles. In single-cell tests, Pt_85_Ir_15_/C exhibited negligible decay in current density compared with Pt/C and Pt/C + Ir/C. These results are attributed to the atomically dispersed Ir in Pt_85_Ir_15_/C, which suppresses carbon corrosion and improves catalyst durability.

Approximately 30 nm Pd@Pt core–shell nanoparticles, consisting of Pd nanocube cores coated with an epitaxial Pt shell of ∼5 atomic layers, were investigated with and without interstitial C/N doping to elucidate stability under potential cycling between 0.05 and 1.50 V *vs.* RHE in 0.1 M HClO_4_.^56^ At the single-particle level, the undoped system exhibited pronounced structural reconstruction, where initially well-defined core–shell particles evolved into irregular aggregates, accompanied by a decrease in Pd content from 82.2% to 74.9% and a corresponding increase in Pt fraction, as confirmed by IL-STEM and IL-EDS analysis. This evolution was attributed to preferential Pd dissolution at interfacial pinhole sites, which disrupted local coordination and led to the irreversible transformation of the core–shell structure, followed by the formation of Pt–Pd alloy particles *via* dissolution and redeposition processes. In contrast, C/N-doped Pd@Pt displayed only minor compositional and morphological variations after cycling. Real-time dissolution analysis using scanning flow cell ICP-MS showed a strong suppression of metal loss, with reductions of Pt and Pd dissolution by factors of 48.5 and 13.9, respectively. Consequently, *operando* XAS indicated that the Pt coordination environment remained comparatively stable in the doped system under oxidising potentials. These observations directly link interfacial C/N incorporation to the suppression of pinhole-initiated dissolution and to the stabilisation of the core–shell architecture.

Carbon-supported Pd/C and PdFe/C catalysts with initial particle sizes of ∼2 nm were synthesised *via* impregnation–reduction, while thermal annealing at 600 °C produced ordered PdFe intermetallic nanoparticles with enlarged sizes of ∼8 nm.^57^ IL-TEM conducted under potential cycling (0.6–1.0 V *vs.* RHE, 0.1 M HClO_4_, 100 mV s^−1^) reveals that disordered PdFe/C undergoes limited structural variation after 10 000 cycles but exhibits pronounced degradation after 30 000 cycles, including particle disappearance, coalescence, migration, and aggregation, accompanied by carbon support corrosion. Elemental mapping and statistical analysis show substantial Fe depletion, with the Pd/Fe ratio shifting from ∼1 : 1 to 87.2 : 12.8, consistent with inductively coupled plasma optical emission spectroscopy (ICP-OES) results indicating higher Fe dissolution (0.811 mg L^−1^). In contrast, Pd/C displays particle dissolution and aggregation without carbon corrosion, confirming that support degradation in PdFe/C is associated with Fe leaching. The ordered PdFe bimetallic catalyst exhibits suppressed particle migration and agglomeration, reduced Fe dissolution (0.320 mg L^−1^), and minimal carbon corrosion, while preserving the bimetallic structure after cycling. These observations established Fe leaching as the primary origin of structural degradation, whereas atomic ordering suppresses Fe loss and maintains catalyst stability, consistent with the smaller half-wave potential shift of 131 mV after 30 000 cycles.

Pt shells deposited on TiWC nanoparticle cores (Pt/TiWC) with nominal coverages of 0.7 and 2 monolayers (ML) exhibit particle sizes of 5.1 ± 1.3 and 6.6 ± 1.8 nm, respectively.^54^ IL-STEM combined with EDS analysis was used to track the structural and compositional evolution of individual particles after an AST consisting of 10 800 potential cycles between 0.4 and 1.0 V *vs.* RHE in Ar-saturated 0.1 M HClO_4_ at a scan rate of 1 V s^−1^. Particles with complete Pt shell coverage maintained their core–shell structure and elemental composition after cycling, with no evidence of alloy formation or agglomeration (Fig. 9g and h). In contrast, particles with incomplete shell coverage exhibited dissolution of the TiWC core and collapse, forming ∼4–5 nm Pt nanoparticles. Scanning flow cell with inductively coupled plasma-mass spectrometry (SFC-ICP-MS) measurements showed that increasing Pt shell thickness substantially suppresses dissolution of the core elements, with ∼80% W dissolution observed for sub-monolayer coverage compared to 17% for 2 ML Pt/TiWC. Electrochemical measurements indicated that 2 ML Pt/TiWC exhibits improved stability relative to Pt/C, with losses of 9% in ECSA, 22% in mass activity and 14% in specific activity after cycling. These observations demonstrate that complete Pt shell coverage can effectively suppress core dissolution and improve catalyst stability.

Three types of Pt–Ni nanoparticles supported on carbon were investigated, including core–shell Pt@Ni, Ni-rich PtNi, and Pt-rich PtNi, with mean particle sizes of 6.8 ± 1.3, 14.8 ± 5.2, and 6.3 ± 1.4 nm, respectively.^55^ AST, consisting of 1000 potential steps of 3 s between 0.6 and 1.0 V *vs.* RHE for ORR conditions and between 1.2 and 1.6 V *vs.* RHE for OER conditions in 1 M KOH, was applied. IL-TEM observations (Fig. 9i) showed that PtNi nanoparticles remain largely attached to the carbon support after cycling, whereas Pt/C exhibit significant nanoparticle detachment. The Pt-rich PtNi catalyst shows the highest particle loss, with 51% loss after ORR cycling and 24% loss after OER cycling. In contrast, Ni-rich and core–shell nanoparticles showed limited particle detachment. Morphological analysis revealed a transition from octahedral to more spherical shapes in Pt-rich nanoparticles, while Ni-rich and core–shell particles largely retained their morphology but exhibited the formation of filament-like features attributed to Ni oxide formation. A decrease in particle size was observed after stress testing, consistent with metal dissolution or exsolution. Electrochemical measurements indicated that Ni-rich and core–shell catalysts exhibit higher and more stable OER activity, whereas Pt-rich PtNi shows superior ORR performance. Structural and compositional analyses by XRD and XPS indicated phase segregation and surface oxidation after cycling, consistent with the changes observed by IL-TEM.

### 3.3 Oxygen evolution reaction (OER)

#### 3.3.1 Single-metal catalysts

Iridium nanoparticles (∼1.5 nm) dispersed on nanotubular TiON supports fabricated on Ti TEM grids were examined under electrochemical conditions using a floating electrode configuration in an H-cell,^58^ where a potentiostatic protocol switched between 1.55 and 0.78 V, applied for 5 min and 2 min, respectively. Identical-location TEM, STEM, and EELS analyses revealed that electrochemical treatment generated Ir single atoms while simultaneously inducing oxidation of the TiON support, evidenced by a decrease in the N/O ratio. IL-EELS measurements quantified the formation of a TiO_2_ surface layer of 3.0 ± 0.4 nm on bare TiON, whereas a significantly thinner layer of 0.3 ± 0.3 nm after electrocatalysis was measured for TiON-Ir, corresponding to an approximately tenfold reduction in oxide thickness. The smaller decrease in the N/O ratio in the presence of Ir coincided with reduced oxide growth, linking the emergence of Ir single atoms and nanoparticles to the suppression of oxidation of the TiON support under OER conditions.

Using a TEM grid, Ir nanoparticles (sub-nm to few nm) deposited *via* dip-coating of IrBr_3_ and subsequent reductive annealing on TiO_*x*_N_*y*_ supports were examined under OER conditions using a floating electrode configuration in an H-cell,^58^ with a pulsed protocol comprising twenty 5 min steps at 1.55 V *vs.* RHE separated by 2 min at 0.78 V. IL-STEM-HAADF imaging revealed agglomeration, detachment, Ostwald ripening, and the emergence of isolated Ir atoms (Fig. 10a and b) following electrochemical treatment, while statistical analysis showed that particle loss remained below 1% and size distribution metrics were largely preserved. In parallel, compositional analysis indicated a decrease in the N/O ratio of the TiO_*x*_N_*y*_ support, with a smaller variation than that observed for bare TiO_*x*_N_*y*_ under comparable conditions. Correlative measurements combining IL-STEM, IL-SEM, XPS, and Raman spectroscopy on the same TEM grid allowed concurrent tracking of nanoparticle evolution and support composition during catalyst synthesis and electrocatalysis tests.

**Fig. 10:**
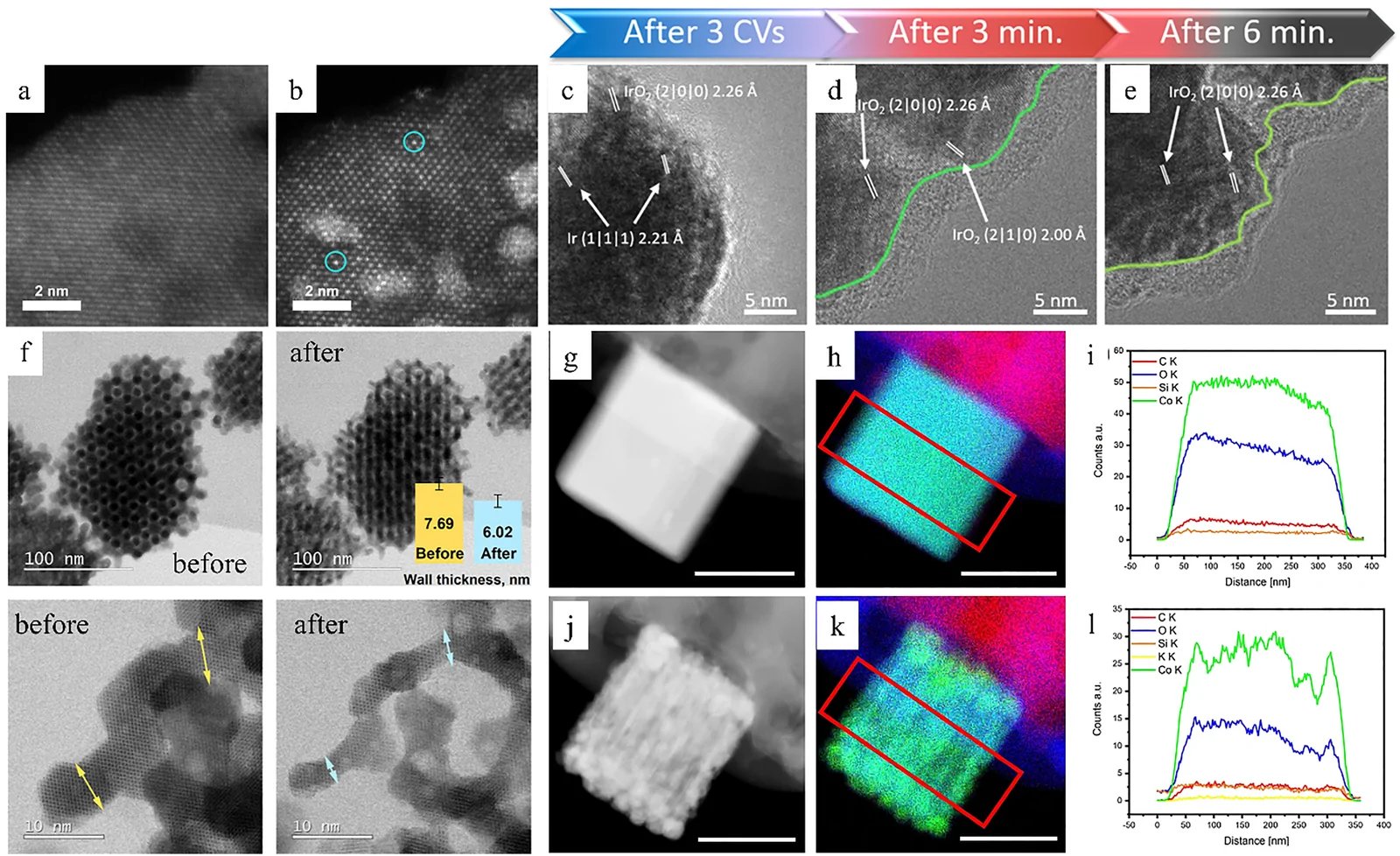
IL-STEM–HAADF image of the TiO_*x*_N_*y*_–Ir sample before (a) the addition of Ir and after (b) electrochemical treatment. Reproduced from ref. 58 with permission from the American Chemical Society, copyright 2023. IL-HRTEM images of the Ir50 Au@CNE UME at different stages: after the (c) initial 3 CV cycles, (d) 3 min, and (e) 6 min at an applied potential of 1.715 V *vs.* RHE. Reproduced from ref. 61 with permission from the American Chemical Society, copyright 2025. (f) High-resolution annular bright field IL-STEM of Co_3_O_4_ before and after the start-up/shutdown electrochemical protocols. Reproduced from ref. 64 with permission from the American Chemical Society, copyright 2025. Identical-location STEM image, EDS mapping and line profile of the Co_3_O_4_@CNE before (g)–(i) and after (j)–(l) the AST. Scale bar is 200 nm. Reproduced from ref. 65 from Springer Nature, copyright 2021.

Iridium nanoparticles supported on TiON_*x*_/C (3–4 nm) and Cu-modified TiON_*x*_/C (2–3 nm) were electrochemically activated by cycling (100 cycles, 0.05–1.45 V, 300 mV s^−1^) in 0.1 M HClO_4_.^59^ IL-STEM showed the development of an amorphous layer on Ir nanoparticles in both systems after activation, while the Cu-containing sample exhibited more pronounced particle fragmentation together with the emergence of isolated Ir single atoms on the support. These structural changes coincided with substantial Cu dissolution during treatment (∼65% of the initial Cu). The Cu-modified catalyst exhibited ∼35% higher OER mass activity, which was attributed to a higher density of accessible active sites resulting from smaller particle size and increased Ir surface area, with a further contribution from residual Cu species remaining in the structure.

Supported on TiON TEM grids, Ir nanoparticles with a broad size distribution (up to ∼15 nm) were investigated under potentiostatic activation (1.6 V, 30 min) followed by a dynamic degradation protocol consisting of 3 cycles of 60 repetitions at 5 mA cm^−2^ (30 s) and 0 mA cm^−2^ (45 s), with intermediate Ar purging in 0.1 M HClO_4_ at OCP and cyclic voltammetry (0.05–1.45 V, 300 mV s^−1^).^60^ IL-STEM observations indicated limited structural changes after activation, whereas the degradation protocol led to evident surface roughening in particles larger than ∼3 nm, reflected by the disappearance of well-defined facets and an increased population of low-coordinate atomic columns. In contrast, smaller particles underwent continuous reshaping without additional roughening. Occasional amorphous-like surface features were detected at specific sites. Quantitative nearest-neighbour analysis captured the redistribution of atomic coordination during degradation, consistent with the emergence of defect-rich surfaces, while electrochemical measurements showed increased OER current after activation and further enhancement following the degradation protocol, alongside a reduced oxidation tendency of TiON-supported Ir inferred from persistent metallic features in voltammetry.

Iridium films with controlled thickness were electrodeposited onto Au-supported carbon nanoelectrodes (CNEs) used as ultramicroelectrodes (UMEs) (denoted as Ir10, Ir20, and Ir50, corresponding to 10, 20, and 50 electrodeposition cycles, respectively) and subjected to three CV cycles up to 1.715 V *vs.* RHE, followed by potentiostatic holds at 1.715 V *vs.* RHE for 3 and 6 min in 0.1 M H_2_SO_4_.^61^ IL-HRTEM of Ir50 (Fig. 10c–e) after CV showed IrO_2_ lattice fringes localised near the surface while the metallic Ir lattice remained detectable, reflecting limited bulk structural change. After 3 min of polarisation, an amorphous surface layer emerged together with IrO_2_ lattice features, indicating surface oxidation, and further holding to 6 min resulted in stabilisation of this amorphous region. Comparative IL-SEM and IL-STEM analysis revealed substantial dissolution and structural disruption for Ir10, whereas Ir20 retained a more intact morphology with fewer structural changes. The persistence of the amorphous surface layer coincided with the presence of underlying metallic Ir and crystalline IrO_2_ sublayers, while the absence of such sublayers in thinner films corresponded to faster activity decay and reduced stability.

Porous IrO_*x*_ microparticles obtained by annealing of precursor-derived materials in air at 400 °C and 450 °C were probed by IL-TEM following electrochemical conditioning (100 cycles between 0.05 and 1.40 V *vs.* RHE) and subsequent potential cycling (200 cycles between 1.20 and 1.60 V *vs.* RHE at 60 °C in 0.05 M H_2_SO_4_), where IL-TEM imaging revealed no discernible change in morphology for both catalysts after cycling.^62^ This absence of particle-level restructuring coincides with divergent activity–stability metrics, as the 450 °C sample exhibits a stability number (*S*-number, defined as the ratio of evolved O_2_ to dissolved Ir)^63^ of ∼320 000, representing an approximately sixfold increase relative to commercial IrO_2_ and corresponding to the most favourable balance between OER activity and stability among the evaluated catalysts.

Ordered mesoporous Co_3_O_4_ with a pore size of ∼4.9 nm was investigated in Ar-saturated 0.05 M H_2_SO_4_ under start-up/shutdown cycling, constant potentiostatic holding at 1.75 V (400 min), and open circuit conditions.^64^ IL-STEM (Fig. 10f) revealed a consistent decrease in catalyst wall thickness after all electrochemical protocols, indicating material loss across the mesoporous framework. The thinning occurred uniformly throughout the structure, with no amorphous surface layer detected, and the crystalline spinel structure remained intact. These observations contrast with reports of surface amorphisation under OER conditions. Dissolution was observed both upon initial contact with the electrolyte and during OER operation, with isotope-labelled differential electrochemical mass spectrometry and dissolution measurements indicating lattice participation in the reaction.

Co_3_O_4_ cubes (∼207 nm) were investigated at the single-particle level under OER conditions using linear sweep voltammetry and accelerated stress protocols.^65^ At comparatively low current densities (∼1 A cm^−2^) with rapid potential sweeps (1 V s^−1^) in 0.05 M KOH, IL-HRTEM showed no detectable changes in morphology or lattice structure, with crystalline fringes extending to the particle surface. In contrast, AST conducted in 1 M KOH at a scan rate of 200 mV s^−1^ led to pronounced structural modifications, where IL-TEM and IL-EDS (Fig. 10g–l) revealed the disappearance of sharp cube edges and the emergence of internal Co_3_O_4_ agglomerates, indicating loss of compositional uniformity. Non-identical location HRTEM further showed that the particle interior retained its crystalline structure, while the near-surface region became amorphous. Under these conditions, current densities reached 5.5 A cm^−2^ with a corresponding turnover frequency of 2.8 × 10^4^ s^−1^ at 1.9 V *vs.* RHE.

Electrodeposited MnO_2_ sheet films consisting of complex clusters of particles, *ca.* 100–200 nm in size, supported on Au/C grids were examined after chronoamperometric holds at 1.5 and 1.7 V *vs.* RHE for 2 h in acidic electrolyte (HClO_4_, pH 1).^66^ IL-STEM combined with EDS and EELS revealed that the morphology remained largely unchanged at 1.5 V, whereas at 1.7 V the catalyst layer exhibited cracking accompanied by modest structural alterations. Compositional analysis showed that the normalised oxygen content remained constant at 1.5 V but increased by ∼4% after polarisation at 1.7 V, while EELS measurements indicated no detectable change in Mn oxidation state at 1.5 V and an increase toward higher oxidation states at 1.7 V, evidenced by energy shifts of the Mn L_2,3_ edges and a decrease in the *I*(L_3_)/*I*(L_2_) ratio. These structural and chemical changes coincide with *operando* scanning flow-cell measurements combined with inductively coupled plasma mass spectrometry, which detected negligible Mn dissolution at 1.5 V and measurable Mn dissolution at 1.7 V.

Ultramicroelectrodes (UMEs) based on gold microelectrodes with diameters of 12.7 and 50.0 µm were employed to investigate electrodeposited nickel selenide films under OER conditions in 0.1 M KOH with 1 mM Fe(NO_3_)_3_ using a multi-step electrochemical protocol that combines CV and impedance spectroscopy.^67^ At the smaller microelectrode (12.7 µm), where current densities exceeding 1 A cm^−2^ were achieved, IL-SEM imaging (Fig. 11a–f) revealed a pronounced transformation of the catalyst film morphology from initially spherical and ellipsoidal particles (<100 nm to several hundred nanometres) to predominantly flat, extended patches after OER, with most discrete particles no longer observed. In contrast, for the 50.0 µm microelectrode operating at significantly lower current densities (∼0.3 A cm^−2^), particle-based morphology remained clearly distinguishable after identical electrochemical treatment. Complementary IL-EDS analysis showed a reduction in selenium content accompanied by increased oxygen incorporation after OER, consistent with conversion of nickel selenide to nickel oxide/oxyhydroxide species. These structural changes were correlated with a decrease in mass-transport-limited current at high overpotentials, as identified from electrochemical measurements.

**Fig. 11:**
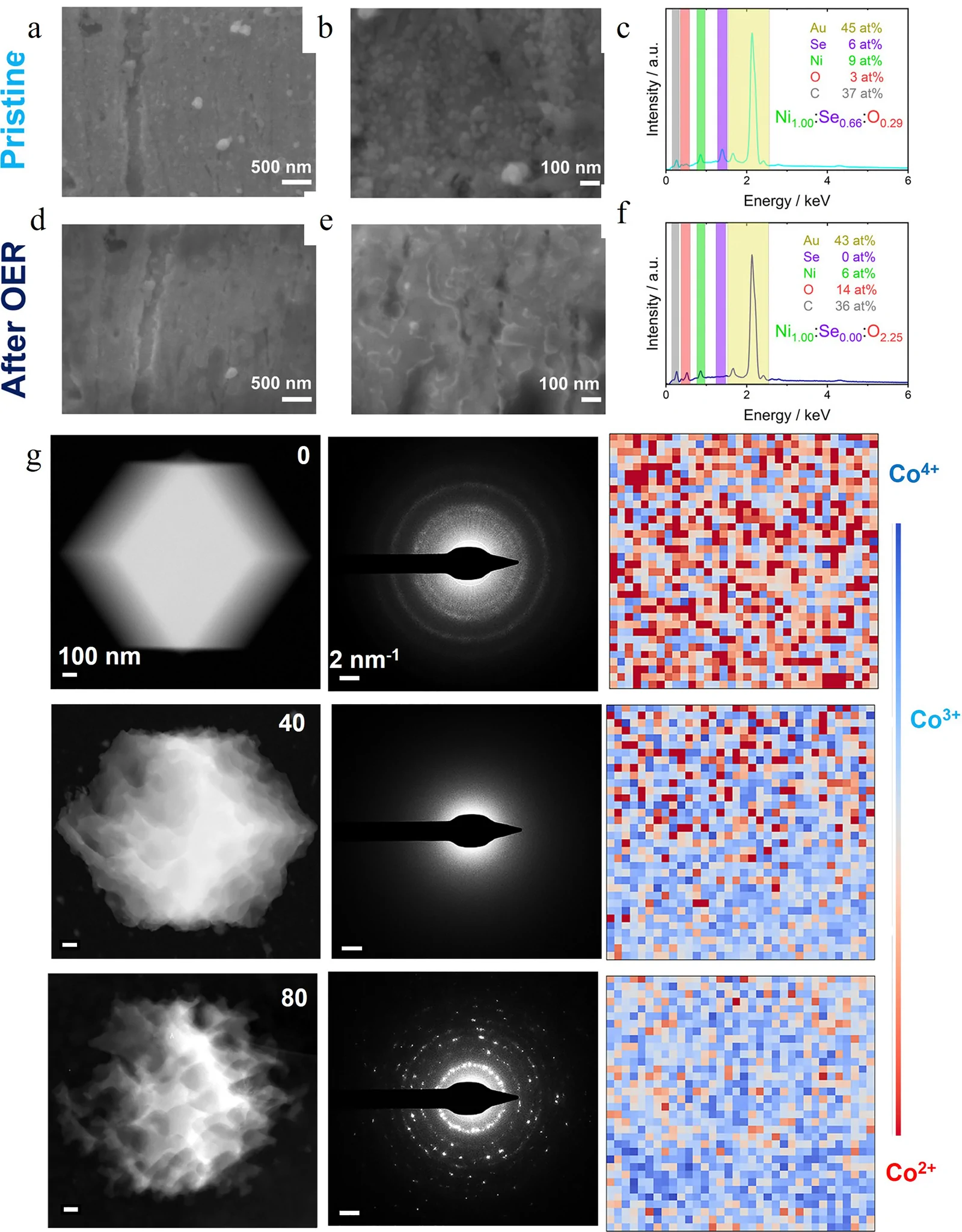
Identical-location SEM and EDS of a 12.7 µm UME of the (a)–(c) pristine-deposited film and (d)–(f) after OER testing. Reproduced from ref. 67 with permission from the American Chemical Society, copyright 2025. (g) IL-TEM images of a ZIF-67 particle during the OER. Adapted from ref. 68 with a figure provided by Sheng Dai.

ZIF-67 particles with sizes of 1.2–1.5 µm were investigated under OER conditions in 1.0 M KOH using IL-TEM at different electrochemical cycling stages (denoted as C*x*, where *x* represents the cycle number; *e.g.*, C0, C40, and C80 correspond to the 0th, 40th, and 80th cycles), with CV performed at 10 mV s^−1^ (0.1 to 0.7 V *vs.* Hg/HgO).^68^ IL-TEM imaging (Fig. 11g) revealed that the initial polyhedral framework underwent surface-initiated dissolution and oxidation during cycling, accompanied by the formation of a porous structure and a progressive loss of nitrogen as oxygen incorporation increased. Structural analysis showed transformation from the initial ZIF-67 framework to Co(OH)_2_, followed by the emergence of CoOOH and CoO phases, with electron diffraction and spectroscopic mapping confirming the coexistence of these species and a shift in cobalt oxidation state from Co^2+^ to Co^3+^. At later stages, the presence of Co(OH)_2_, CoOOH, and CoO persisted within the same particle, consistent with a dynamic phase equilibrium. These structural changes were accompanied by an increase in ECSA, reflected by double-layer capacitance values rising from 4.3 mF cm^−2^ for the initial sample (ZIF-67_0_, collected at cycle 0) to 24.0 mF cm^−2^ after 80 cycles (ZIF-67_80_), and improved OER performance, with ZIF-67_80_ exhibiting overpotentials of 326 and 415 mV at 10 and 100 mA cm^−2^ respectively, together with a Tafel slope of 67.4 mV dec^−1^. The observations from IL-TEM further suggest that catalytic performance is closely linked to the dynamic evolution of Co-based phases during operation, with the balance among Co(OH)_2_, CoOOH, and CoO determining both activity and long-term stability.

#### 3.3.2 Bimetallic and multimetallic catalysts

Nickel–aluminium layered double hydroxides with carbonate and bromide intercalation (LDH-CO_3_ and LDH-Br) were examined during OER in 0.1 M KOH under Fe-free conditions and with controlled Fe addition (0.01–1.00 ppm).^69^ IL-HAADF-STEM showed that the layered morphology was retained after electrochemical testing in both electrolytes, while in the presence of 0.10 ppm Fe, a weak but spatially distributed Fe signal was detected across the surface. The oxygen K-edge in EELS preserved its main spectral features after reaction, with only a broadened pre-edge component assigned to NiOOH formation, indicating limited structural modification. These observations are consistent with *ex situ* XAS. Despite the absence of measurable changes in electrochemically active surface area, the introduction of Fe led to a marked decrease in Tafel slope and a substantial increase in current density, suggesting enhanced OER kinetics facilitated by trace amounts of Fe.

**Fig. 12:**
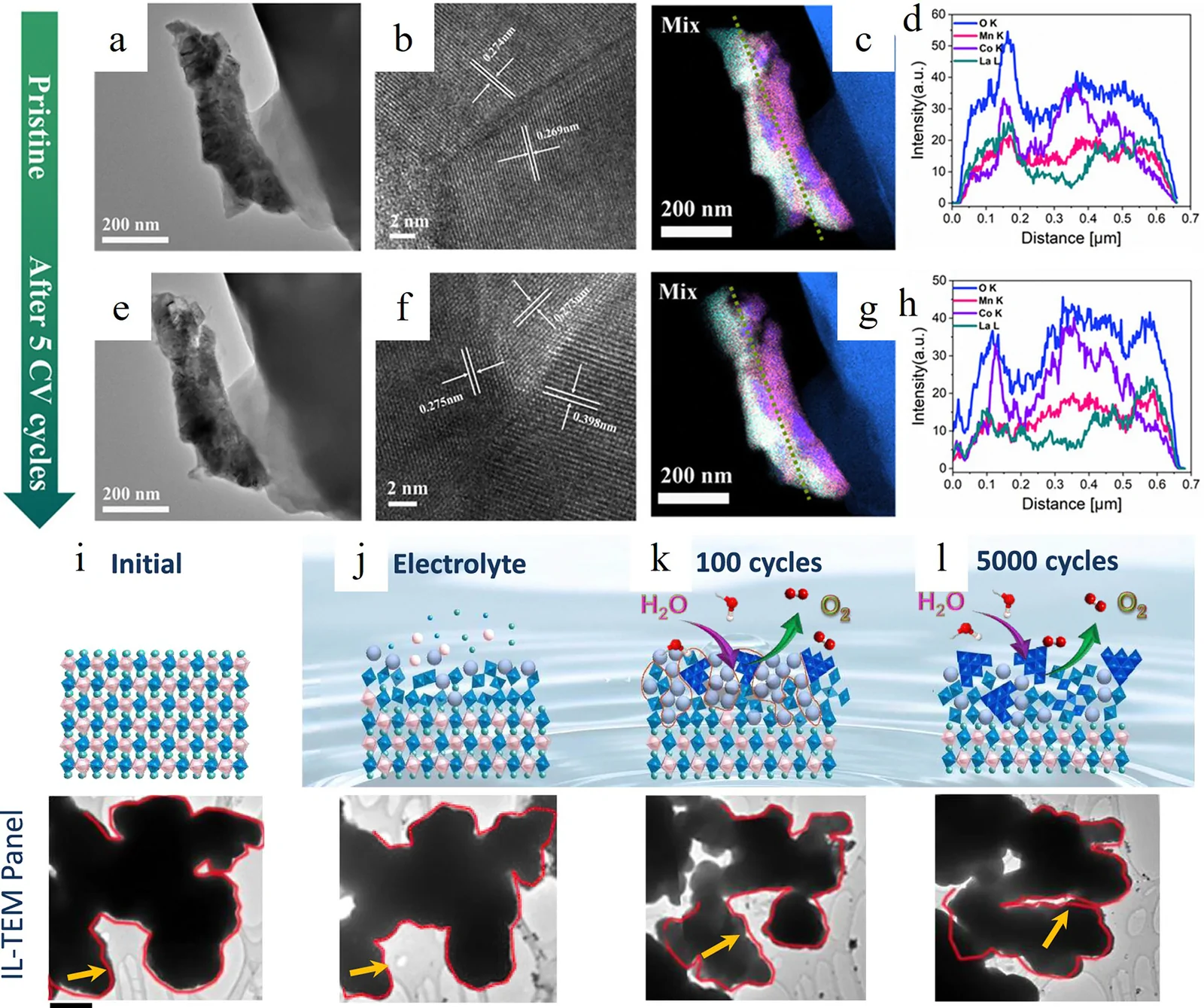
IL-TEM and IL-EDS of single La–Co–Mn–O column before (a)–(d) and after electrochemical measurements (e)–(h). Reproduced from ref. 70 with permission from John Wiley and Sons, copyright 2024. IL-TEM of (i) Fresh Sr_2_CaIrO_6_, (j) immersed in the electrolyte, (k) after 100 OER cycles and (l) after 5000 OER cycles. The scale bar is 500 nm. Reproduced from ref. 71 with permission from Springer Nature, copyright 2022.

La–Co–Mn–O columns (>100 nm in diameter and ∼1 µm in length) composed of LaCo_0.5_Mn_0.5_O_3_, Co_2_MnO_4_, and Co_3_O_4_ phases were examined under OER conditions using single-entity electrochemistry, in which individual columns are probed *via* a particle-on-nanoelectrode configuration, in purified 1 M KOH with five cycles between 1 and 1.8 V *vs.* RHE.^70^ IL-TEM combined with HRTEM and STEM–EDS mapping (Fig. 12 a–h) resolved the coexistence of multiple crystalline domains within individual columns, with lattice spacings corresponding to the three phases both before and after electrochemical testing. The spatial distribution of La, Co, and Mn remained unchanged, and no discernible variation in morphology or phase constitution was detected following cycling. Electrochemical measurements yielded ∼5 mA cm^−2^ at 1.7 V, while the current response showed no significant deviation over five cycles, consistent with the absence of structural or compositional evolution observed by IL-TEM.

Sr_2_CaIrO_6_, consisting of ∼30 nm crystallites aggregated into ∼300 nm particles, was examined in O_2_-saturated 0.1 M HClO_4_ using IL-TEM (Fig. 12 i–l) following electrolyte immersion and OER cycling (100 and 5000 cycles at 10 mV s^−1^ between 1.2 and 1.7 V *vs.* RHE, with the catalyst deposited on a glassy carbon electrode and measured under RDE conditions at 1600 rpm).^71^ After 5 min immersion, particles exhibited only a minor size reduction (∼5%) while maintaining morphology, accompanied by partial leaching of Ca and Sr as detected by EDS and ICP-OES, and the emergence of crystalline IrO_2_ together with amorphous IrOOH identified by selected area electron diffraction (SAED). After 100 cycles, the same regions evolved into hollow, open structures, where EDS indicated Ir–O compositions and SAED showed diffuse rings characteristic of IrOOH, while AC-STEM resolved short-range ordered domains of edge-sharing IrO_6_ octahedra. Prolonged cycling to 5000 cycles led to increased particle agglomeration and a higher frequency of open morphologies, with SAED halos at 2.7 and 1.5 Å becoming more diffuse, indicating progressive loss of local ordering. The development of these open structures was accompanied by an increase in mass-specific surface area relative to the pristine material, whereas the combined effects of agglomeration and structural disorder led to a 15% decrease in catalytic activity.

Ba_0.5_Sr_0.5_Co_0.8_Fe_0.2_O_3−*δ*_ (BSCF, where *δ* denotes the degree of oxygen non-stoichiometry associated with oxygen vacancy formation in the perovskite lattice) was examined after immersion in 0.1 M KOH for 3 h and subsequent CV between 1.1 and 1.8 V *vs.* RHE at 10 mV s^−1^ using IL-(S)TEM on MEMS-based platforms and liquid-cell configurations.^72^ IL-STEM and IL-EDS showed that Ba/Sr-rich secondary phases present on the as-prepared surface were removed upon alkaline immersion, accompanied by the emergence of a ∼20 nm Co/Fe-enriched surface region. EELS analysis of this region indicated a spinel-like structure with a modified Co oxidation state (Co^2+^) relative to the bulk (Co^3+^). Tracking identical particles before and after electrochemical treatment revealed that the overall particle morphology remained largely unchanged during cycling, whereas HAADF-STEM imaging revealed progressive surface roughening and the development of porosity with increasing exposure to cyclic voltammetry. The Co/Fe-rich spinel-like surface that persisted after electrochemical operation is associated with the formation of Co(Fe)OOH species under anodic conditions, correlating with the observed OER activity.

Nanorods of La_2_NiO_4_ were subjected to 300 electrochemical cycles in 1.0 M KOH, after which IL-TEM was performed on the same particle clusters.^73^ The images before and after cycling retained similar overall morphology, and no continuous surface layer could be resolved. Selected-area electron diffraction patterns remained largely unchanged, indicating the absence of detectable bulk structural transformation. In some regions, loss of local crystalline order was suggested by fast Fourier transform (FFT) analysis, although this was treated cautiously due to possible imaging artefacts and limited contrast from disordered surface species. Direct visualisation of the reconstructed phase by TEM remained unsuccessful, likely due to its limited thickness and heterogeneous distribution. Electrochemical measurements, including emerging Ni redox features and impedance-derived surface area changes, were used to infer the formation of redox-active surface species, as direct TEM evidence remained inconclusive.

Cysteamine·HCl-modified Ir–Ni mixed oxide nanoparticles (c-*x*NiIrMO, where *x* denotes the Ni molar fraction, 1.6–2.4 nm) were evaluated under CV cycling between 0.4 and 1.5 V *vs.* RHE in 0.5 M H_2_SO_4_ using a three-electrode configuration.^74^ IL-TEM imaging revealed composition-dependent structural evolution, where samples with Ni content (*x* > 0.5) underwent pronounced dissolution, consistent with Ni leaching during electrochemical activation, whereas Ir-rich compositions such as c-20NiIrMO displayed only limited particle shrinkage. For Ni-rich c-80NiIrMO, the removal of Ni species resulted in the formation of isolated Ir oxide nanoparticles with diameters of ∼1 nm, accompanied by evident detachment of these particles. This transformation coincided with a reduction in anodic charge (q*) for samples containing more than 40% Ni, linking the loss of structural integrity upon Ni dissolution to a decrease in accessible ECSA.

Nanoparticles of IrNi on carbon with sizes of 10–15 nm underwent electrochemical activation by 50 CV scans between 0.05 and 1.5 V *vs.* RHE at 500 mV s^−1^ in 0.05 H_2_SO_4_ (N_2_ purged), 0.5 M KHCO_3_ (CO_2_ purged), and 0.1 M KOH (N_2_ purged), yielding catalysts denoted as IrNiO_*x*_/C–A, IrNiO_*x*_/C–N, and IrNiO_*x*_/C–Al, respectively (where A, N, and Al refer to activation in acidic, neutral, and alkaline media). These catalysts were subsequently evaluated for OER in CO_2_-saturated 0.5 M KHCO_3_.^75^ IL-TEM combined with IL-STEM-EDS (Fig. 13a–i) revealed electrolyte-dependent structural evolution, where activation in acidic media produced a Ni-depleted IrNi@IrO_*x*_ configuration with Ni remaining only in the particle core, while alkaline treatment preserved Ni and generated a Ni-rich IrNiO_*x*_ shell without measurable loss of Ni; in contrast, pH-neutral conditions led to partial Ni removal confined to the surface, forming a thin and weakly oxidised IrO_*x*_ shell with an IrNi core. These transformations were accompanied by distinct CV responses consistent with the observed morphologies, and the catalyst activated under alkaline conditions exhibited a lower overpotential of 384 mV at 10 mA cm^−2^ in neutral electrolyte compared to the counterparts treated under acidic and neutral conditions. *Operando* XAS further resolved the electronic structure of IrNiO_*x*_/C–Al, where the Ir species exhibited a higher density of d-band holes, while Ni species were present in elevated oxidation states under OER conditions, consistent with its higher activity in neutral electrolyte.

**Fig. 13:**
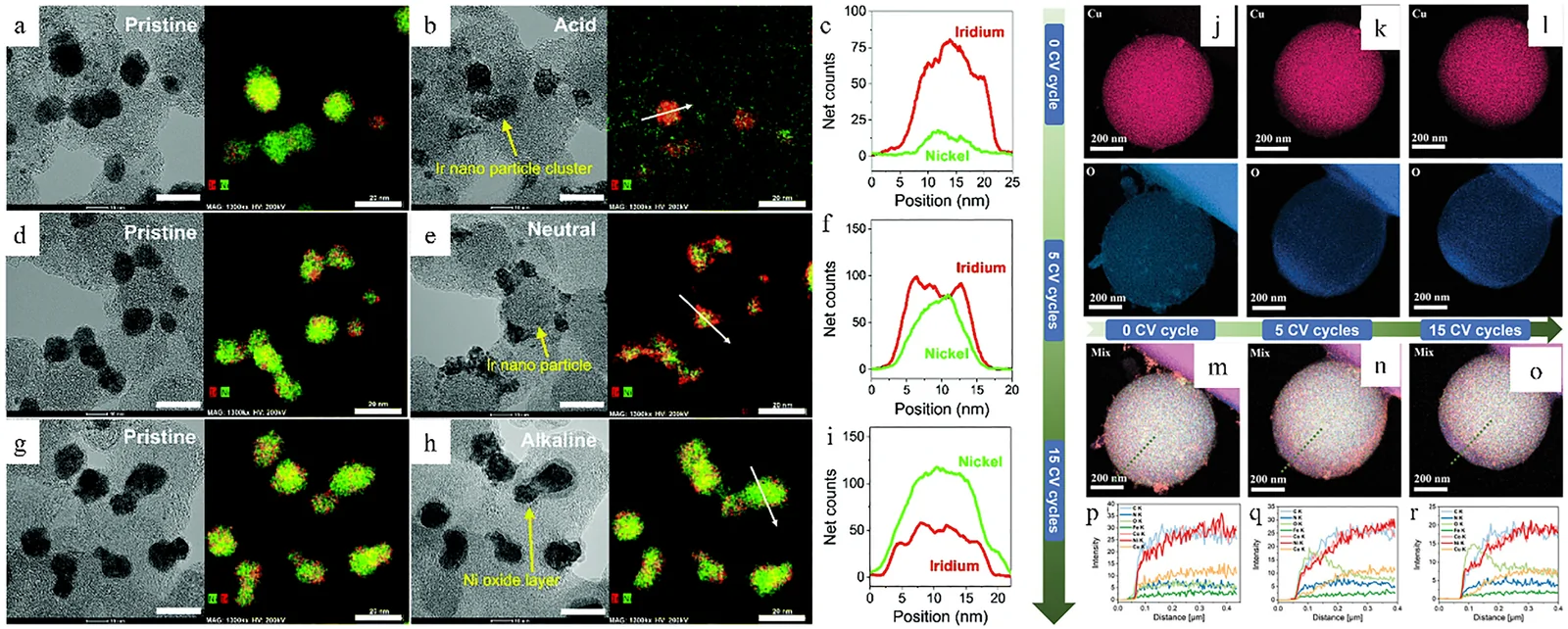
(a)–(i) Morphological and compositional transformation of electrochemically activated IrNi/C under acidic, neutral, and alkaline conditions. Reproduced from ref. 75 with permission from the Royal Society of Chemistry, copyright 2020. (j)–(o) Identical-location EDS elemental mapping and (p)–(r) EDS line scans of the single CoNiFeCu (1 : 1 : 1 : 0.5) particle before CVs, after 5 CVs, and after 15 CVs. Reproduced from ref. 78 with permission from John Wiley and Sons, copyright 2022.

Carbon-supported IrCo alloy nanoparticles (∼2–3 nm) and Ir nanoparticles (∼1–2 nm) have been examined in CO_2_-saturated 0.5 M KHCO_3_ (pH 7.4) within a potential range of 0.95–1.85 V *vs.* RHE.^76^ Following electrochemical oxidation, the catalysts were converted to IrCoO_*x*_/C and IrO_*x*_/C, respectively. IL-TEM imaging after a 216 h stability test at 5 mA cm^−2^ revealed pronounced particle growth and aggregation for IrO_*x*_/C, whereas IrCoO_*x*_/C retained a more homogeneous dispersion with significantly reduced agglomeration. Elemental analysis by non-identical TEM-EDS indicated that the Ir and Co ratio in IrCoO_*x*_/C remained close to its initial value after operation. These structural differences are consistent with electrochemical measurements, where IrCoO_*x*_/C exhibited improved stability and reduced Ir dissolution compared to IrO_*x*_/C, as confirmed by chronopotentiometry and *in situ*/*operando* ICP-MS.

A multicomponent NiFeCo-S/C catalysts, consisting of Fe- and Co-doped nickel sulfide nanoparticles supported on carbon and modified *via* H_2_S-assisted sulfurisation, were evaluated under oxygen evolution reaction conditions in 1 M KOH following electrochemical activation between 1.0 and 1.7 V *vs.* RHE in a 1 M KOH.^77^ IL-TEM and IL-EDS conducted after OER indicated that sulfur species, originating from the nickel sulfide phase, were partially retained within the catalyst despite surface leaching, as reflected by a decrease in the S/Ni ratio from 2.11 to 1.13. *In situ*/*operando* Raman and XAS further revealed that sulfur species persisted within the catalyst matrix and were associated with the formation of NiOOH during operation, accompanied by an increase in Ni–O bond length within the regenerated phase. These structural changes coincided with enhanced OER performance, where NiFeCo-S/C reached a current density of 10 mA cm^−2^ at an overpotential of 271.2 mV.

Multimetallic particles CoNiFeCu (1 : 1 : 1 : 0.5) of 0.45–0.95 µm were subjected to cyclic activation between 1.2 and 1.7 V *vs.* RHE at 100 mV s^−1^ in 1 M KOH, followed by IL-TEM and EDS analysis at different stages up to 2000 cycles.^78^ After 32 cycles, IL-EDS mapping (Fig. 13j–o) revealed the emergence of void-like features within the particles, accompanied by a reduction in Cu and N signals and a concurrent increase in O content, with these compositional and structural changes becoming more pronounced after extended cycling. Line-scan EDS further confirmed the progressive depletion of Cu and enrichment of O within the particles, while IL-TEM on carbon nanoelectrodes identified concurrent carbon corrosion during activation. These structural and compositional evolutions coincide with improved OER metrics, including a reduced overpotential of 291.5 ± 0.5 mV at 10 mA cm^−2^ and a Tafel slope reaching 43.9 mV dec^−1^ after activation, indicating that the observed Cu depletion and oxygen enrichment correlate with the enhanced catalytic response.

### 3.4 Hydrogen evolution reaction (HER)

The hydrogen evolution reaction (HER) is a crucial process in water splitting and other sustainable chemistry reactions, heavily dependent on catalytic processes at the nanoscale. Researchers have investigated rhodium nanoparticles measuring 2–4 nm with a 20% loading on a carbon support, which is a commercial catalyst.^79^ These nanoparticles were examined under accelerated degradation protocols (ADP) that involved 5000 rapid voltammetric scans (at a rate of 1 V s^−1^) in two potential ranges: 0.4 to −1.4 V *vs.* RHE, and 0.05 to −1.4 V *vs.* RHE in 0.1 M HClO_4_. An activation step was performed before the ADP, but the evolution of the catalyst during this step was not studied. No noticeable changes were detected in the narrower potential range by IL-SEM (Fig. 14a and b); however, changes at the nanometre and sub-nanometre scales could not be visualised due to the limited resolution of conventional SEM. In contrast, significant alterations were observed in the wider potential range. IL-SEM revealed many carbon particles that lacked Rh nanoparticles, while some displayed larger Rh nanoparticles compared to their size before the scans. The microscopy measurements were correlated with the activities of the HER and the ORR, particularly focusing on the ECSA. In the narrower potential range, the ECSA remained unchanged, while it decreased in the broader range. Additionally, the concentration of Rh in solution was measured using a scanning flow cell coupled with ICP-MS, revealing that approximately 0.03% of Rh dissolved in the narrower potential range, compared to around 1% in the wider range. Non-IL TEM imaging of the catalyst after the ADP showed that the original Rh nanoparticles had aggregated into larger clusters, with sizes increasing up to 15 nm. It is challenging to determine the mechanism behind the aggregation of nanoparticles using IL-SEM data alone, due to the lack of atomic resolution. However, the observation that dissolution is enhanced when using an RDE suggests that the coarsening of particles is primarily driven by the dissolution and subsequent re-deposition of rhodium. The differences observed in the two potential scan windows can thus be attributed to the reduction of the Rh oxide shell at potentials below 0.05 V in the wider scan, which leads to catalyst destabilisation.

**Fig. 14:**
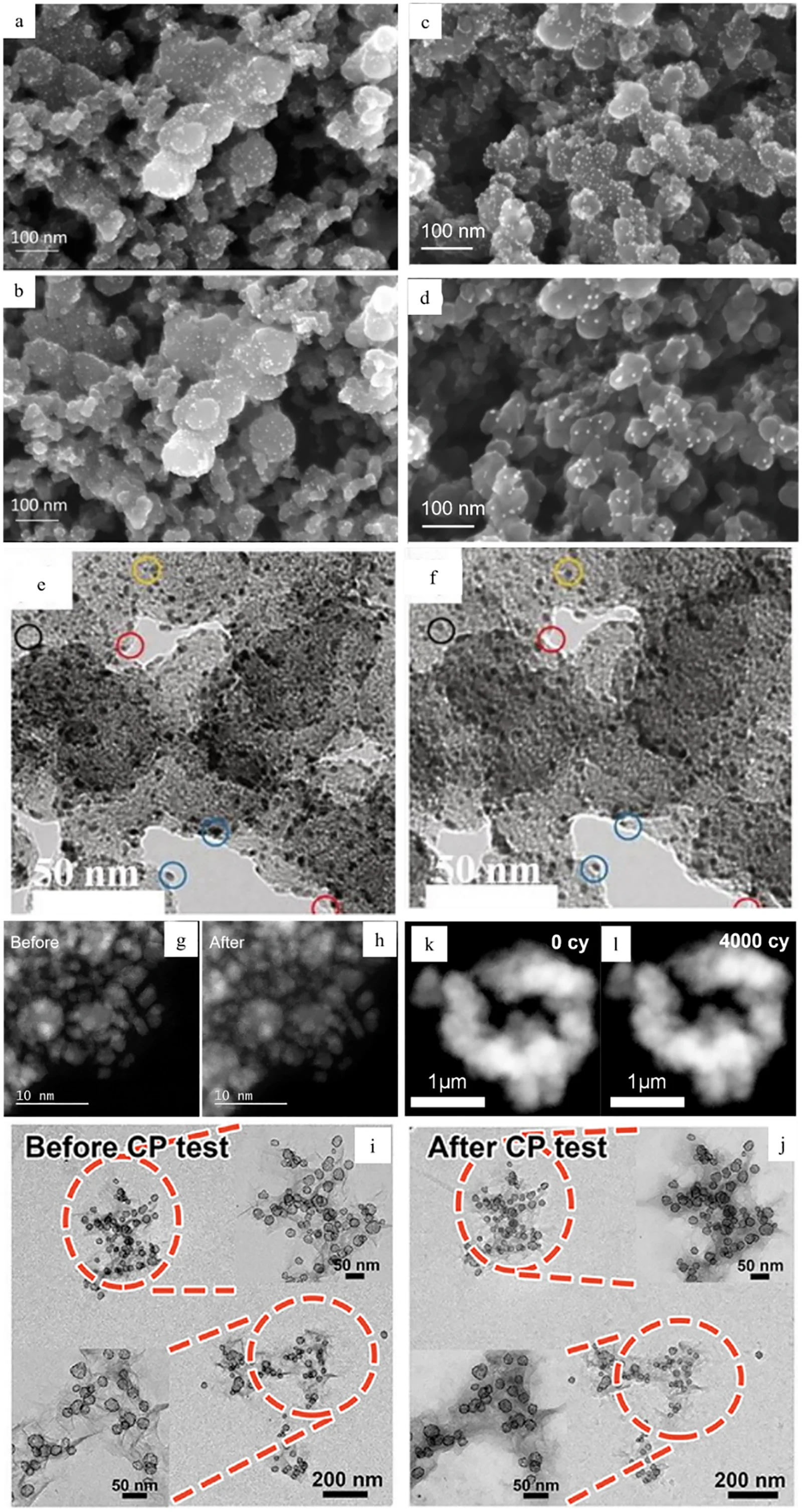
IL-SEM characterisation of the Rh/C: (a) before and (b) after the ADP. Reproduced from ref. 79 with permission from Elsevier, copyright 2021. IL-SEM inspection of the Pd/C: (c) before and (d) after AST. Scale bars correspond to 100 nm. Reproduced from ref. 80 with permission from the American Chemical Society, copyright 2021.IL-TEM characterisation of the ionic liquid@Pt/C before (e) and after (f) 2000 CV cycles between −0.1 V and 0.0 V *vs.* RHE with a scan speed of 50 mV s^−1^ in 0.5 M H_2_SO_4_ at room temperature. Reproduced from ref. 82 with permission from Elsevier, copyright 2021. IL-TEM characterisation of Pt/TAP before (g) and after (h) ADT. Reproduced from ref. 83 with permission from the American Chemical Society, copyright 2022. IL-TEM images before (i) and after (j) HER of Pt/Ni(OH)_2_. Reproduced from ref. 84 with permission from John Wiley and Sons, copyright 2025. IL-STEM characterisation of Re_0.2_Mo_0.8_S_2_ before (k) and after (l) 4000 CVs. Reproduced from ref. 85 with permission from the American Chemical Society, copyright 2023.

Another commercial catalyst consisting of palladium nanoparticles measuring 2–6 nm on carbon, with a loading of 20%, was subjected to an AST.^80^ This involved applying 5000 rapid CV scans at a rate of 1 V s^−1^ in a 0.1 M HClO_4_ electrolyte within the potential window of 0.4 to −1.4 V *vs.* RHE, or alternatively, from 0.05 to −1.4 V *vs.* RHE. IL-SEM reported that the nanoparticles experienced varied changes; some disappeared at certain sites while others increased in size, ultimately becoming 2–3 times larger than their original dimensions before the test. When examined in the narrower potential window, the nanoparticles retained their morphology (Fig. 14c and d). However, in the wider potential window, palladium transformed into crystalline structures approximately 100 nm in size, with shapes differing from the original nanoparticles. These new crystals were developed on top of the catalyst. The alterations in the catalyst were accompanied by a decrease in HER activity and the ECSA of the palladium. Notably, there was significant palladium dissolution during the CV cycle. The 3D Ostwald ripening mechanism has been proposed to explain the coarsening of the nanoparticles. This process involves the dissolution of smaller nanoparticles followed by their re-deposition onto larger nanoparticles, resulting in increased size and an overall loss of active surface area.

To further elucidate the degradation behaviour of carbon-supported noble metal catalysts under HER conditions, both Pd/C and Pt/C systems have been examined.^81^ Commercial Pd/C and Pt/C catalysts, typically supported on Vulcan XC72 carbon, with average particle sizes of 3.6 ± 0.7 and 3.0 ± 0.7 nm, respectively, have been studied. Standard CV or ageing protocols, including 100 potential cycles between −0.05 and 0.60 V *vs.* RHE at a scan rate of 2 mV s^−1^ in Ar-saturated 0.1 M HClO_4_ at 25 °C, have been employed to assess catalyst stability. For Pd/C, more aggressive square-wave cycling between −0.10 V and 0.40 V (with 10 s dwell time at each potential) was utilised. Notably, a preconditioning step is applied prior to CV or ageing procedures, yet catalyst evolution during this phase remains poorly characterised. IL-TEM studies suggested that observed metal loss does not stem from metal dissolution or nanoparticle detachment from the carbon support, but rather from partial delamination of the thin-film catalyst layer. Complementary ICP-MS measurements revealed that the extent of metal release is closely linked to HER activity, with Pt releasing more metal than Pd and enhanced under lower-potential limits and slower sweep rates. Such release behaviour is exclusive to carbon-supported nanoparticles, with no detectable loss observed for bulk Pt or Pd electrodes. The corresponding online ICP-MS signals exhibited characteristic spiking behaviour, similar to that observed from *ex situ* injections of metal/C catalyst suspensions. These findings highlight the critical need to distinguish between true metal dissolution and catalyst layer detachment when interpreting degradation data. Approaches to enhance the stability of the catalyst layer and mitigate detachment from the working electrode include decreasing the catalyst layer thickness and increasing the ionomer-to-carbon ratio.

The stability of platinum nanoparticles, with a mean diameter of 2.6 nm and a 20% loading on carbon (a commercial catalyst), was tested before and after modification with an ionic liquid.^82^ The evaluation was performed over 2000 CV cycles between −0.1 V and 0.0 V *vs.* RHE, using a scan rate of 50 mV s^−1^ in a 0.5 M H_2_SO_4_ solution. IL-TEM revealed that the ionic liquid coating protects the nanoparticles from detachment, growth, and agglomeration (Fig. 14e and f). It is hypothesised that the ionic liquid safeguards the platinum surface by creating a hydrophobic environment, which helps suppress dissolution in water.

A broad distribution of platinum particles, including nanometre-sized clusters, larger nanoparticles, and nanowires, was deposited on a tris-(aza)-pentacene (TAP) support material at a loading of 16%.^83^ These were studied under an HER degradation protocol using a 0.1 M HClO_4_ electrolyte, with 3000 voltammetric scans conducted between 0.05 and 1.4 V *vs.* RHE at a scan rate of 1 V s^−1^. Imaging with IL-TEM revealed no significant changes in the particles (Fig. 14g and h), which correlated with both the ECSA and activity of the catalyst during HER. The stability of the catalyst was attributed to the presence of nitrogen atoms and the nanotextured nature of the support material, which helps stabilise the platinum nanoparticles. This contrasts sharply with platinum supported on commercial carbon, which is a less stable catalyst under the same testing conditions.

A porous Ni(OH)_2_ layer was wrapped around Pt nanocages – hollow Pt structures (30–50 nm) formed *via* a galvanic replacement–induced Kirkendall effect – to regulate interfacial charge transfer.^84^ The Pt/Ni(OH)_2_ and commercial Pt/C (carbon-supported Pt nanoparticles) were cycled 5000 times in a potential range between −0.95 and 1.00 V at a sweep rate of 10 mV s^−1^ in N_2_-saturated 1.0 M KOH. After 5000 cycles, IL-TEM reveals that Pt/C undergoes Pt nanoparticle detachment from the carbon support, accompanied by Pt nanoparticle coarsening *via* Ostwald ripening, increasing the mean particle size from 2.80 to 3.24 nm. In contrast, Pt nanocages remain highly dispersed within the Ni(OH)_2_ nanosheets after cycling, indicating preserved structural integrity (Fig. 14i and j). Consistently, the overpotential of Pt/C changes by 27 mV at 100 mA cm^−2^ after cycling, whereas Pt/Ni(OH)_2_ remains essentially unchanged. These observations demonstrate that the encapsulation-confinement structure suppresses active-site migration and particle size evolution, thereby stabilising the catalyst under alkaline HER conditions.

Mixed metal particles composed of Re_0.2_Mo_0.8_S_2_, referred to as nanoflowers, were immobilised in a Nafion binder on gold TEM grids.^85^ These nanoflowers were cycled 4000 times between 0 and −0.25 V *vs.* RHE in a 0.5 M solution of sulfuric acid. Using IL-STEM (Fig. 14k and l) for imaging, researchers detected no structural changes in the catalyst, even at the nanometre scale. However, IL-EDS showed a decrease in oxygen content, while the rhenium content remained unchanged. The study also investigated various methods for connecting the TEM grid to the working electrode, addressing the issue of hydrogen bubble formation, which poses a challenge and limits the range of experimental conditions that can be explored using identical-location microscopy for hydrogen evolution reactions.

Nanoparticles of Ag_60_Au_40_ with an average diameter of 11 ± 3 nm, synthesised from reverse micelles and immobilised on a boron-doped diamond electrode (thinned to electron beam transparency),^86^ were examined using IL-STEM and EDS under potentiodynamic dealloying conditions in 1.0 M HClO_4_ between 0.0 and 1.25 V *vs.* Ag/AgCl up to 100.5 cycles. Identical-location imaging enabled tracking of more than 70 individual particles, revealing significant compositional and morphological evolution during cycling. The largest change occurred during the initial cycle, where particles lost approximately 40 ± 16% of their Ag content, followed by a continued decrease to 18 ± 9 at% over the following 100.5 cycles. Concurrently, particle diameters decreased from 11 ± 3 to 6 ± 2 nm, while most particles retained a spheroidal morphology throughout the treatment. In addition to gradual shrinkage, particle loss due to dissolution or detachment was observed, and aggregated particles underwent coalescence into more compact structures. The evolution patterns of individual particles exhibited substantial heterogeneity in both size and composition. Correlation with macroscopic electrochemical measurements and scanning electrochemical cell microscopy indicated that catalytic activity toward hydrogen evolution increased after initial dealloying and decreased upon extended cycling.

### 3.5 Electrochemical activation, stability and degradation

Sulfur-doped carbon-supported Pt single-atom catalysts (Pt SACs) have been investigated under AST involving 1000 cycles of an extended load AST (0.05 to 1.00 V *vs.* RHE) and a combined start–stop/load AST (0.6 to 1.5 V *vs.* RHE) in 0.1 M HClO_4_.^87^ IL-HAADF-STEM imaging (Fig. 15a and b) after the extended load AST showed that most Pt atoms remained present and largely preserved their initial spatial positions, indicating minimal structural perturbation. In contrast, after the combined start–stop/load AST, IL-TEM revealed clear evidence of Pt dissolution together with the formation of larger clusters arising from the aggregation of initially isolated atoms. Additionally, new single Pt atoms appeared at previously unoccupied locations, which was attributed to either dissolution–redeposition processes or surface atom mobility, although the two could not be distinguished. These structural changes correlate with electrochemical measurements showing that, within the lower-potential window, Pt SACs exhibit dissolution behaviour comparable to that of conventional carbon-supported Pt nanoparticles (Pt/C). At higher anodic potentials, however, measurable Pt dissolution occurs (Fig. 15c and d), although the overall extent of Pt loss remains lower than that observed for Pt/C under similar conditions. Combined XPS and ICP-MS analyses further indicate that these transformations are closely linked to changes in the sulfur-doped carbon support. In particular, substantial sulfur depletion was observed during electrochemical cycling, indicating progressive destabilisation of Pt–S coordination sites. The loss of these anchoring moieties is therefore likely a key factor driving the redistribution, aggregation, and partial dissolution of Pt species observed under harsh operating conditions.

**Fig. 15:**
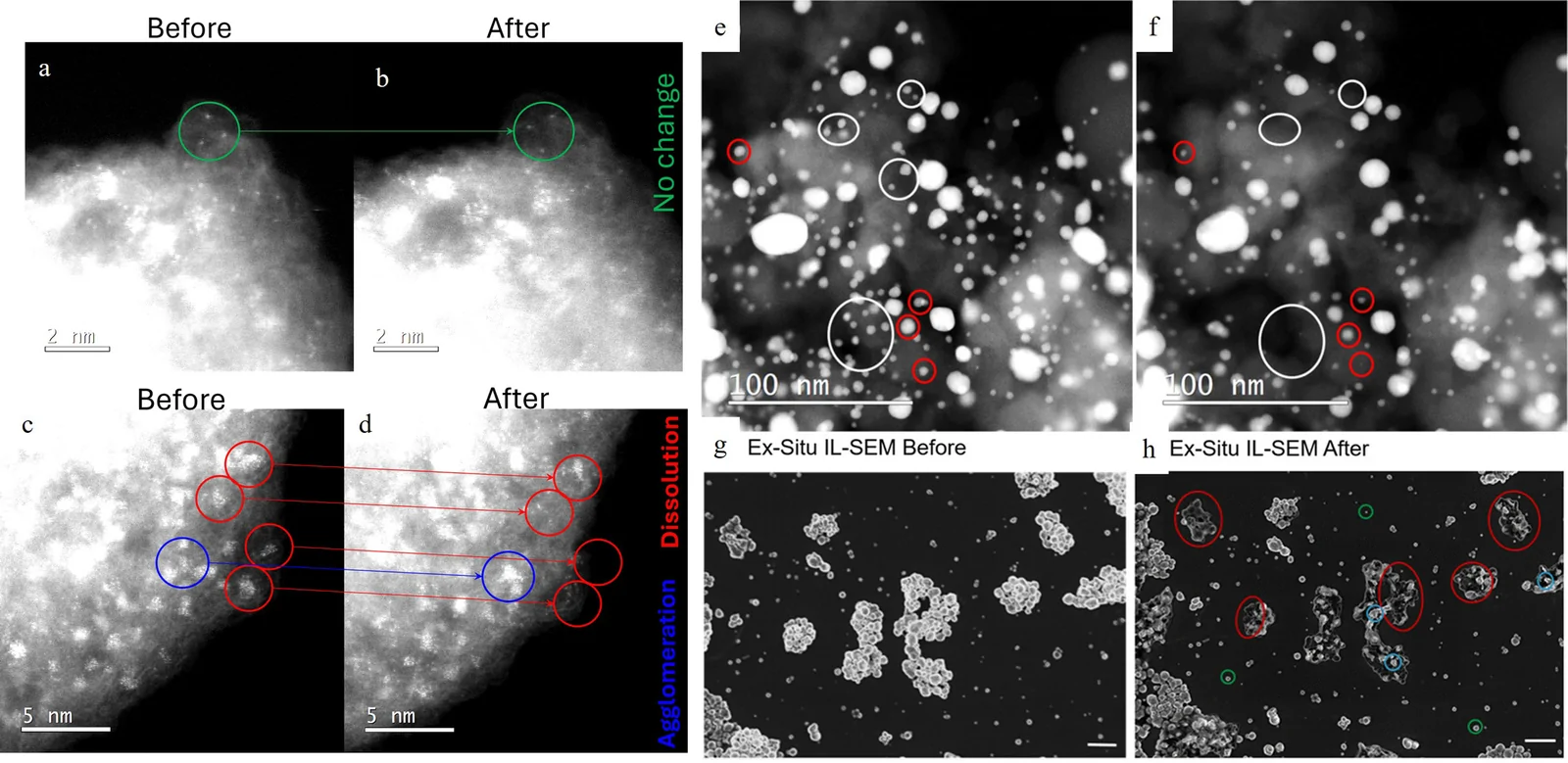
IL-HAADF images before (a) and (c) and after (b) and (d) 1000 cycles of an extended load AST (a and b, from 0.05 to 1.0 V *vs.* RHE) and a combined start–stop/load AST (c and d, from 0.6 to 1.5 V *vs.* RHE). Scale bars are 2 nm (a) and (b) and 5 nm (c) and (d). Adapted from ref. 87 with a figure provided by Serhiy Cherevko and Nejc Hodnik. IL-TEM imaging of the Au/C sample: (e) before and (f) after AST. Reproduced from ref. 88 with permission from the American Chemical Society, copyright 2021. IL-SEM images of the NPs before (g) and after (h) exposure to the ozone-saturated electrolyte. The scale bar is 2 µm. Reproduced from ref. 89 with permission from John Wiley and Sons, copyright 2024.

Carbon-supported Au nanoparticles with a polydisperse size distribution were subjected to 10 000 voltammetric scans (1 V s^−1^) between 0.58 and 1.41 V *vs.* RHE in 0.05 M H_2_SO_4_, in the absence or presence of 10^−4^ or 10^−2^ M Cl^−^.^88^ IL-SEM imaging in chloride-free electrolyte showed no clearly discernible morphological changes during the AST and no evidence of particle growth, excluding redeposition as a contributing mechanism. In contrast, IL-TEM imaging (Fig. 15e and f) revealed that a significant fraction of particles smaller than 5 nm disappeared after the AST, while larger particles remained largely unaffected, identifying size-dependent dissolution as the dominant structural change. This selective loss of sub-5 nm particles corresponds to a marked reduction in ECSA, with only ∼35% of the initial value remaining despite minimal overall Au mass loss, consistent with the disproportionate contribution of small particles to ECSA. In electrolytes containing chloride ions, IL-SEM showed progressively more pronounced particle loss, with substantial disappearance of particles already at 10^−4^ M Cl^−^ and near-complete removal at 10^−2^ M, which correlates with further decreases in ECSA to ∼20% and below 1% of the original value, respectively.

The process of gold stripping during the recycling of electronic components was simulated using 500–800 nm gold particles, which were drop-cast onto a glassy carbon electrode and then coated with a Nafion binder.^89^ IL-SEM was employed to study the evolution of the particles after exposure to an ozone-saturated solution of 0.1 M H_2_SO_4_ and 0.2 mM HCl at an OCP over a duration of 2.5 h. IL-SEM imaging (Fig. 15g and h) revealed that the gold particles began to dissolve, and their shapes became non-spherical during the process. EDS was used to analyse the residual particles after treatment, enabling distinction between gold-containing particles and carbon residues. Additionally, ICP-MS monitored the amount of dissolved gold in the solution, providing important complementary information. The investigated process has the potential to replace cyanide-based methods, providing an environmentally friendly alternative for recycling precious metals from electronic waste.

### 3.6 Electrochemical ammonia synthesis

The electrochemical reduction of water-soluble nitrogen-containing compounds, such as nitrates or nitrites, presents a promising method for transforming pollutants or waste materials into valuable products, specifically ammonia. There is a growing demand for ammonia as a hydrogen storage medium and as a fuel to help decarbonise industry and transportation; therefore, the development of catalysts for electrochemical ammonia synthesis is becoming increasingly important. Individual cubes of Cu_2_O and Co_3_O_4_, measuring 200–300 nm, were studied for their effectiveness in reducing nitrate on a specially designed carbon nanoelectrode.^90^ Five CV cycles were performed between +0.4 and −0.5 V *vs.* RHE in a 0.1 M NaOH solution containing either 0.1 M NO_3_^−^ or 0.1 M NO_2_^−^, at a scan rate of 50 mV s^−1^. Monitoring the changes in individual particles of the catalyst using IL-STEM and IL-STEM-EDS (Fig. 16a) revealed significant alterations in Cu_2_O, attributed to etching by the NO_2_^−^ intermediate and copper leaching. EDS indicated a reduction in the oxygen peak, suggesting the reduction of Cu_2_O to copper metal. As a result, the Cu_2_O cubes became porous and damaged while still maintaining their overall geometry. In contrast, changes in Co_3_O_4_ were less pronounced. Identical-location microscopy data were correlated with nitrate reduction current at −0.35 V *vs.* RHE, which showed an increase when the Cu_2_O and Co_3_O_4_ cubes were positioned next to each other. Interestingly, IL-STEM-EDS revealed no alloying between the two metals. This led to the proposal of a tandem catalysis mechanism, where the reduction of nitrate to nitrite occurs on the Cu_2_O particle, followed by the reduction of nitrite to ammonia on the Co_3_O_4_ particle. Overall, the co-location of Co_3_O_4_ and Cu_2_O significantly enhanced the conversion from NO_3_^−^ to NH_3_, achieving a faradaic efficiency of 85% at −0.3 V *vs.* RHE, with the catalyst's activity increasing over time, which was explained by IL-STEM measurements.

**Fig. 16:**
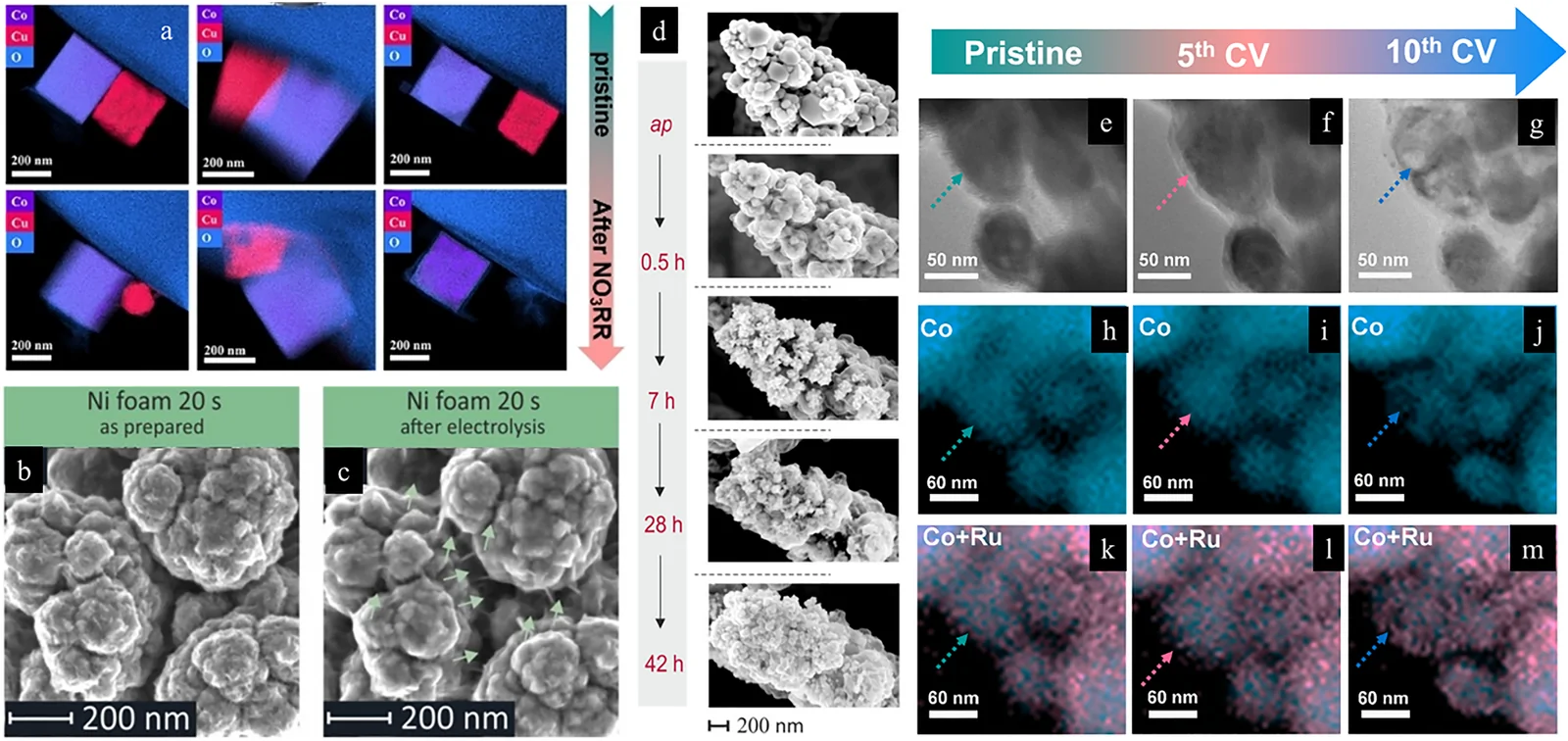
(a) EDS mapping of Cu_2_O + Co_3_O_4_ on a carbon nanoelectrode before (top) and after 5 CV cycles (bottom).^90^ Reproduced from ref. 90 with permission from John Wiley and Sons, copyright 2023. IL-SEM analysis of the Ni foam catalyst (20 s deposition time) before (b) and after electrolysis (c) (30 min at −0.3 V *vs.* RHE). Reproduced from ref. 91 with permission from Elsevier, copyright 2022. (d) IL-SEM characterisation of the Cu-foam (30 s)@mesh catalyst as a function of the electrolysis time. Reproduced from ref. 92 with permission from the American Chemical Society, copyright 2023. TEM images (e)–(g), elemental Co EDS map (h)–(j), and Co/Ru overlay EDS map (k)–(m) of CNE@Co–B/Ru_12_ before (first column), after the 5th (second column), and after the 10th CV cycle (third column), measured in 0.1 mol L^−1^ NaOH containing 0.1 mol L^−1^ NaNO_3_ at a scan rate of 50 mV s^−1^. Reproduced from ref. 93 with permission from Springer Nature, copyright 2024.

Metal catalysts with a foam-like morphology have proven to be effective for electrochemical ammonia synthesis. For instance, nickel foams of varying thicknesses were electrodeposited onto a nickel foil and subjected to a potential of −0.3 V *vs.* RHE for 30 min in a solution containing 1 M KOH and 0.1 M KNO_3_, all under an argon atmosphere.^91^ IL-SEM analysis indicated no significant microscale changes; however, there was a slight growth of nanoscale structures atop the microparticles (Fig. 16b and c). Additionally, cross-sectional SEM-EDS mapping was effectively used to examine the penetration of potassium ions through the foam. The faradaic efficiency for ammonia production decreased over time, which was attributed to the depletion of nitrate anions within the foam.

Copper foam with microscale pores and dendritic structures, measuring hundreds of nanometres, was electrodeposited onto a copper mesh and studied at −0.7 V *vs.* RHE in a 100 mM KNO_3_ solution within a KOH electrolyte (pH 14) over a duration of 42 h.^92^ IL-SEM revealed no significant changes in the overall microstructure of the catalyst; however, there were notable alterations on the surface of the dendritic structures (Fig. 16d). These changes resulted in increased nanoscale roughness and the formation of uncoordinated sites on the catalyst. The nanoscale modifications contributed to an increase in the hydrogen evolution reaction rate, competing with the ammonia production. However, the generation of hydrogen gas bubbles enhanced the mass transport of nitrate anions to the catalyst surface, leading to a high partial current density for NH_3_, despite a decrease in the faradaic efficiency for NH_3_ production. Overall, the catalyst demonstrated excellent stability during electrochemical ammonia synthesis.

A catalyst with a complex composition, Co-B/Ru_12_, was studied for electrochemical ammonia synthesis. This catalyst was investigated by identical-location microscopy as a single microparticle that contains various regions of Ru metal and areas of an amorphous Co-B phase.^93^ The ammonia synthesis process was carried out in a solution of 0.1 M NaOH with 0.1 M NaNO_3_, using five or ten CV cycles between potentials of 0.9 and −0.3 V *vs.* RHE. IL-TEM and IL-EDS revealed no change in the shape or overall structure of the microparticles during the experiments (Fig. 16e–m). However, there was a noticeable redistribution of Co, including a loss of Co from certain areas. This led to a continuous increase in the number of active sites in the catalyst as the number of CV scans increased. The single-particle electrochemistry measurements established the faradaic efficiency for ammonia production, which reached an impressive 90.4 ± 9.2%. Additionally, the catalyst achieved a remarkable half-cell energy efficiency of 40.9 ± 4% at 0 V *vs.* RHE.

### 3.7 Electrochemical formic acid oxidation

A catalyst for the electrochemical oxidation of formic acid was developed using a mixture of 2 nm platinum and 10 nm gold nanoparticles.^94^ This was accomplished through colloidal synthesis, yielding nanoparticles of distinct sizes to facilitate differentiation in EM images. The nanoparticles were co-deposited on carbon black. In the GDE setup, a gold TEM finder grid with the catalyst on support was positioned between a Nafion membrane and the gas diffusion layer, all immersed in a 1 M HClO_4_ electrolyte with formic acid delivered as vapour. The catalyst underwent potential cycling between 0.15 and 1.20 V *vs.* RHE for 50 cycles at a scan rate of 50 mV s^−1^. The changes in the catalyst were examined using IL-TEM (Fig. 17). The results indicated that platinum nanoparticles migrated and coalesced with gold nanoparticles, or dissolved, as evidenced by the shrinking of Pt nanoparticles observed in IL-TEM imaging. Importantly, the rate of formic acid oxidation improved due to the formation of a Pt–Au alloy, which lowered the overpotential and enhanced the catalyst's resistance to poisoning. This conclusion was further supported by *operando* small-angle X-ray scattering (SAXS), elemental mapping in STEM, and wide-angle X-ray scattering (WAXS), all of which confirmed the formation of the Pt–Au alloy detected in IL-TEM.

**Fig. 17:**
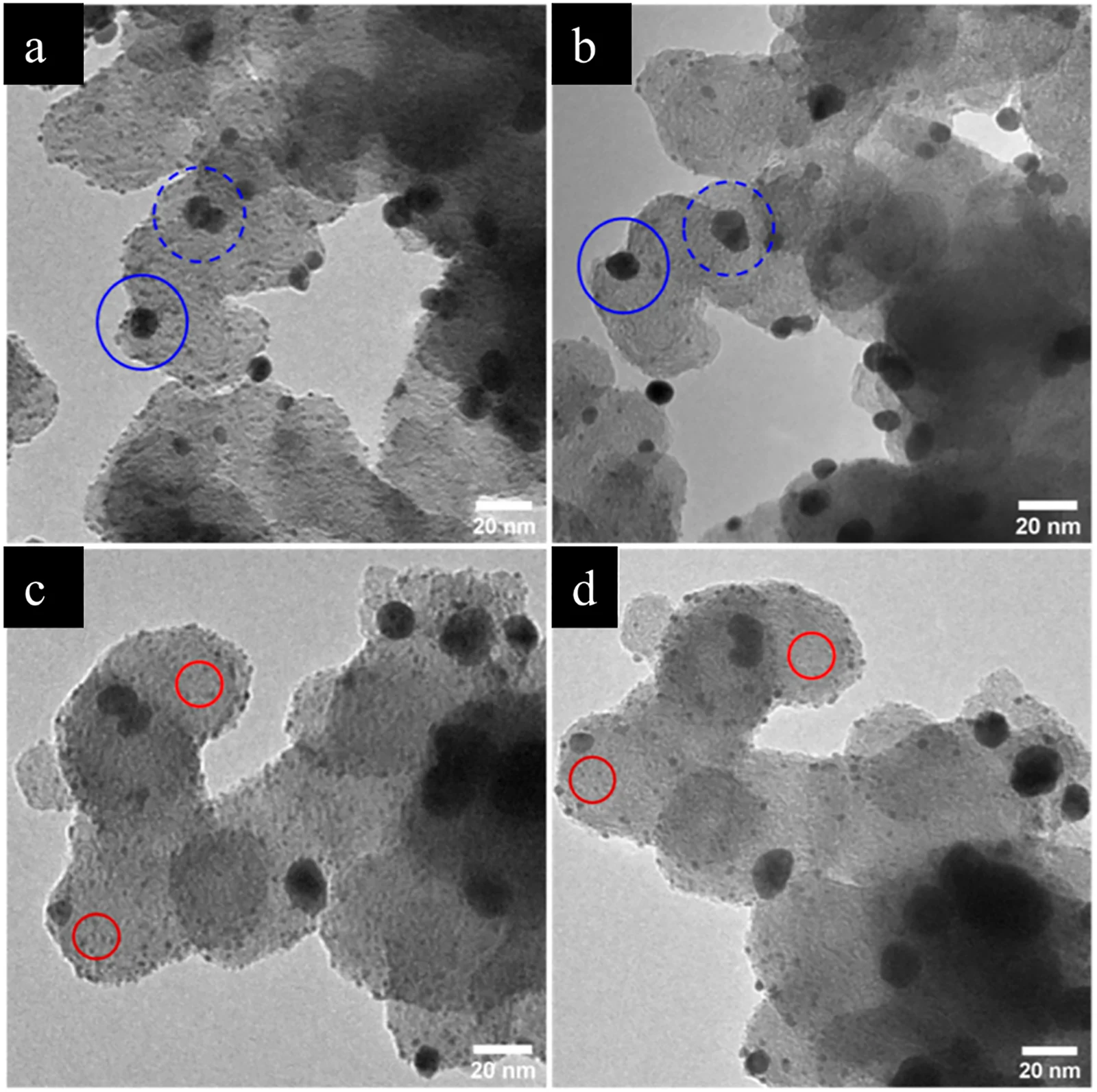
IL-TEM (a)–(d) of the Pt + 5Au/C nanocomposite: pristine (a) and (c), and the same sample exposed to 50 CVs in the FAOR potentiodynamic test (b) and (d).^94^ Reproduced from ref. 94 with permission from the American Chemical Society, copyright 2022.

### 3.8 Fuel cell

Platinum on carbon support is the most commonly used electrocatalyst in fuel cells due to its high activity in ORR and OER, and stability. However, feedstocks of Platinum are very limited, making this metal expensive, and there are challenges associated with degradation of the catalyst in the fuel cell. For example, the high and changing potentials during fuel cell operation facilitate corrosion and dissolution of Pt, particularly on the cathodic side.^95^ Repeated switching between oxidising and reducing potentials leads Pt to shift between metallic and oxidised states, which can lead to dissolution and changes in surface structure over time.^96^ Carbon support degradation is another common reason for the decrease in the performance of fuel cells.^97^ In order to design catalysts for fuel cells with high activity and stability, it is crucial to study the degradation of the catalysts under relevant conditions to gain a deep understanding of the degradation mechanism. To obtain more quantitative information on the degradation process, the IL-EM can be used to provide valuable insights into changes in the sample over time.

To understand the degradation of commercial Pt catalysts, square wave voltage ASTs were performed on single-cell proton exchange membrane fuel cells (PEMFCs) with a Pt-catalyst membrane electrode assembly to mimic relevant reaction conditions.^98^ IL-SEM (Fig. 18a) was used to monitor the evolution of the cathodic catalytic layer (CL) over 14 000 AST cycles in the potential range of 0.6 to 1.0 V *vs.* RHE. It was revealed that Ostwald ripening, crystallite migration, and coalescence processes cause the degradation of Platinum nanoparticles. Specifically, smaller Pt nanoparticles (<5 nm) merge with larger Pt nanoparticles (>5 nm) or dissolve and redeposit into more stable nanoparticles, thus increasing the average particle size by about 35% during the ASTs. Some Pt nanoparticles increased in size during the initial AST and then decreased in size during the subsequent AST process. In this case, deformation of the carbon support was also observed, with some carbon particles moving. However, the main degradation mechanism appeared to be agglomeration and dissolution of Pt nanoparticles. The IL-SEM data were correlated with the ECSA, which decreased by about 72% due to changes in the catalyst. Even though IL-SEM measurements have been helpful in following the local degradation of the CL in PEMFCs under operational conditions, they cannot provide a complete picture of the degradation mechanisms due to the spatial resolution of SEM of about 3 nm, which means that nanoparticles and nanoclusters smaller than this size, and single atoms of Pt, remain undetected in the proposed mechanism.

**Fig. 18:**
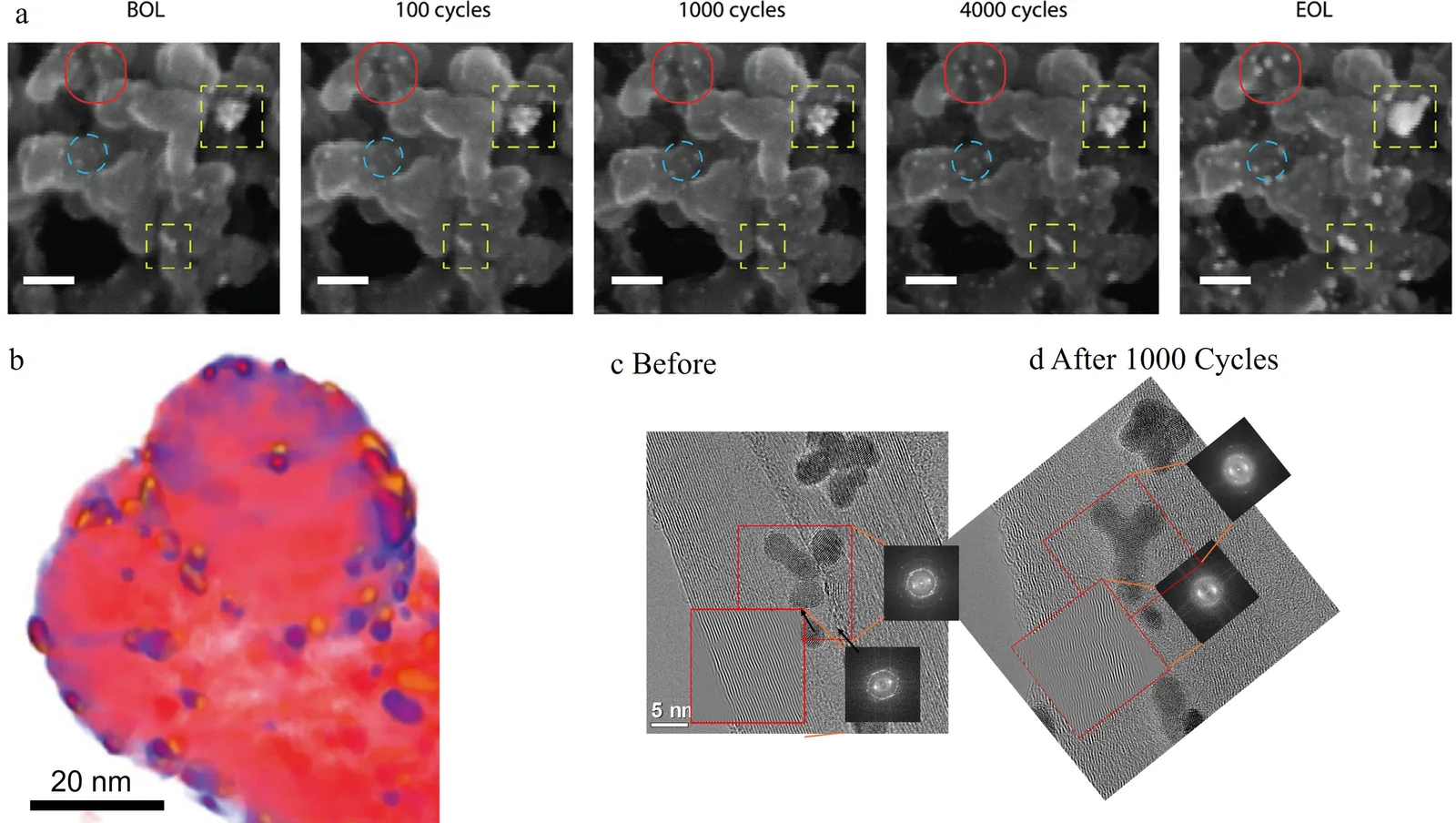
(a) IL-SEM images of the cathodic catalytic layer highlighting different types of catalyst changes. Scale bar is 50 nm. Reproduced from ref. 98 with permission from the American Chemical Society, copyright 2022. (b) Overlay of 3D perspectives of Pt NPs on a carbon support before and after 6000 potential cycles. Before cycling, the particles and the carbon support are represented in yellow and red, respectively, whereas after 6000 cycles, the particles and the carbon support are represented in purple and blue, respectively. Adapted from ref. 99 with a figure provided by Paulo J. Ferreira. Aberration-corrected TEM images of the Pt NPs on CNTs (c) before potential cycling and (d) after 1000 potential cycles. Adapted from ref. 102 with a figure provided by Paulo J. Ferreira.

Platinum nanoparticles (∼2.0 nm) supported on Vulcan XC72 carbon (5 wt% loading) were subjected to IL-TEM during potential cycling between 0.6 and 0.95 V *vs.* RHE at a sweep rate of 0.5 V s^−1^ in 0.1 M HClO_4_ under N_2_, with observations conducted from 1000 to 10 000 cycles and complementary tomography acquired before cycling and after 6000 cycles.^99^ Two-dimensional IL-TEM images tracked particle migration, coalescence, and the appearance and disappearance of Pt nanoparticles, alongside apparent changes in the carbon support; however, projection effects arising from the three-dimensional morphology of the support limited the interpretation of interparticle distances and support evolution. Three-dimensional IL-TEM reconstruction (Fig. 18b) resolved these ambiguities, showing that coalescence occurred between particles initially separated by less than 0.5 nm, while additional Pt nanoparticles emerged on the carbon surface following dissolution and subsequent cluster growth *via* Ostwald ripening, accompanied by changes in particle morphology. Reconstruction of the support structure indicated that contrast variations observed in 2D images corresponded to structural rotation of carbon rather than unambiguous carbon loss, highlighting discrepancies between projected and volumetric observations and their impact on interpreting degradation pathways.

The Pt nanoparticle catalysts modified with an aromatic alkylamine (unwashed catalyst) not only exhibit ORR activity but also show high durability compared to a commercially available catalyst.^100^ IL-TEM was used to understand the mechanism of improved durability, where the accelerated durability test was conducted through triangle wave cycling between 1.0–1.5 V *vs.* RHE at a scan rate of 500 mV s^−1^ in an Ar-saturated solution of 0.1 M HClO_4_. Under these conditions, the Au TEM grid was shown to be unsuitable for IL-TEM because the redox processes of Au occur at potentials within the applied potential window. To avoid this problem, the Au TEM grid was coated with iridium oxide, which prevented the dissolution and redeposition of Au under start-up/shut-down (SUSD) conditions. A Pt nanoparticle catalyst with an average size of 2.6 nm on carbon black coated with pyrene-containing amine molecules, as prepared or with butylamine washing (washed catalyst), was prepared for comparison purposes. After 1000 potential cycles, the average nanoparticle size of the as-prepared catalyst increased from 2.3 nm to 2.5 nm, while that of the washed catalyst increased from 2.8 nm to 3.7 nm. This shows that Pt nanoparticle catalysts modified with the pyrene-containing amine molecules are more stable under the test conditions. The ECSA behaviours of the washed and unwashed (as-prepared) catalysts were also different, increasing up to 1000 cycles for the latter and up to 500 cycles for the former. The CV of the washed catalyst showed an increase in the electric double layer of 0.4–0.6 V with increasing potential cycles, indicating carbon corrosion. The electrochemical evidence, combined with IL-TEM, in this study allowed the authors to conclude that aromatic groups on the surface of Pt nanoparticles hinder carbon corrosion and the migration and coalescence of the nanoparticles, as revealed by IL-TEM.

Platinum nanoparticles with an initial size of approximately 2.7–3.0 nm supported on hollow graphitic spheres (Pt@HGS) have been investigated under AST consisting of 1000 CVs between 0.05–1.4 V *vs.* RHE at 100 mV s^−1^ in 0.1 M HClO_4_ (pH 1), using both single-entity electrochemistry on a carbon nanoelectrode and ensemble IL-STEM measurements in Ar- and O_2_-saturated electrolytes, as well as in a diluted electrolyte at pH 4 (10^−4^ M HClO_4_ + 0.0999 M LiClO_4_).^101^ IL-STEM of the single-entity configuration revealed the disappearance of a substantial fraction of Pt nanoparticles accompanied by pronounced coarsening, with the remaining particles reaching an average diameter of 6.1 ± 2.2 nm, consistent with dissolution and subsequent redeposition under conditions of enhanced mass transport. In contrast, IL-STEM performed on particles deposited on TEM grids showed markedly reduced degradation, with structural changes largely confined to the outer surface of the Pt@HGS particles. Under Ar-saturated conditions, significant particle loss was observed without measurable size increase (2.8 ± 0.9 nm to 2.9 ± 1.2 nm), whereas in O_2_-saturated electrolyte, the particles were largely retained and exhibited moderate growth from 3.0 ± 0.9 nm to 3.5 ± 1.0 nm, accompanied by a reduction in particle number of approximately 32%. When the electrolyte pH was increased to 4, IL-STEM indicated minimal structural evolution, with particle sizes remaining close to the as-prepared distribution (∼2.94 ± 1.79 nm). These variations in structural evolution correlate with differences in degradation behaviour under ORR conditions, where locally increased pH values are associated with reduced Pt dissolution and enhanced redeposition.

Platinum nanoparticles with an average size of approximately 3.2 nm supported on carbon nanotubes and carbon black were examined using IL-AC-TEM under half-cell AST involving potential cycling between 1.0 and 1.5 V *vs.* RHE at 0.5 V s^−1^ in N_2_-saturated 0.1 M HClO_4_ for up to 5000 cycles.^102^ IL-TEM (Fig. 18c and d) after 1000 cycles revealed that particle migration followed by coalescence dominated the early-stage evolution, with initially separate nanoparticles moving and merging into larger single-crystal particles, while the carbon nanotube (CNT) support underwent structural modification with basal planes transforming into wavy-like morphologies. At approximately 1500 cycles, IL-TEM imaging showed the emergence of sub-nanometer clusters and single atoms on the CNT surface, consistent with Pt dissolution, accompanied by an increased separation between larger particles. Upon further cycling to 2000 and 5000 cycles, continued particle migration and coalescence were observed, along with Ostwald ripening, in which smaller species disappeared and contributed to the growth of larger particles. Comparable IL-TEM observations for Pt nanoparticles supported on carbon black indicated similar processes, including particle mobility, coalescence, and partial or complete dissolution during cycling. These structural changes correlate with a reduction in ECSA, which was associated with particle migration and coalescence during the initial stages of degradation.

The cathodic catalytic Pt/C electrode layer of a commercial three-layer catalyst-coated membrane (CCM, IRD Fuel Cells, Pt loading: 0.1/0.4 mg_Pt_ cm^−2^) in a PEMFC under operation was investigated by performing IL-SEM and IL-TEM.^103^ AST was performed to mimic the SUSD process, where the cell voltage (two-electrode configuration) was cycled between 1.0 and 1.5 V at a scan rate of 500 mV s^−1^, for a total of 10 000 cycles. IL-SEM on micro- and macroscopic scales has observed propagation of cracks in carbon film and delamination of carbon layer from the supporting film, which contributed to a 2–4 fold increase in the cell electrical resistance and a loss of 79% initial activity. While IL-TEM (Fig. 19a) on the nanoscopic scale showed that electrochemical corrosion of the carbon support causes shrinkage of carbon structures by ∼20%, leading to Pt nanoparticles agglomerating and growing. The mean size of Pt nanoparticles increased from *ca.* 3.5 nm to above 5 nm after 10 000 cycles, where the 63% growth of Pt nanoparticles correlated with a 65% decrease in ECSA. Here, the structural information is based on the averaging IL information for 500 Pt nanoparticles, which gives strong statistics but loses information about single-particle evolution, which could explain the atomistic mechanisms responsible for the restructuring of Pt nanoparticles. Quicker corrosion of carbon was revealed where more Pt nanoparticles are present, indicating that carbon corrosion may be catalysed by Pt.

**Fig. 19:**
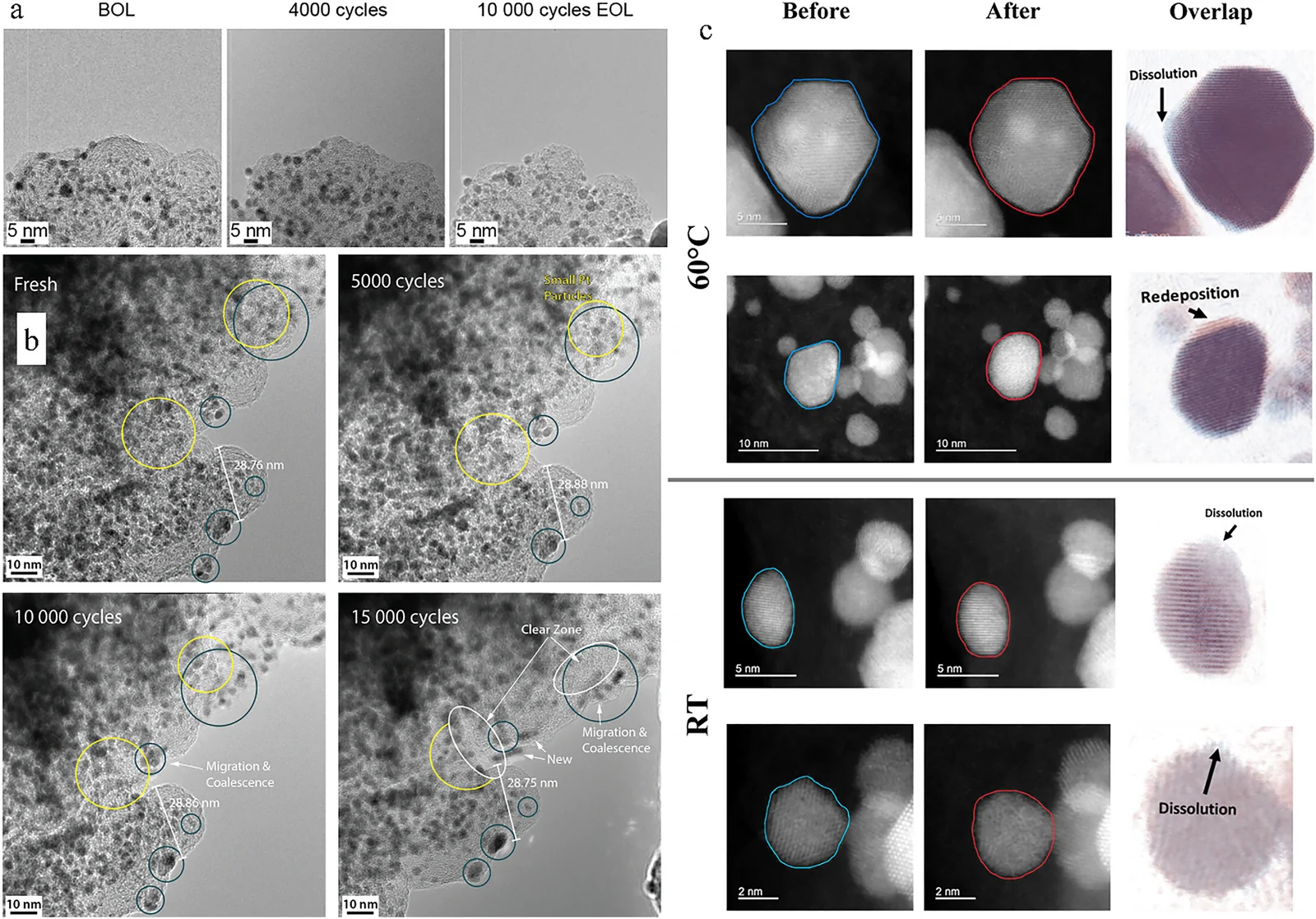
(a) IL-TEM images on the edge to focus on the surface (representative average region on the CL), at (left) BOL, (middle) after 4000, and (right) after 10 000 SUSD cycles. Reproduced from ref. 103 with permission from the American Chemical Society, copyright 2024. (b) IL-TEM images for the fresh fuel cell and after 5000, 10 000 and 15 000 normal operation AST potential cycles.^104^ with permission from the Royal Society of Chemistry, copyright 2023. (c) IL-STEM images of the Umicore Pt–Co/C sample depicting dissolution and redeposition influenced by surface energy after the ADT at 60 °C and room temperature. Reproduced from ref. 106 with permission from the American Chemical Society, copyright 2025.

Two types of ASTs were implemented to study Pt/C catalysts derived from a commercial membrane electrode assembly in a real PEMFC device.^104^ The first type of AST is used for simulating normal operation, where the sample is cycled between 0.6 and 1.0 V *vs.* RHE at 16 s per cycle up to 15 000 cycles. Lacey carbon Au grids were used for normal operation AST. Under normal operation conditions, Pt nanoparticles (Fig. 19b) were found to grow through Ostwald ripening, migration and coalescence, while the carbon support was relatively stable. AST also showed that both the current and the hydrogen underpotential deposition (*H*_upd_) region in the CVs decrease during the potential cycling, which corresponds to the decrease in ECSA. The second type of AST was used for simulating SUSD, where the sample was cycled between 1.0 and 1.5 V *vs.* RHE at 500 mV s^−1^ up to 10 000 cycles. An Au TEM grid without a carbon film has been used for SUSD AST to avoid the influence of the carbon film on catalyst degradation. Under SUSD conditions, the carbon support has been found to degrade through loss of volume and collapse, which forced the Pt nanoparticles closer to each other and to grow. The changes in the catalyst led to a decrease in fuel cell performance and a 68% loss in ECSA. When a TEM grid with a non-conductive layer (Formvar) was used in the measurement, no obvious degradation of the catalyst was found even after 15 000 potential cycles. This proved that a good electrical contact between Pt/C and the TEM grid is essential for successfully studying the catalyst degradation process.

Nanoparticles of PtCo (4–5 nm) supported on high-surface-area carbon (HSC) and Pt/HSC (2–3 nm) were examined under potential cycling in Ar-purged H_2_SO_4_ (0.05 or 0.5 M) at 80 °C between 0.6 and 0.95 V *vs.* RHE for 30 000 cycles, with additional tests in 0.57 M HClO_4_ at room temperature and extended potential windows up to 0.40 to 1.05 V.^105^ IL-HAADF-STEM combined with SE imaging, the latter probing particles within ∼4–5 nm of the carbon surface, revealed pronounced particle growth for Pt/HSC, whereas PtCo/HSC exhibited minimal positional displacement and no measurable shift in particle size distribution under identical conditions. In contrast, membrane electrode assembly measurements showed an increase in PtCo particle size from 4.3 to 5.1 nm after 30 000 cycles, despite comparable Co loss (∼6 at%) detected by EDS in both environments. Variations in temperature, electrolyte type (H_2_SO_4_*vs.* HClO_4_), and acid concentration did not alter the particle size distribution of PtCo/HSC in IL-STEM experiments. When the potential window was extended to 0.4–1.0 V in a Pt-ion-containing electrolyte, a reduction in the fraction of smaller particles, growth of larger particles to a median size of ∼4.7 nm, and a shift toward more spherical morphologies were observed, consistent with an increased contribution from Ostwald ripening.

Commercial Pt–Co/C electrocatalyst nanoparticles (Umicore Elyst Pt50 0690) deposited on a gold TEM grid were investigated by IL-STEM before and after accelerated degradation testing (ADT) in 0.1 M HClO_4_, involving potential cycling between 0.6 and 0.95 V *vs.* RHE for 10 000 cycles at room temperature and 60 °C.^106^ At the single-particle level, IL-STEM (Fig. 19c–h) images acquired after cycling revealed a progressive loss of faceting, with sharp edges and corners disappearing and particles adopting more rounded morphologies. At higher temperatures, additional structural evolution was observed at the ensemble level, including the formation of necks between neighbouring particles and subsequent coalescence into larger entities, whereas at room temperature, such processes were not detected and changes were limited to more subtle reshaping. These morphological changes were accompanied by modifications in electrochemical properties, with CO electrooxidation measurements showing a shift of the oxidation peak toward more negative potentials and a decrease in the ECSA from ∼97% retention after ADT at room temperature to ∼88% at 60 °C. The increase in particle size dispersion following cycling, consistent with IL-STEM observations of coalescence, coincided with reductions in both specific and mass activity of the catalyst.

The electrochemical stability of rhodium–platinum core–shell nanoparticles on a carbon support (Rh@Pt/C nanoparticles, 5.9 nm) was explored using IL-STEM (Fig. 20a–d) to track the morphological and compositional evolutions of Rh@Pt/C nanoparticles during 10 000 cycles (0.06–0.80 V *vs.* RHE, 0.5 M H_2_SO_4_).^107^ The as-prepared Rh@Pt/C nanoparticles showed that the Rh core is completely covered by the Pt shell. After 10 000 cycles, both dissolution and redeposition of the outermost surface atomic columns of Pt were found. However, the size and composition of Rh@Pt/C nanoparticles remained nearly constant under potential cycles, whereas no particle aggregation, Ostwald ripening, or progressive catalysis dissolution was revealed by the IL-STEM observation. It was found that particle detachment from the carbon support is the main reason for the decrease in electrochemical current in Rh@Pt/C nanoparticles during potential cycling. The results showed high stability of Rh@Pt/C and revealed the main degradation mechanism: particle detachment from the carbon support. Therefore, the perspective of increasing stability should focus on strengthening the particle-carbon support interaction or developing new support materials.

**Fig. 20:**
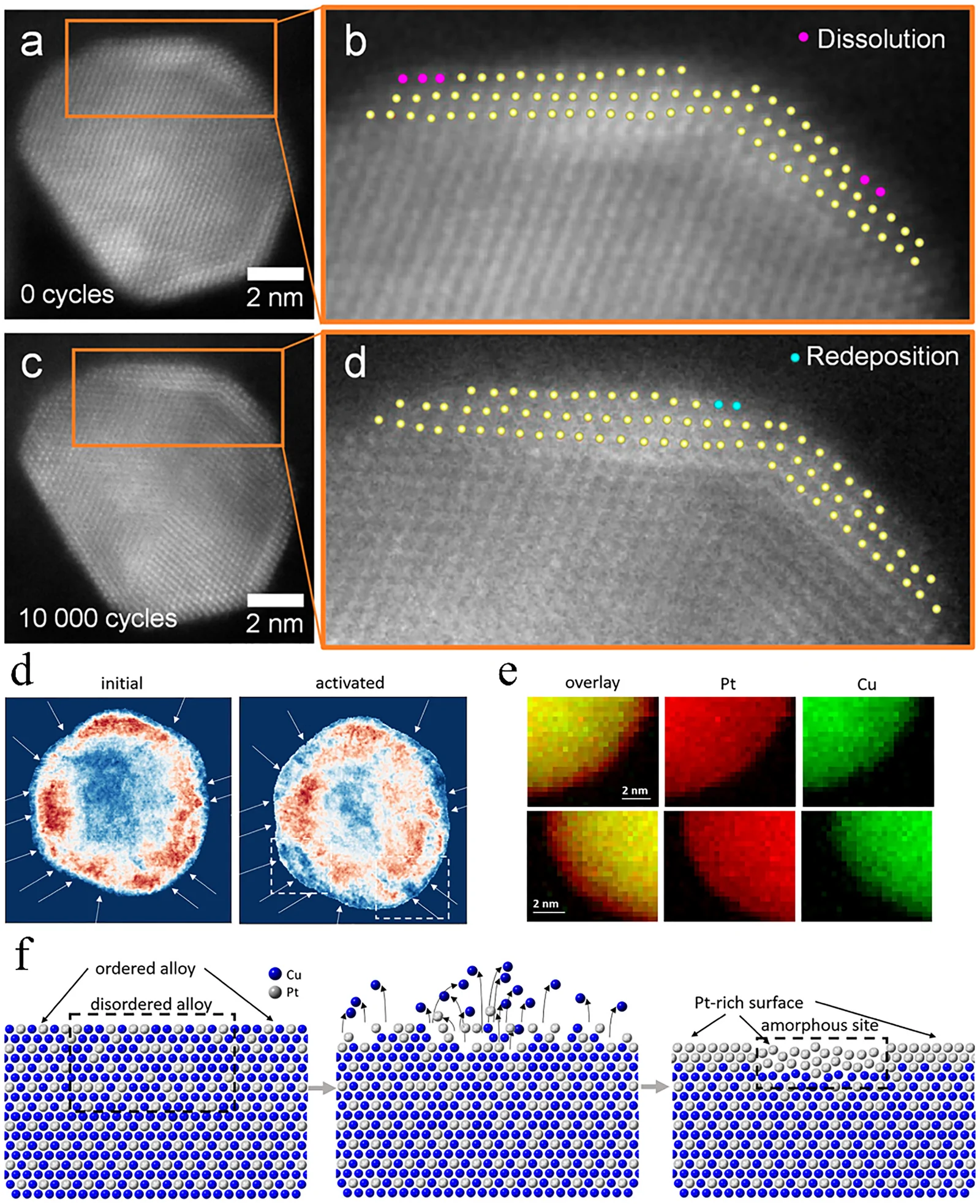
IL-STEM images of Rh@Pt/C NPs before (a) and (b) and after (c) and (d) 10 000 cycles. Reproduced from ref. 107 with permission from the Royal Society of Chemistry, copyright 2023. (d) Maps depicting alloy ordering within a Pt–Cu nanoparticle after synthesis (left) and after potential cycling activation (right). White arrows denote sites with observed local amorphisation after activation. (e) EELS elemental maps for two parts of a nanoparticle after activation. (f) A schematic depiction of the proposed mechanism for the local amorphisation on the Pt–Cu nanoparticle surface. Reproduced from ref. 109 with permission from the American Chemical Society, copyright 2025.

Alloying Pt with Ni has emerged as an effective approach for ORR and OER catalysts in fuel cells.^108^ In this study, sub-10 nm PtNi octahedra, employed as a bifunctional electrocatalyst, exhibited reduced Pt dissolution and hence improved catalyst durability. The real-condition test has been carried out in unitised regenerative fuel cells (URFC) in the order of ORR, OER, and recovery CV for one cycle. The IL-TEM before and after the URFC did not show significant morphological changes or degradation of PtNi octahedra (however, no information is given on the specifics of the IL-TEM conditions), with Pt mostly located at the edges and Ni on the facets. This proved that the alloying effect of PtNi hinders the dissolution of Pt and facilitates the transformation of PtO_*x*_ to metallic Pt *via* a recovery process. The performance of the anion exchange membrane (AEM)-based URFC single cell was stable over 10 cycles after the recovery process, demonstrating long-term operation.

Carbon-supported Pt–Cu particles with a broad size distribution (4–100 nm) were examined by identical-location HAADF-STEM and 4D-STEM before and after activation treatment consisting of acid washing in 0.1 M HClO_4_ for 30 s followed by 200 potential cycles between 0.05 and 1.20 V *vs.* RHE at 300 mV s^−1^ in 0.1 M HClO_4_ using a modified floating electrode setup.^109^ After acid washing, IL-HAADF-STEM imaging showed only minor particle reshaping, while IL-EDS indicated enrichment of platinum at the surface, together with copper removal from the outermost layers. Following potential cycling, the particles transitioned from initially faceted morphologies to more irregular, rugged surfaces, accompanied by a reduction in particle size of approximately 3.6%. IL-4D-STEM analysis (Fig. 20d–f) further revealed the disappearance of ordered alloy domains at the particle surface and the emergence of localised amorphous regions, indicating a loss of crystallinity at specific surface sites. These structural changes were consistent with electrochemical measurements, in which cyclic voltammograms in the final cycle showed features characteristic of platinum, indicating the formation of a Pt-rich surface, alongside a modified surface associated with an increased ECSA.

### 3.9 Electrocatalysis of organic reactions

A sphere of CoNiFeCu alloy, approximately 500 nm in diameter, was attached to a focused ion beam-processed nanoelectrode and studied for its electrocatalytic oxidation of solketal (SOR), a derivative of glycerol, in 1 M KOH.^110^ This study involved up to eight CV scans, ranging from 1.3 to 1.75 V *vs.* RHE. A control experiment was conducted under the same conditions but without solketal, operating instead under OER catalysis conditions. IL-STEM, combined with IL-EDS elemental mapping of oxygen, revealed that the activation of the catalyst in the presence of solketal does not require the formation of the oxyhydroxide phase (MOOH), which typically emerges under OER conditions. Notably, the IL-EDS mapping of copper showed a reduced extent of copper leaching (Fig. 21a and b) from the catalyst when solketal was present. This observation supports the conclusion that solketal protects the catalyst from hydroxide ion attack, thus inhibiting the formation of MOOH. Furthermore, a significant increase in current density was observed in CV when solketal was present compared to the control without it, and *operando* attenuated total reflectance Fourier-transform infrared (ATR-FTIR) spectroscopy was also performed, identifying the products formed during the reaction.

**Fig. 21:**
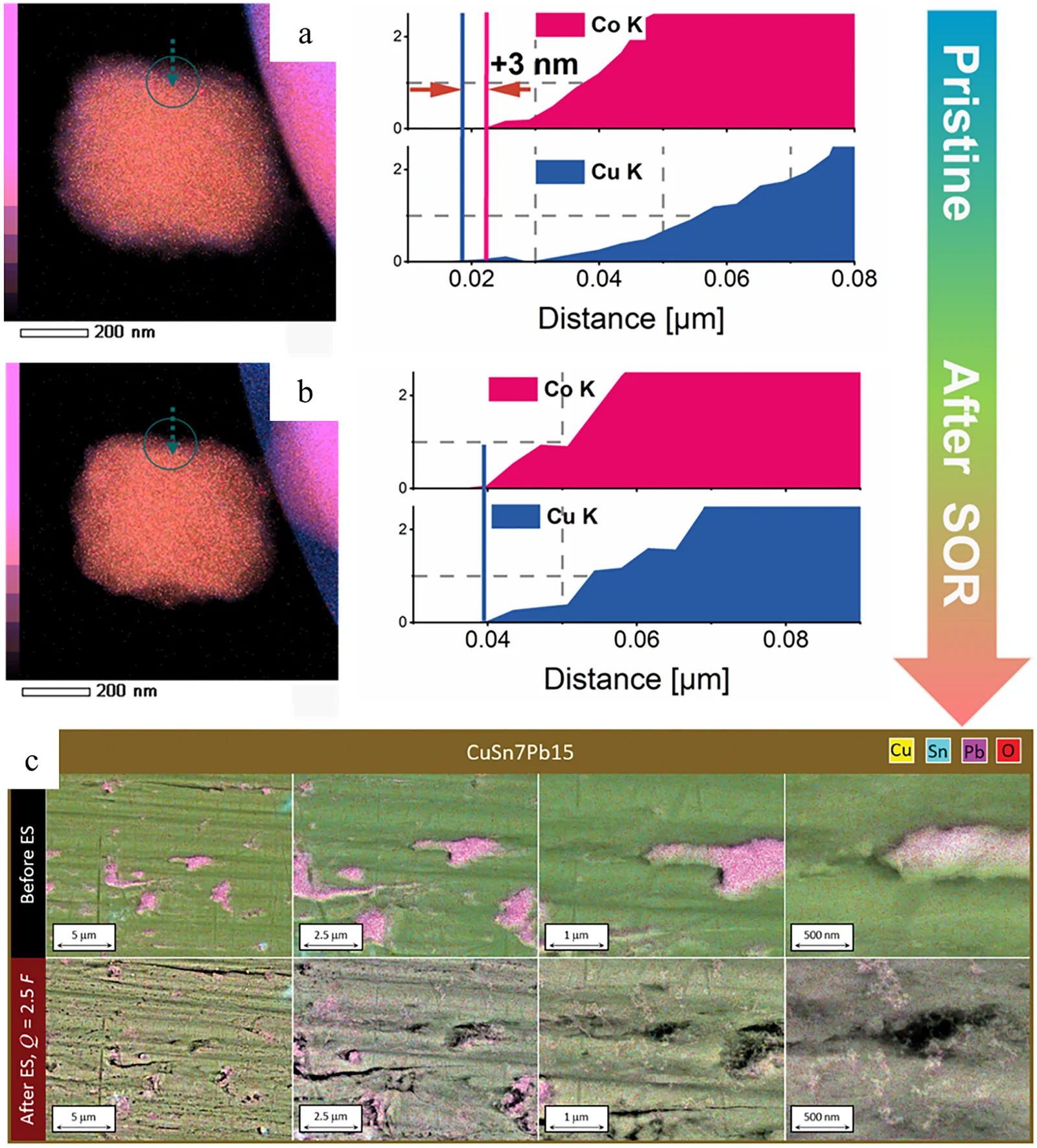
IL-EDS mapping images and corresponding parallel line scan curves of CNE_CoNiFeCu (1 : 1 : 1 : 0.5) nano-assemblies before (a) and after (b) 8 CV cycles SOR. Reproduced from ref. 110 with permission from John Wiley and Sons, copyright 2025. (c) IL-EDS investigations of CuSn_7_Pb_15_ before and after electroreductions (*j* = −10 mA cm^−2^, *Q* = 2.5 *F* and *ω* = 400 rpm). Reproduced from ref. 111 with permission from John Wiley and Sons, copyright 2023.

The behaviour of the foam-like catalyst Cu_21_Pb_61_O_18_, prepared using the dynamic hydrogen bubble template (DHBT) deposition method, was studied for the electroreduction of 2,6-dichlorobenzaldoxime to 2,6-dichlorobenzonitrile under galvanostatic conditions at a current density of *j* = −10 mA cm^−2^, with a total charge of *Q* = 2.5 F and a cell potential of approximately −3 V.^111^ The performance of this catalyst was compared to that of the CuSn_7_Pb_15_ alloy. IL-SEM imaging and IL-EDS elemental mapping were employed both before and after the reaction (Fig. 21c). This revealed a complex three-dimensional morphology of the Cu_21_Pb_61_O_18_ catalyst, characterised by open pores and different phases present as particles, clusters, and plates at both micron and sub-micron scales. After the reaction, the IL-SEM images showed only minor morphological changes on the microscale. Specifically, highly crystalline hexagonal b-PbO and CuSn particles remained virtually unchanged, while PbSn particles experienced mild agglomeration. In contrast, the CuSn_7_Pb_15_ alloy, which contains Pb in a metallic form, exhibited significant changes in regions containing Pb. The microscopy results were correlated with ICP-MS analysis of the solution: low leaching of Pb corresponded to minor morphological changes observed in the IL-SEM, indicating the importance of maintaining the purity of the synthesised product from Pb contamination.

### 3.10 Battery-type electrochemical transformation

Two-dimensional antimonene flakes with micrometre-scale lateral dimensions synthesised from SbCl_3_ were investigated by IL-S/TEM after electrochemical cycling.^112^ The flakes, dispersed on a Cu TEM grid, were incorporated into coin cells and cycled five times at 0.1 A g^−1^ in 1 M potassium bis(fluorosulfonyl)imide/tris(2,2,2-trifluoroethyl) phosphate (KFSI/TEP) electrolytes, followed by retrieval and re-examination of identical regions. IL-S/TEM imaging showed that the flakes retained their overall structural integrity without fracture, while their lateral dimensions decreased slightly (*e.g.*, 0.77 to 0.72 µm in TEP) and a wrinkled morphology developed across the 2D layers. The corresponding SAED patterns changed from discrete diffraction spots to halo features, indicating a transition from a single-crystalline to an amorphous state after cycling. HRTEM images revealed the formation of a thin amorphous surface layer with a thickness of ∼3.1 nm in the TEP-based electrolyte and ∼6.7 nm in the tris­(2,2,2-trifluoroethyl) phosphate (TFP)-based electrolyte. EELS mapping detected K, P, F, and S species distributed at the flake surface, consistent with electrolyte decomposition products. These structural and interfacial changes were analysed alongside XPS and Raman measurements and correlated with the stable cycling performance of the corresponding potassium-ion cells.

### 3.11 Hydrogen and methanol oxidation reactions

Palladium nanoparticles with an average size of ∼2.3 nm supported on carbon were further modified by atomic layer deposition to introduce a TiO_2_ overlayer, forming a Pd@TiO_2_/C architecture.^113^ The catalyst deposited on TEM grids was subjected to accelerated durability testing by cycling between 0 and 0.4 V *vs.* RHE for up to 7000 cycles in H_2_-saturated 0.1 M KOH. IL-TEM revealed pronounced structural evolution in Pd/C, with particle growth *via* coalescence and Ostwald ripening, the disappearance of enlarged particles indicative of detachment, and the progressive loss of smaller particles consistent with dissolution. In contrast, Pd@TiO_2_/C exhibited largely unchanged particle morphology and distribution over the same cycling protocol, with only minor local variations. This stabilisation correlates with electrochemical measurements showing a smaller increase in overpotential after durability testing, and *operando* XAS further links the preserved structure to interfacial electronic interactions between Pd and TiO_2_ under working conditions.

Pd nanocubes (∼18 nm) with dominant (100) facets, supported on carbon, were examined, alongside Pd@Pt counterparts bearing an ultrathin Pt overlayer, under methanol oxidation conditions in 1.0 M KOH + 1.0 M CH_3_OH electrolyte, using a reference-indexed identical-location TEM configuration at 0.8–1.0 V *vs.* RHE over 10 h.^114^ IL-TEM tracking at 2 h intervals showed that the nanocubes remained anchored to the support, while neighbouring particles progressively approached and coalesced, forming interconnected structures at extended times. High-resolution analysis after electrochemical operation revealed lattice distortion and newly formed interfacial structures within fused particles, consistent with dissolution and reconstruction processes. *In situ* Raman measurements detected surface-bound –OH intermediates during operation, and their presence coincided with the observed morphological evolution and loss of electrochemically active surface area. Introducing a Pt overlayer preserved the cubic morphology and suppressed the extent of particle coalescence, accompanied by the absence of detectable –OH vibrational features and improved retention of catalytic activity.

PtRu nanoparticles with sizes of 3–4 nm, synthesised by citric acid-assisted thermal reduction and encapsulated within nitrogen-doped carbon shells on a carbon support, were evaluated under accelerated stress testing in H_2_-saturated 0.1 M HClO_4_ by cycling between 0.000 and 0.800 V *vs.* RHE for 5000 cycles.^115^ IL-TEM imaging before and after cycling showed no detectable variation in particle size, spatial distribution, or morphology across the analysed regions. The nanoparticles remained uniformly dispersed, with no evidence of coalescence, ripening, or material loss. This structural invariance is consistent with electrochemical measurements, including hydrogen oxidation reaction polarisation and *in situ* FTIR spectroscopy, which indicated retention of catalytic activity and resistance to poisoning under the same conditions.

## 4 Light-induced structural evolution probed by IL-EM

Photocatalysis is an emerging technology that has the potential to harness light energy to drive chemical transformations, enabling cleaner and more efficient processes. Understanding the structural and compositional changes in photocatalysts and other photoactive materials is essential for comprehending the chemical reactions promoted by light. Currently, there are few examples of using identical-location microscopy to study photocatalysts. One such example involves WO_3_ nanoparticles, which are less than 100 nm in size, being irradiated in air with UV light at wavelengths of 254 nm and 185 nm for 4 h.^116^ IL-STEM imaging showed no changes in particle morphology or atomic structure; however, it did detect the formation of an amorphous sub-nanometre layer on the surfaces of the nanoparticles (Fig. 22). The microscopy data were correlated with XPS measurements on bulk material, which revealed the formation of C=O and C–OH groups due to the photooxidation of hydrocarbons during UV light irradiation. It was suggested that the origin of these hydrocarbons was contamination from the air.

**Fig. 22:**
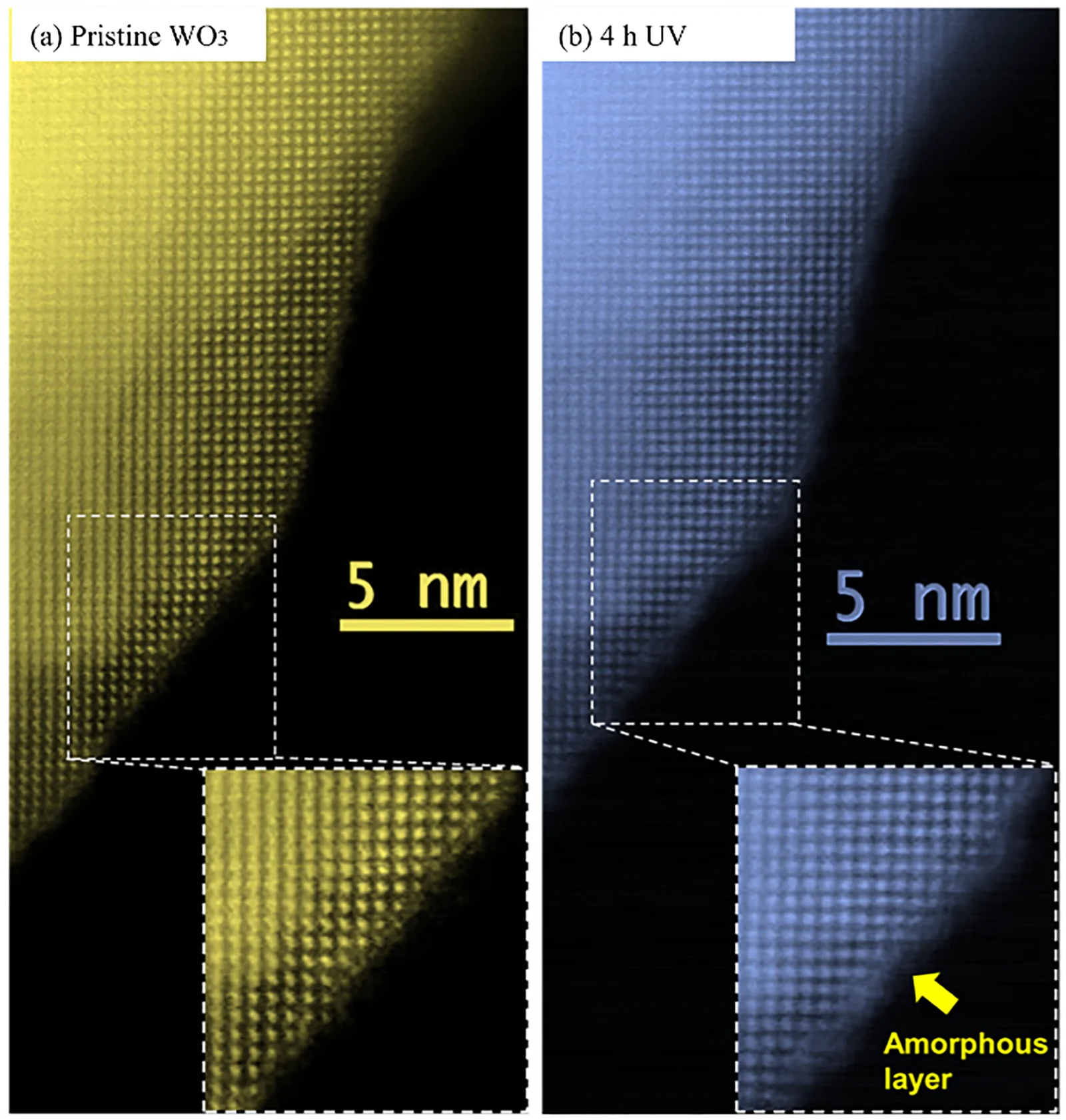
Atomic-scale HAADF-STEM images of WO_3_ particles before and after UV irradiation. Reproduced from ref. 116 with permission from MDPI, copyright 2024.

## 5 Thermally activated processes

### 5.1 Ammonia formation and decomposition

As most industrial catalytic reactions rely on heat, it is crucial to understand the thermally activated evolution of a catalyst. These processes can be diverse and complex, and can be divided into three main stages: catalyst activation, steady state, and deactivation. Understanding the atomic-level changes occurring in the catalyst at each stage is important. For instance, Wüstite (FeO) can serve as an effective pre-catalyst in the ammonia synthesis reaction. The activation of Wüstite is accomplished by heating the material in a hydrogen/argon atmosphere at 10 bar. IL-TEM of a few hundred nanometer-wide disk-like Wüstite particles heated at 365 °C under these conditions reveals extensive surface cracking along with the development of protrusions (Fig. 23a and b).^117^ A close examination of HRTEM images confirms the presence of nanocrystalline α-Fe and magnetite (Fe_3_O_4_) phases. This indicates that both reduction and disproportionation of Wüstite are occurring, although the process is incomplete, as the Wüstite phase is still present. When the temperature is increased to 470 °C, the reduction to polycrystalline α-Fe is completed. The changes observed in IL-TEM are consistent with bulk measurements obtained through XRD, demonstrating how IL-TEM can be effectively employed to examine catalyst activation processes with nanoscale precision.

**Fig. 23:**
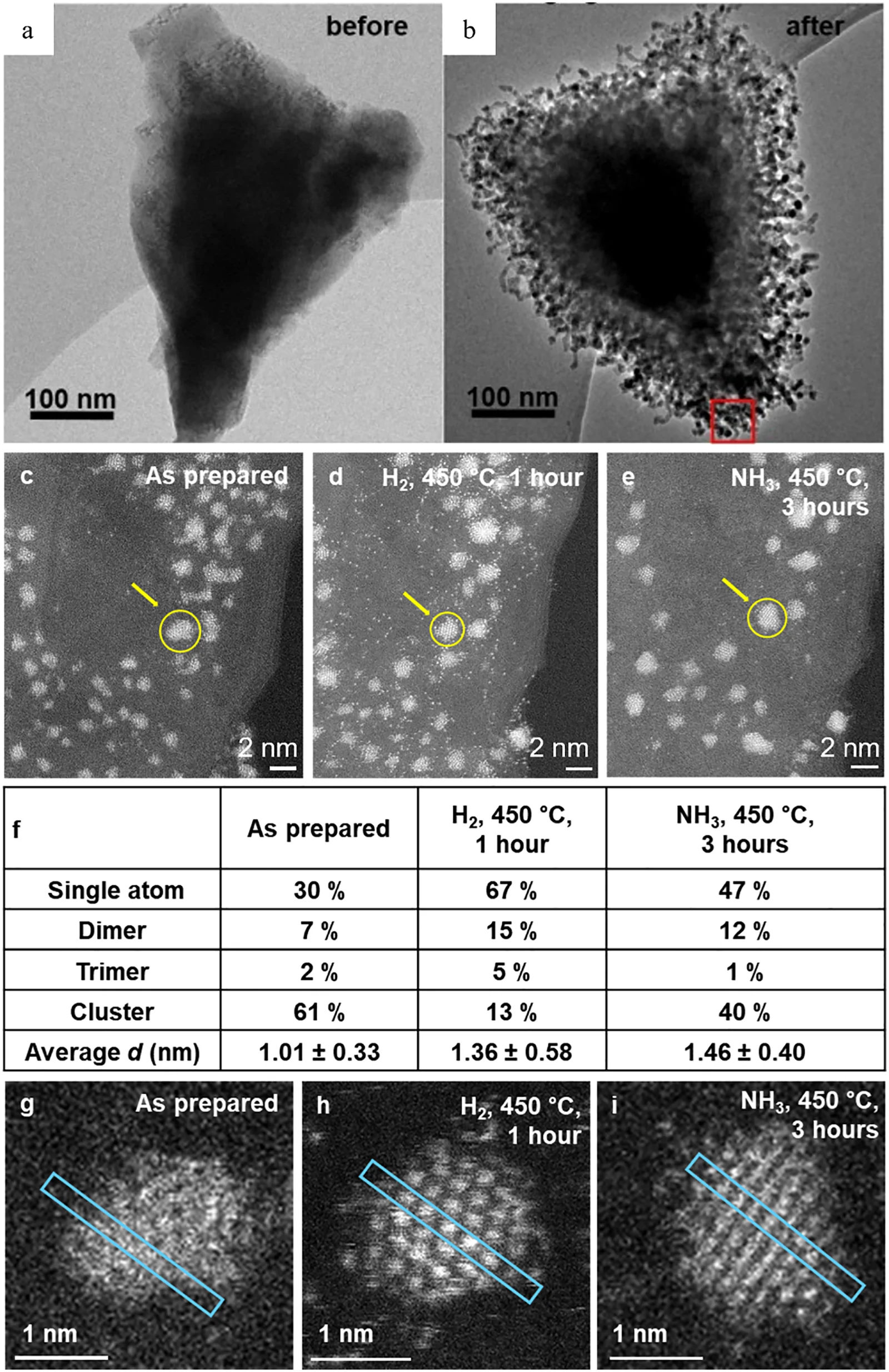
IL-TEM observations of FeO before (a) and after (b) the experiment (H_2_/Ar = 3/1, 10 bar, 365 °C). Reproduced from ref. 117 with permission from Elsevier, copyright 2022. IL-STEM images of Ru/GNF at different stages: as-prepared (c), after 450 °C in H_2_ (d), and after 450 °C in NH_3_ (e) (scale bar, 2 nm). A tabulated summary of changes in the population of single atoms, dimers, trimers and nanoclusters, and the nanoclusters' average diameter, *d*, for each stage (f). An example of IL-STEM images for the evolution of a specific single Ru nanocluster (marked with the arrow in c–e) through different reaction stages (g)–(i). Reproduced from ref. 6 with permission from the Royal Society of Chemistry, copyright 2025.

Another significant reaction involving ammonia is the production of hydrogen through the thermal decomposition of NH_3_ into its elemental components. A key challenge in this process is enhancing the catalyst's performance to function effectively across a wide range of operating conditions while maintaining stability over extended periods. Recent advancements have seen the development of nanometre-sized Ru clusters, which are assembled directly from Ru atoms on the surface of graphitised carbon nanofibers.^6^ These nanoclusters have a substantial fraction of surface atoms but exhibit disordered structures. IL-STEM was utilised to monitor the evolution of the catalyst during the activation stage in hydrogen at 450 °C, followed by the reaction in 5% ammonia in argon at the same temperature for up to 12 h (Fig. 23c–i). IL-STEM imaging revealed that coalescence and Ostwald ripening were the primary mechanisms of nanocluster structuring and pyramidalization during the activation stage, with coalescence becoming the dominant mechanism under reaction conditions. Single Ru atoms formed during the activation process are absorbed by the nano-pyramids during the ammonia decomposition reaction. As a result, Ru nanoclusters evolve from an amorphous state to nano-pyramids featuring a hexagonal close-packed (hcp) lattice and a 2–3 nm^2^ footprint composed of 3–5 atomic layers. This configuration maximises the concentration of active sites essential for the rate-determining step in the reaction.

Consequently, IL-STEM provided insights that explained bulk kinetic observations, demonstrating that the activity of NH_3_ decomposition increased over 12 hours. A crucial aspect of this work is the quantitative analysis of STEM images, which involved counting the number of Ru atoms in individual particles and quantitatively assessing their changes and footprints. This method enabled the detection of subtle changes that might otherwise go unnoticed, highlighting the significance of nanocluster shape for catalyst activity. A comparison between ensemble-averaged (non-IL) data and IL analysis indicated that IL-STEM offers a more definitive understanding of the atomistic mechanisms governing nanocluster evolution. An important takeaway from this study is that the macroscopically measured activity of the heterogeneous catalyst cannot be explained solely by non-IL microscopy data. Instead, the IL information obtained from localised scale analysis can elucidate these phenomena without requiring extensive statistical data collection.

### 5.2 Oxidation of CO

The oxidation of carbon monoxide (CO) is commonly used as a model reaction for *in situ* investigations of catalysts. This is due to its straightforward mechanism and the valuable insights it offers into the chemical state of active sites, as observed through the behaviour of CO molecules. For example, in a study involving cobalt oxide (CoO) nanoparticles larger than 50 nm, the nanoparticles were heated on a TEM grid using an identical-location imaging and spectroscopy (ILIAS) setup in an environment of 2.3 bar of either O_2_ or N_2_ at temperatures of 150 or 250 °C.^118^ After pre-treating the catalyst in O_2_, the application of CO gas pulses resulted in the conversion of CO to CO_2_ at room temperature. To explain this phenomenon, IL-TEM was applied to show that the atomically flat surface of CoO changed to a terraced structure after heating at 250 °C in O_2_ (Fig. 24a and c). Additionally, if CoO was reduced prior to oxidative treatment, it underwent more significant transformations, becoming rough and featuring cube-like structures associated with the Co_3_O_4_ spinel phase. The imaging was augmented by IL-EELS, which confirmed an increased proportion of Co^3+^ after the oxidative treatment and visualised its distribution across the surface of individual catalyst nanoparticles (Fig. 24b and d). This nanoscale structural and chemical information aligns well with the macroscopically observed conversion of CO to CO_2_ at room temperature, indicating the presence of highly active oxidative species on the catalyst surface after heating in O_2_.

**Fig. 24:**
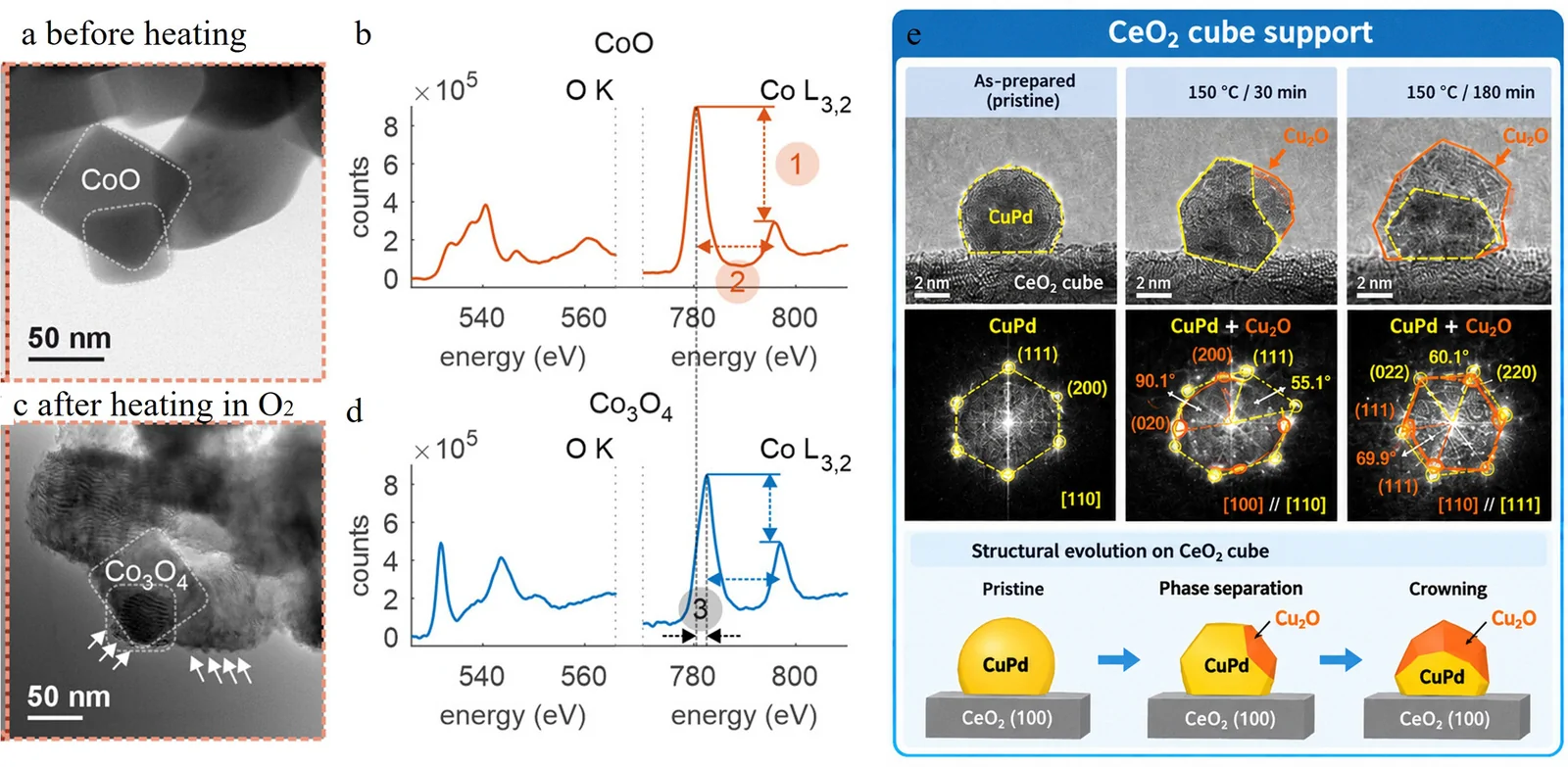
ILIAS before (a) and (b) and after (c) and (d) heating in O_2_. Reproduced from ref. 118 with permission from the American Chemical Society, copyright 2025. IL-TEM analysis of a single CuPd nanoparticle supported on the CeO_2_ cube (e). Adapted from ref. 119 with a figure provided by Langli Luo.

Bimetallic CuPd nanoparticles, approximately 7 nm in diameter, supported on cerium oxide, were studied using IL-TEM for the CO oxidation reaction at 150 °C over a total period of 180 min.^119^ Observations of individual nanoparticles were made at 0, 30, and 180 min throughout the activation process (Fig. 24e). Single-particle analysis revealed a significant change: the growth of a Cu_2_O phase that forms a shell around the original nanoparticle, while the core of the alloy becomes predominantly enriched with Pd. The phase separation behaviours of CuPd nanoparticles appear to be sensitive to the surface planes of the CeO_2_ support. Notably, when the catalyst operates below the critical temperature required for phase separation, the CuPd nanoparticles exhibit inactivity towards CO oxidation. However, after phase separation occurs, the CO conversion rate increases to nearly 100%. This indicates that the newly formed Cu_2_O/CuPd interface plays a crucial role in enhancing CO oxidation at lower temperatures.

### 5.3 Other heat-activated oxidation processes

The interface between catalyst particles and support materials is crucial, especially when nanoparticles are produced before being deposited onto the support. This is often achieved through colloidal synthesis, which requires stabilising nanoparticles with organic ligands. While these ligands are essential for controlling nanoparticle growth, they can negatively affect catalytic activity once nanoparticles are on the support. IL-TEM was utilised to investigate the removal of the stabilising ligand, 1-dodecanethiol, from 2 nm Au nanoparticles deposited on carbon supports.^120^ Treatments using O_2_/Ar at 300 °C for 1 h or H_2_/Ar at 400 °C for 1 h revealed that, while the average size of the nanoparticles increased as expected, smaller nanoparticles tended to disappear more frequently than larger ones. Similarly, IL-TEM results after 10 cycles between 0.05 V and 1.65 V *vs.* RHE in a 0.1 M KOH electrolyte also indicated an increase in particle size (Fig. 25a and b). Thermal gravimetric analysis-mass spectrometry (TGA-MS) was performed to confirm the removal of the alkyl-thiol layer from the nanoparticles during the annealing process. Importantly, CV measurements demonstrated an increase in the electrochemical activity of the Au nanoparticles post-treatment despite their larger size, highlighting the importance of eliminating the alkyl thiol molecules at the gold-carbon interface, as they create a barrier for electron transfer.

**Fig. 25:**
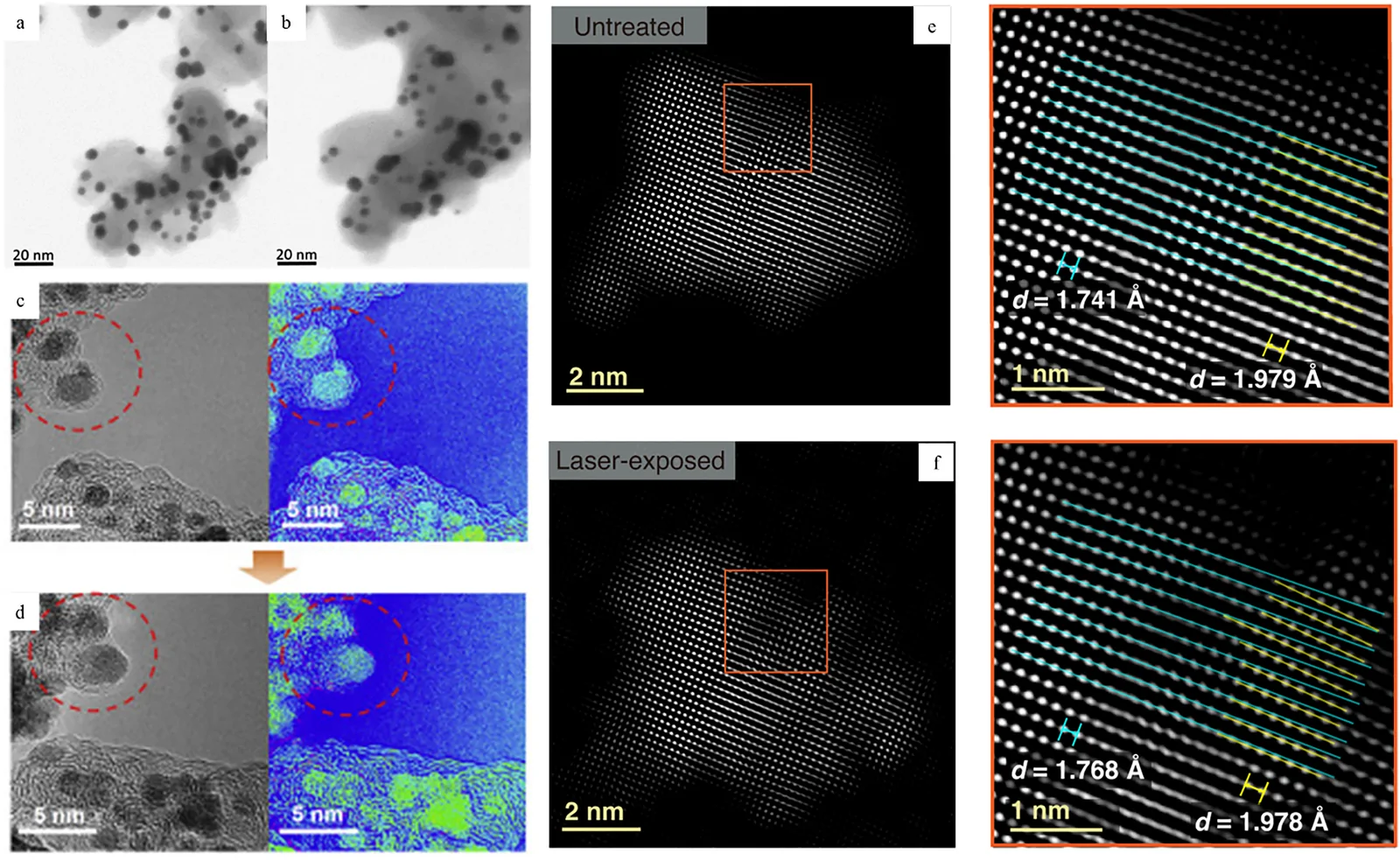
IL-TEM observation of O_2_ + H_2_-treated Au/Vulcan before (a) and after (b) being subjected to electrochemical stress in 0.1 M KOH. Reproduced from ref. 120 with permission from Royal Society of Chemistry, copyright 2022. IL-TEM analysis of Pt/sulfur-doped carbon-1100(H_2_-treated) before (c) and after oxidation treatment (d). Reproduced from ref. 121 with permission from Elsevier, copyright 2022. Inverse FFT of InP/ZnSe/ZnS QDs before (e) and after (f) laser-exposure.^122^ Reproduced from ref. 122 with permission from Springer Nature, copyright 2024.

Platinum nanoparticles, a few nanometres in size, supported on carbon black, are often prepared using a wet impregnation method followed by annealing. This process results in the encapsulation of Pt nanoparticles within one or more layers of carbon shells due to the strong metal-support interaction (SMSI) effect. Through IL-TEM, the catalyst was examined to assess the effects of heating in air at 200 °C (Fig. 25c and d).^121^ The investigation revealed that even under these mild conditions, the carbon shells around the Pt nanoparticles were partially removed. This step is crucial for the application of Pt on carbon in the contexts of ORR and PEMFC, both relying on electrochemical access to the Pt surface.

The IL-TEM study of a laser heating process on quantum dots, which consist of a 3.6 nm indium phosphide (InP) core surrounded by a 2 nm thick zinc selenide (ZnSe) shell and a 0.2 nm zinc sulfide (ZnS) outer shell (InP/ZnSe/ZnS QDs), revealed that the shell material oxidised to form zinc oxide in the presence of oxygen.^122^ To minimise electron beam damage to these delicate materials, low-dose TEM imaging was employed, allowing for the investigation of individual quantum dots. The IL-TEM and inverse FFT data clearly demonstrated that oxidation results in distortions of the quantum dot's crystal lattice (Fig. 25e and f), which correlated with the degradation of their emission at 627 nm that was detected in bulk measurements.

### 5.4 Organic reactions in solution

Catalysis of organic reactions in solution involves intricate mechanisms, particularly for heterogeneous catalysts. In addition to the reactant-catalyst and support-catalyst interactions as in the gas phase, the solvent and co-reactants (such as a base in a cross-coupling reaction) also influence the evolution of catalytic centres. A significant question remains: does the reaction occur on the catalyst's surface or in the surrounding solution? IL-TEM methods can provide valuable insights by allowing researchers to track the fate of individual catalyst particles through imaging before and after the reaction. Graphitised carbon nanofibers (GNF), hollow carbon structures with nanoscale steps, have proven to be effective platforms for this purpose. They allow for precise tracking of nanoparticles in relation to the carbon steps. For example, the evolution of palladium nanoparticles, with an average diameter of 7 nm, formed directly on the steps of GNF from potassium tetrachloropalladate,^123^ has been evaluated in the Suzuki–Miyaura reaction of coupling of 4-iodonitrobenzene and phenyl boronic acid in methanol, using sodium acetate as a base at 70 °C over 24 h.^15^ IL-TEM enabled the tracking of distinct nanoparticles, demonstrating that they remain solid during the reaction (contradicting the common belief that they dissolve and re-precipitate). Instead, the primary mechanisms causing a decrease in the active surface area during the Suzuki–Miyaura reaction were shown to be nanoparticle migration and coalescence.

A different behaviour of palladium nanoparticles, sized between 0.5 and 2.0 nm, synthesised directly on oxidised multiwalled carbon nanotubes through the reduction of Pd salt in hydrogen, was observed during the cross-coupling of aryl iodides or bromides with phenylboronic acid, using K_2_CO_3_ as a base in a water/ethanol mixture over periods of 5, 10 min, or longer.^124^ IL-STEM and IL-HRTEM were conducted on the catalysts on a TEM grid before and after immersion in the reaction mixture. Initially, the flat surfaces of the as-prepared palladium nanoparticles became rough after 5 min of reaction time (Fig. 26a and b). After 10 min, a noticeable growth in nanoparticle size was observed, with their surfaces flattening once again. The nanoparticles rarely changed their positions, indicating minimal particle migration and coalescence. When the catalyst on the TEM grid was removed from the reaction solution immediately, without cooling, a significant dissolution of the palladium nanoparticles was noted, with their size decreasing from 1.6 nm to 0.9 nm. In contrast, allowing the catalyst to cool in the reaction mixture led to a considerable amount of precipitate forming on the surfaces of the catalyst particles, including newly formed palladium nanoparticles, KI, and biphenyl products. This observation supports the conclusion that the dissolution and precipitation of palladium nanoparticles play a crucial role in the reaction. Control experiments revealed that the base K_2_CO_3_ is essential for the dissolution process.

**Fig. 26:**
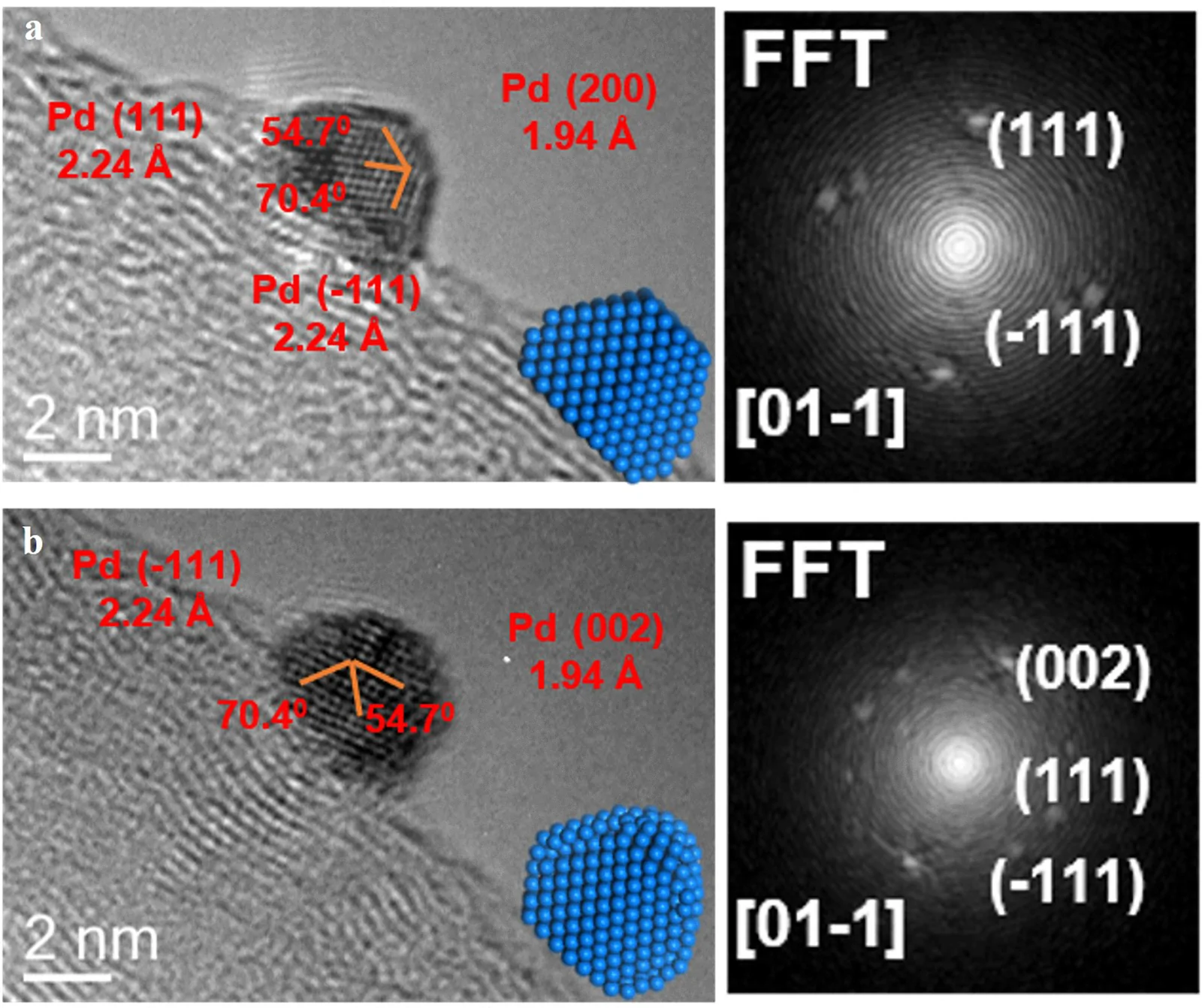
IL-HRTEM observation of Pd NPs supported on oxygen-functionalized carbon nanotubes before (a) and after (b) a Suzuki reaction with aryl iodide (iodobenzene) for 5 min. Adapted from ref. 124 with a figure provided by Wen Shi and Bingsen Zhang.

Palladium nanoparticles approximately 3 nm in size, formed through the spontaneous decomposition of an organometallic palladium complex in chloroform upon contact with a carbon support, were investigated using IL-SEM during the Mizoroki–Heck reaction of 4-iodo-nitrobenzene with styrene, in the presence of triethylamine, over 8 h at 140 °C. Additionally, IL-TEM of the palladium nanoparticles, supported on carbon-coated lacey formvar copper TEM grids, was conducted during the same reaction for 1 h at 100 °C.^125^ The study also employed IL-HAADF-STEM to observe the evolution of palladium nanoparticles during the Suzuki–Miyaura reaction between 4-iodo-nitrobenzene and 4-OMe-phenylboronic acid, which was carried out in the presence of K_2_CO_3_ in a water/ethanol mixture at 70 °C. Utilising three different identical-location approaches allowed for a detailed investigation of catalyst dynamics across varying length scales, from micro to nano and single-atom. Specifically, data from IL-SEM, processed using neural network analysis, indicated that the nanoparticles increased in size and were displaced from their original positions by up to 10 nm during the Mizoroki–Heck reaction. At the same time, IL-TEM observations did not reveal any removal of even the smallest nanoparticles from the carbon film; instead, only minor displacements were noted. Furthermore, IL-HAADF-STEM results after the Suzuki–Miyaura reaction indicated that single palladium atoms adsorbed onto the lacy carbon of the TEM grid changed positions, moving closer to a palladium nanoparticle. These comprehensive and insightful observations regarding the displacements of single atoms from the carbon film led the authors to conclude that the detachment and re-capture of single-atom palladium from and to the carbon support is a much more facile process than previously believed. This finding helps explain the high catalytic activity observed in bulk catalysis utilising this material.

### 5.5 Inorganic reactions in solution

Identical-location microscopy methods can effectively investigate solution-mediated inorganic reactions. For instance, the growth of UO_2_ phase on magnetite particles fixed on a SiN_*x*_ membrane was studied while immersed in a uranium chloride buffer solution for either 18 or 65 h using IL-HAADF-STEM.^126^ The identical-location imaging revealed the formation of UO_2_ nanowires from magnetite nanoparticles (Fig. 27a–f). Additionally, SAED and EDS spectroscopy confirmed the composition and structure of the nanowires as the UO_2_ phase. The detailed crystallographic identification was essential, as uranium exists in various oxo-forms. This study provides valuable insights into the evolution of uranium compounds under geological conditions.

**Fig. 27:**
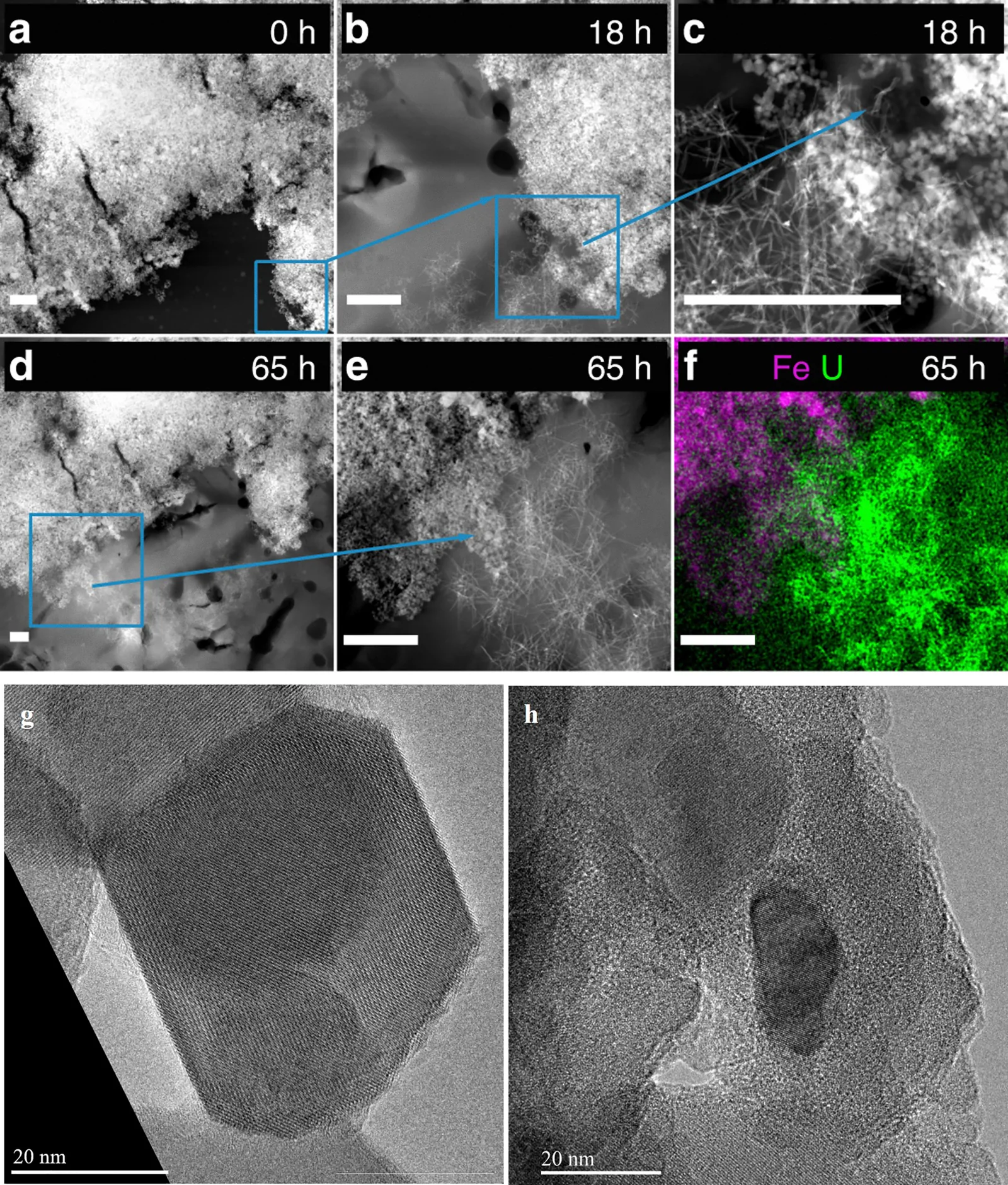
IL-HAADF-STEM observations of magnetite nanoparticles at different reaction times (a)–(e) (scale bar 500 nm). (f) STEM-EDS mapping of the region shown in (e). Reproduced from ref. 126 with permission from Springer Nature, copyright 2020. IL-HRTEM analysis of a forsterite nanoparticle before (g) and after (h) exposure to wet supercritical CO_2_ for 10 h. Adapted from ref. 128 with a figure provided by Xiaoxu Li.

Tamarack Bowl intrusion olivine with forsteritic composition (Mg_*x*_Fe_*y*_SiO_4_, where *x* and *y* denote the molar fractions of Mg and Fe, respectively, and *x* + *y* = 2) within a peridotite assemblage was examined by IL-TEM after exposure on TEM grids to water-saturated supercritical CO_2_ at 50 °C and 90 bar for 21 days.^127^ Imaging of identical regions before and after reaction revealed the development of a ∼4 nm amorphous surface layer on anorthite and a ∼10 nm altered layer on forsteritic olivine, accompanied by the appearance of ∼5 nm aragonite nanocrystals on the modified surface. These nanoscale features coincide with edge recession of olivine grains, indicating structural alteration at the mineral–fluid interface. Bulk XRD and TGA-MS measurements performed on reacted samples identified concurrent carbonate dissolution and precipitation trends under the same conditions.

Forsterite (magnesium silicate) particles measuring 20–60 nm were exposed to supercritical CO_2_, along with traces of water, at a pressure of 90 bar and a temperature of 50 °C for either 10 or 20 h.^128^ During this process, magnesium ions were leached from the particles. IL-TEM imaging (Fig. 27g–h) and EDS spectroscopy were used to monitor the dissolution and leaching of magnesium ions from the particle surfaces at different stages. This process resulted in the formation of an amorphous polysilicate shell, followed by a reaction of the magnesium ions and CO_2_ to form 2–5 nm carbonate nanocrystals that adhered to the shell. Understanding this process at the nanoscale is crucial for utilising basalt reservoirs for carbon dioxide capture and mineralisation, thereby helping to reduce this greenhouse gas.

### 5.6 Heat-activated processes in the absence of reactants

Heat-activated processes occurring at the interface between a catalyst and its support, even in the absence of reactant molecules, may significantly influence catalytic performance. A combination of IL-TEM and IL-HAADF-STEM has proven effective in elucidating the thermal evolution of this interface (Fig. 28a–d).^129^ In this study, a photocatalyst for water splitting was created using zinc oxide wurtzite phase wires, which had a diameter of 100–180 nm and were synthesised *via* a hydrothermal method. These wires were functionalised with zinc sulfide particles, ranging in size from 100–200 nm, that were grown using ion exchange. The material was heated in an inert environment at 400 °C for 10 min. This treatment led to the rotation of crystal grains of ZnS with respect to ZnO, thus enhancing the structural coherence of the ZnS–ZnO interface. The improved coherence of the interface significantly impacted the material's photophysical properties, resulting in increased photocurrent, reduced photo corrosion, and enhanced photocatalytic activity for hydrogen production from water. This improvement was attributed to lower barriers for the transport of photogenerated charge carriers, facilitated by the more structurally coherent ZnS–ZnO interface. In this study, identical-location measurements were bolstered by statistical ensemble-averaging analysis, both aiding the understanding of atomic-scale changes at the catalyst-support interface.

**Fig. 28:**
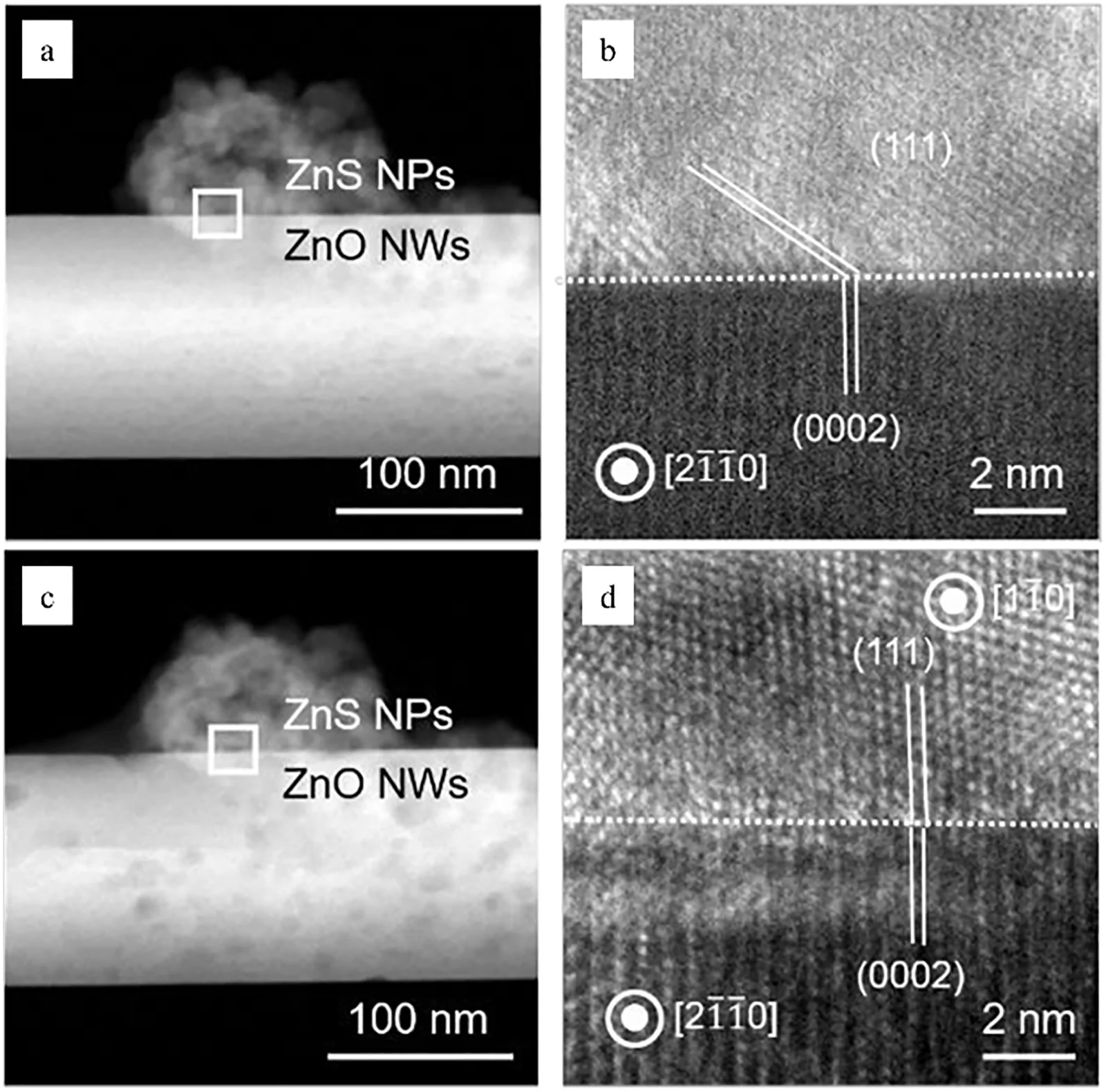
IL-HAADF-STEM and IL-HRTEM observations of the ZnO–ZnS interface before (a) and (b) and after (c) and (d) thermal annealing. Reproduced from ref. 129 with permission from Elsevier, copyright 2022.

### 5.7 Dehydrogenation

Selective propane dehydrogenation (PDH) plays a vital role in the industrial production of propylene, a crucial building block for the polymer and petrochemical sectors. Gaining insight into the structural and compositional evolution of PDH catalysts is critical for guiding the design of more efficient and durable catalytic materials. IL-SEM and IL-EDS analyses were conducted on Ga–Pt alloys synthesised *via* ultrasonication followed by galvanic displacement.^130^ Remarkably, the overall particle morphology remained unchanged throughout the entire sequence of reaction steps, but notable changes were observed in the distribution of Pt. In the pristine state, Pt was localised in specific regions on the particle surface. Upon H_2_ pretreatment at 823 K, Pt appeared to dissolve into the droplets. After the dehydrogenation reaction at 823 K for 15 h, a further redistribution of Pt was detected, suggesting Pt dissolution during the catalytic process. This identical-location approach provides valuable insights into the morphological and compositional evolution of catalysts under realistic reaction conditions.

## 6. Methodological challenges and opportunities

For IL-EM to be successful, the catalyst must exist in arrested states between the reaction steps. This naturally defines the scope of catalytic reactions for IL-EM as those with high activation barriers, so that the reaction proceeds only at high temperature or under an applied potential, and all dynamics cease as soon as the temperature drops or the potential is turned off. The latter is particularly convenient for trapping the catalyst in arrested states, which explains a significant preference for electrocatalysis in the IL-EM literature, with relatively few thermally catalysed reactions and even fewer liquid reactions reported to date. Instead of the spatiotemporally continuous evolution of catalyst particles obtained by *in situ* microscopy, IL-EM offers a different perspective on catalyst dynamics – a series of snapshots – imposing temporal resolution as one of the key limitations of IL-EM. However, if the stages of the reaction are chosen wisely for IL-EM, for example, at critical moments such as catalyst activation, change of selectivity, or onset of deactivation, we can convincingly reconstruct the entire process by interpolating the evolution dynamics between the static images, thanks to IL-EM taking us to the same particle at each stage.

A major practical hurdle of IL-EM is the substantial time and effort required for repeated sample transfer, relocation, reaction exposure and imaging. The finder grid must be manually moved from the microscope to a reactor and back several times. This process involves evacuating or filling the setup with gases or liquids, adjusting the temperature, attaching and detaching the sample grid to electrodes, and transporting the sample grid between different laboratories. The failure rate in this series of steps is high due to potential human errors and the risk of damaging the sample grid. If a mistake occurs, there is no way to revert to a previous step, necessitating a complete restart of the entire process. Additionally, exposing the sample to ambient conditions between imaging steps limits the types of catalysts that can be used in IL-EM, as only those reasonably stable in air can be employed unless the sample is transferred without exposure to air between reaction and microscope. Future developments in interfacing an electron microscope directly with a reaction chamber and automating the transfer process using robotics could significantly enhance the utility of IL-EM by saving time and effort, improving the success rate of experiments, and protecting catalysts from contamination or oxidation in air. A related potential future direction is to keep the catalyst sample within a reaction TEM cell but remove or exchange the reactive medium just before imaging, thereby reducing beam–medium interactions, such as radiolysis, while retaining identical-location tracking. If selecting such an approach, however, it is important to consider how the material of the cell windows can affect image contrast and the quality of *in situ* spectroscopy, as both may degrade information and resolution.

It is noteworthy that most studies involving IL-EM primarily focus on catalyst degradation. This emphasis can be attributed to the longstanding tradition of using EM for post-mortem analysis of catalysts to determine why they failed. However, there is considerable potential for IL-EM to be used to investigate other important questions, such as the mechanisms of catalyst activation or changes in selectivity. Addressing these questions could greatly inform the design and fabrication of catalysts, maximising their effectiveness in heterogeneous processes. Despite this potential, the number of studies exploring these aspects remains relatively small.

Atomically-resolved IL-EM data are still quite rare. This scarcity may be attributed to the complexity and significant effort required for IL-EM, as well as a higher failure rate in experiments, as previously discussed. The choice of catalyst support could also influence outcomes; low-contrast 1D or 2D supports, such as nanotubes or graphene, might be more effective for obtaining atomically resolved images compared to 3D support particles that have high contrast or non-uniform morphology. Additionally, selecting thinner supports or those with a cylindrical shape can facilitate the extraction of three-dimensional information about catalyst particle evolution. This can be achieved through EM tomography or through-focus image reconstruction techniques.

In thermally activated reaction media, gas-phase reactions are clearly more dominant in IL-EM measurements than in liquid media. This is likely because incorporating a finder grid into gas reactors is easier and cleaner. Liquid solvents, on the other hand, can contaminate the catalyst or create artefacts during drying due to surface tension effects. To date, there have been no reported instances of thermally activated reactions in liquid flow systems. There is significant potential to develop IL-EM for liquid-flow reactors, as many catalytic reactions essential to fine organic synthesis, such as pharmaceutical production, occur under these conditions. IL-EM could provide valuable insights into catalyst evolution in these important processes.

The next major challenge for IL-EM, like all microscopy methods, is the inherent issue of statistical significance in the acquired data. AI-assisted automation of image acquisition and processing may increase the throughput of EM, including IL-EM. For instance, machine learning algorithms can be trained to recognise specific changes in particle shapes, sizes, interparticle distances, and features of the particle-support interface, with some early examples of AI use in IL-EM already emerging.^23^ This approach can facilitate automated decision-making for classifying single-particle events (SPE), helping eliminate human bias and increase data volume. Because IL-EM follows the same catalyst particle through successive reaction stages, the evolution pathway of each particle can be established directly while retaining information on its individual size, structure and local nano-landscape (*e.g.* features of support materials in the locality of the catalyst particle), rather than being inferred from averaged changes across non-identical particles. This creates an opportunity to use statistics differently in IL-EM: particle-specific trajectories reveal mechanistic pathways (SPE_*i*_, Fig. 1c), and larger datasets of such trajectories quantify how frequently different pathways occur across a catalyst population (*n*_*i*_, Fig. 1c). It has been recently shown that indiscriminate averaging of IL-EM trajectories can obscure particle-specific mechanisms relevant to macroscopic catalyst performance, whereas classifying and statistically analysing many individual trajectories can link nanoscale heterogeneity to bulk catalytic behaviour.^6^ Therefore, in large IL-EM datasets, the key is classification of particle trajectories into distinct evolution pathways, such as dissolution, redeposition, coalescence, Ostwald ripening, phase transformation, alloying/dealloying, faceting, fragmentation or apparent stability. The relative frequency of these pathways can then be compared with particle size, composition, support morphology, local particle density and catalytic performance. This would allow IL-EM to move beyond illustrative before/after examples and towards a quantitative description of catalyst heterogeneity, identifying which single-particle events are dominant, which are rare, and which are most strongly linked to changes in activity, selectivity or stability.

Feeding into the same challenge, correlation of IL-EM data with bulk analysis techniques is a particularly fruitful direction for the future. The identical-location workflows lend themselves well to such correlations, with many examples of IL-EM coupled with Raman, XPS, CV, ICP, and other bulk techniques described in this review. These examples clearly illustrate that structural (*e.g.*, size, shape, texture) and chemical (*e.g.*, alloying, leaching, oxidation, *etc.*) changes at the individual particle level can be effectively used to rationalise the spectroscopic or electrochemical signal from the macroscopic sample, *e.g.*, the entire TEM grid. Nevertheless, there are significant opportunities to develop methods of particular relevance to heterogeneous catalysis for correlation with IL-EM, such as near-ambient-pressure X-ray photoelectron spectroscopy (NAP-XPS), XAS, and diffuse reflectance infrared Fourier-transform spectroscopy (DRIFTS), that would strengthen the structure–property relationship in the context of IL-EM even further.

In this context, it must be noted that SEM is intrinsically higher-throughput (hence provides a better representation of the macroscopic sample) but less informative for SPE than TEM. Recently, a workflow for correlative SEM-AC-STEM imaging at the same location has been reported, which may offer another route to improving throughput and overcoming the lack of statistics in IL-EM, with SEM providing statistical information and AC-STEM providing atomic-resolution imaging. In this work, HAADF-STEM-in-SEM has been successfully benchmarked against AC-STEM at identical locations, thereby calibrating STEM-in-SEM image intensities to AC-STEM-derived atom counts for ultrasmall metal nanoclusters. In this example, AC-STEM provides the high-precision reference, while STEM-in-SEM enables faster navigation and more efficient imaging over larger sample areas.^20^

Finally, a major opportunity lies in the multimodal correlation of local-probe methods that combine structure, composition, and function into a seamless characterisation workflow at the single-particle level. As demonstrated by numerous examples in this review, correlative EELS and EDS can link morphological evolution to oxidation state and compositional changes within the same particle. There is a significant opportunity to extend this strategy to IL-4D-STEM, which can provide information on local crystallography, strain, and phase evolution, while scanning electrochemical cell microscopy (SECCM) and related nanoscale electrochemical approaches could, in principle, correlate structural trajectories with local electrochemical activity. Importantly, emerging quantum sensing techniques, such as optically detected magnetic resonance (ODMR), can now be carried out at the single-particle level, on a TEM finder grid, and therefore link functional magnetic measurements in the material directly with structural and chemical changes taking place in the specific particle under various conditions.^131–133^ A combination of EM and local magnetic sensing could open the door for studying mechanisms of catalytic reactions proceeding *via* free-radical intermediates.^134^ Combined with automated relocation and AI-assisted image analysis, these methods could make it possible to ask not only what happened to a catalyst particle, but which particles changed and why, how often each pathway occurred, thereby ultimately linking the local chemistry of a catalytic particle to its catalytic function.

## 7. Summary and outlook

Electron microscopy originated as a materials science and engineering tool to examine static structures, and its use within the chemistry research community continues to expand as one of the most powerful tools for examining the transformations of particles and molecules and the dynamics of surfaces and single atoms. The aberration corrector allowed for a crossing of the 1 Å resolution threshold, meaning that the spatial resolution exceeds the length of any chemical bond, enabling direct visualisation of atoms and their dynamics. The levels of chemical information obtained from EM are now not as much limited by the capabilities of the microscope as by the stability of the material under the electron beam. As approaches to mitigating beam damage are being developed, including the latest innovations in identical-location electron microscopy, IL-EM is now firmly within the arsenal of analytical methods for chemistry, alongside traditional bulk characterisation methods, such as spectroscopy and diffractometry. While the bulk analysis methods record the signal of billions of particles in each measurement, IL-EM imaging and analysis track the evolution of individual catalyst particles, thus preserving the particle identity, local nanoscale landscape, and the specific characteristics of each particle, preventing the loss of vital information that can occur through ensemble averaging. IL-EM requires no specialised microscopy infrastructure, which is needed for *in situ* or environmental EM, making it more accessible to the chemistry research community from a technical perspective, albeit more labour-intensive. Recent developments in correlative identical-location imaging and AI-assisted high-throughput analysis are further expanding the capabilities and scope of IL-EM for characterising heterogeneous catalysts.

## Conflicts of interest

There are no conflicts to declare.

## Abbreviations

Abbreviation: Definition
4D-STEM: Four-dimensional scanning transmission electron microscopy
AC-STEM: Aberration-corrected scanning transmission electron microscopy
ADP: Accelerated degradation protocol
ADT: Accelerated durability test
AEM: Anion-exchange membrane
AI: Artificial intelligence
AST: Accelerated stress test
ATR-FTIR: Attenuated total reflectance Fourier-transform infrared spectroscopy
BOL: Beginning of life
BSCF: Ba_0.5_Sr_0.5_Co_0.8_Fe_0.2_O_3−*δ*_
CCM: Catalyst-coated membrane
CL: Catalyst layer
CNE: Carbon nanoelectrode
CNT: Carbon nanotube
CO_2_RR: Electrochemical carbon dioxide reduction reaction
CV: Cyclic voltammetry
DHBT: Dynamic hydrogen bubble template
DRIFTS: Diffuse reflectance infrared Fourier-transform spectroscopy
ECSA: Electrochemically active surface area
EDS: Energy-dispersive X-ray spectroscopy
EELS: Electron energy-loss spectroscopy
EM: Electron microscopy
ETEM: Environmental transmission electron microscopy
FAOR: Formic acid oxidation reaction
FE: Faradaic efficiency
FFT: Fast Fourier transform
FTIR: Fourier-transform infrared spectroscopy
GC: Gas chromatography
GDE: Gas diffusion electrode
GDL: Gas diffusion layer
GNF: Graphitised carbon nanofibre
GPS: Global positioning system
HAADF-STEM: High-angle annular dark-field scanning transmission electron microscopy
HER: Hydrogen evolution reaction
HGS: Hollow graphitic spheres
HMCS: Hollow mesoporous carbon spheres
HRTEM: High-resolution transmission electron microscopy
HSC: High-surface-area carbon
ICP: Inductively coupled plasma
ICP-MS: Inductively coupled plasma mass spectrometry
ICP-OES: Inductively coupled plasma optical emission spectroscopy
IL: Identical-location; used as a prefix for an imaging or spectroscopy method
IL-EM: Identical-location electron microscopy
IL-SEM: Identical-location scanning electron microscopy
IL-STEM: Identical-location scanning transmission electron microscopy
IL-TEM: Identical-location transmission electron microscopy
ILIAS: Identical-location imaging and spectroscopy
LDH: Layered double hydroxide
MEMS: Microelectromechanical systems
ML: Monolayer
NAP-XPS: Near-ambient-pressure X-ray photoelectron spectroscopy
OCP: Open-circuit potential
ODMR: Optically detected magnetic resonance
OER: Oxygen evolution reaction
ORR: Oxygen reduction reaction
PDH: Propane dehydrogenation
PEMFC: Proton-exchange membrane fuel cell
RDE: Rotating disk electrode
RHE: Reversible hydrogen electrode
SAED: Selected-area electron diffraction
SAXS: Small-angle X-ray scattering
SE: Secondary electron
SECCM: Scanning electrochemical cell microscopy
SFC-ICP-MS: Scanning flow cell inductively coupled plasma mass spectrometry
SMSI: Strong metal–support interaction
SOR: Solketal oxidation reaction
SPE: Single-particle event
STEM: Scanning transmission electron microscopy
STEM-in-SEM: Scanning transmission electron microscopy in a scanning electron microscope
SUSD: Start-up/shut-down
TAP: Tris(aza)pentacene
TEM: Transmission electron microscopy
TGA-MS: Thermogravimetric analysis-mass spectrometry
ToF: Turnover frequency
UME: Ultramicroelectrode
UPD: Underpotential deposition
URFC: Unitised regenerative fuel cell
UV: Ultraviolet
WAXS: Wide-angle X-ray scattering
XAS: X-ray absorption spectroscopy
XPS: X-ray photoelectron spectroscopy
XRD: X-ray diffraction
ZIF: Zeolitic imidazolate framework

## Data Availability

No primary research results, software or codes have been included, and no new data were generated or analysed as part of this review.
